# Versatile regulation of effectors by novel orthologous regulators in the *Legionella* genus

**DOI:** 10.1128/mbio.01268-25

**Published:** 2025-05-30

**Authors:** Chen Adler, Gil Segal

**Affiliations:** 1 The Shmunis School of Biomedicine and Cancer Research, George S. Wise Faculty of Life Sciences, Tel Aviv University 26745, Tel Aviv-Yafo, Israel; The University of Texas Health Science Center at Houston, Houstan, Texas, USA

**Keywords:** *Legionella*, effectors, gene expression, LTTR, local regulator, global regulator

## Abstract

The intracellular pathogen *Legionella pneumophila* utilizes the Icm/Dot type IVB secretion system, which translocates >300 effector proteins into host cells. The expression of the effector-encoding genes (EEGs) is mediated by a combination of global and local transcriptional regulators. Here, we describe two novel LysR-type transcriptional regulators (LTTRs), LelB (lpg2138) and LelC (lpg1796), which function as local regulators of EEG in *L. pneumophila*. Bioinformatic analysis, expression assays, site-directed mutagenesis, and *in vitro* binding experiments led to the discovery that in *L. pneumophila,* LelB controls the expression of three EEGs, and LelC controls the expression of a single EEG. Moreover, we discovered a versatile regulator, LelB, that can serve either as a global or local regulator of EEGs. The LelB orthologs in the *Legionella* genus are divided into three clades, but only in two of them *lelB* is located adjacent to its target genes and functions as a local regulator. A genomic search using the validated LelB regulatory element identified multiple genes (between 19 and 36) harboring the LelB regulatory element in the five *Legionella* species belonging to the third clade. Examination of this collection of genes uncovered that most of them harbor common effector domains and other known effector features. Furthermore, experimental analysis of these genes from one of these species, *L. longbeachae*, indicated that LelB regulates their expression. Our results demonstrate that LelB functions as a local or global regulator of EEGs in different *Legionella* species, thus uncovering the remarkable versatility of effector regulation by LTTRs in the *Legionella* genus.

**IMPORTANCE:**

*Legionella pneumophila* translocates into host cells the largest number of effector proteins known in any pathogen. This cohort of proteins needs to be coordinated at the gene expression level to result in a successful infection. To date, the regulation of effector-encoding genes in *L. pneumophila* has been found to be mediated by global regulators that control the expression of multiple effectors and local regulators that control the expression of a single or few effectors. Here, we identified two novel regulators that function as local regulators of effectors in *L. pneumophila*. However, analysis of their orthologs across the *Legionella* genus revealed that one of them controls the expression of multiple genes in five *Legionella* species. The properties of the proteins encoded by these genes suggest that most of them function as effectors. Our data demonstrate a versatile regulator whose orthologs function as local or global regulators of EEGs in different *Legionella* species.

## INTRODUCTION

*Legionella pneumophila* is an intracellular human pathogen that multiplies within alveolar macrophages and causes severe pneumonia known as Legionnaires’ disease (1, 2). In the environment, *L. pneumophila* thrives in numerous different protozoan species (3, 4). Inside its eukaryotic hosts, *L. pneumophila* remodels its phagosome to generate the *L. pneumophila*-containing vacuole (LCV) (5, 6). The establishment of the LCV depends on effectors’ translocation by the Icm/Dot type IV secretion system (7, 8). The *L. pneumophila* Icm/Dot secretion system delivers more than 300 effector proteins that modulate host cell functions during infection (reviewed in References (9–13)), and thousands of effectors were predicted throughout the *Legionella* genus (14, 15). The study of *Legionella* effectors has revealed that many of these effectors share similar properties, including (i) the presence of eukaryotic protein domains, such as ankyrin repeats, serine-threonine kinase domains, F-box, and U-box ubiquitin E3 ligase domains, to name a few (13, 14, 16); (ii) the presence of a type IV secretion signal at their C-terminal (17–19), (iii) effector-encoding genes (EEGs) tend to cluster together in specific genomic regions (20–22), (iv) many EEGs have lower GC content compared to the rest of the genome, probably due to horizontal gene transfer (HGT) (20, 23), and (v) many EEGs are co-regulated at the transcriptional or the translational levels.

The enormous number of *L. pneumophila* effectors that participate in the manipulation of many host-cell processes during infection implies that a successful infection requires coordination among effectors on various levels, including gene expression. The study of *L. pneumophila* EEG regulation has revealed a multi-component, interconnected regulatory network composed of global and local direct regulators, orchestrating the expression of *L. pneumophila* EEGs.

The *L. pneumophila* global regulatory systems are conserved in all the *Legionella* species examined. They are usually located at the same genomic locus, and they directly regulate the expression of many EEGs located throughout the *L. pneumophila* genome. Thus far, these regulatory systems include (i) the PmrAB two-component system (TCS), which includes the PmrA response regulator (RR) and the PmrB sensor-histidine kinase (SHK), which directly activate the expression of 42 EEGs (24, 25). (ii) The CpxRA TCS, which includes the CpxR RR and the CpxA SHK, was shown to directly activate or repress the expression of 26 EEGs and 4 *icm/dot* genes (26–29). (iii) The LetAS-RsmYZ-CsrA regulatory cascade, which includes the LetA RR, the LetS SHK, the two small RNAs (sRNAs) RsmY and RsmZ, and the CsrA repressor, which posttranscriptionally inhibits the translation of more than 40 EEGs (30–37). (iv) Two FIS regulators that directly repress the expression of about 30 EEGs (38–41). One additional global regulator, Fur, is also involved in EEG expression, but unlike the other global regulators, it regulates only a single effector, as well as other genes involved in iron acquisition (42–45).

Besides the global regulatory systems described above, some *L. pneumophila* EEGs were found to be directly regulated by local regulators. These regulators are present only in a few *Legionella* species, they undergo HGT within the *Legionella* genus, and they typically regulate the expression of one or two EEGs located adjacent to them. In some cases, they also regulate the expression of a few distantly located EEGs (39–41). Thus far, the known *L. pneumophila* local regulatory systems are as follows: (i) The LciRS TCS, which includes the LciR RR and the LciS SHK, is activated by copper and controls the expression of a single adjacent EEG located in the same genomic island (39). (ii) RegK3, a LuxR family regulator, which controls the expression of two adjacent EEGs located in the same genomic island (40). (iii) LelA, a LysR-type transcriptional regulator (LTTR), which controls the expression of three EEGs, one positioned adjacent to it, and two additional EEGs located distantly (41). The first two local regulatory systems described (LciRS and RegK3) regulate the same EEGs in all sequenced *Legionella* species and were found as a “regulator-effector island,” whereas the third local regulator (LelA) controls the expression of a variety of adjacently located EEGs or putative EEGs in the *Legionella* species in which it was found, as well as a few genes located distantly.

All these global and local direct regulators of EEGs constitute a highly interconnected regulatory network. Direct regulators of EEGs were shown to regulate the expression of one another: PmrA controls the expression of *csrA* (33), and the Fis repressors strongly repress the expression of two of the local regulators LciR and RegK3 (39, 40). In addition, the RpoS sigma factor was shown to participate in the regulation of the RsmY and RsmZ sRNAs, which are part of the global LetAS-RsmYZ-CsrA regulatory cascade (37, 46), and it also regulates the expression of the local regulator LelA (41). Furthermore, accessory components such as LetE, LerC, and the nitrogen phosphotransferase system (PTS^Ntr^) connect and coordinate the EEG regulatory systems (28, 47–49).

Further exploring this large and interconnected EEG regulatory network, we hypothesized that additional *L. pneumophila* LTTRs might function as local regulators of EEGs. We found that orthologs of the *L. pneumophila* LTTRs lpg2138 and lpg1796 are present in several *Legionella* species, and most of them are located adjacent to EEGs or putative EEGs. Bioinformatic and experimental analyses revealed that lpg2138 (LelB) and lpg1796 (LelC) directly regulate the expression of EEGs in *L. pneumophila*. We further found that in five *Legionella* species harboring a LelB ortholog, the LelB regulatory element is found upstream of numerous putative EEGs located throughout their genomes. Examination of 10 of these genes from *L. longbeachae* indicated that they are indeed regulated by LelB. These results indicate that LelB functions as a local regulator of EEGs in several *Legionella* species and as a global regulator of EEGs in other *Legionella* species.

## RESULTS

### The distribution of lpg2138 LTTR orthologs in the *Legionella* genus

**Fig 1:**
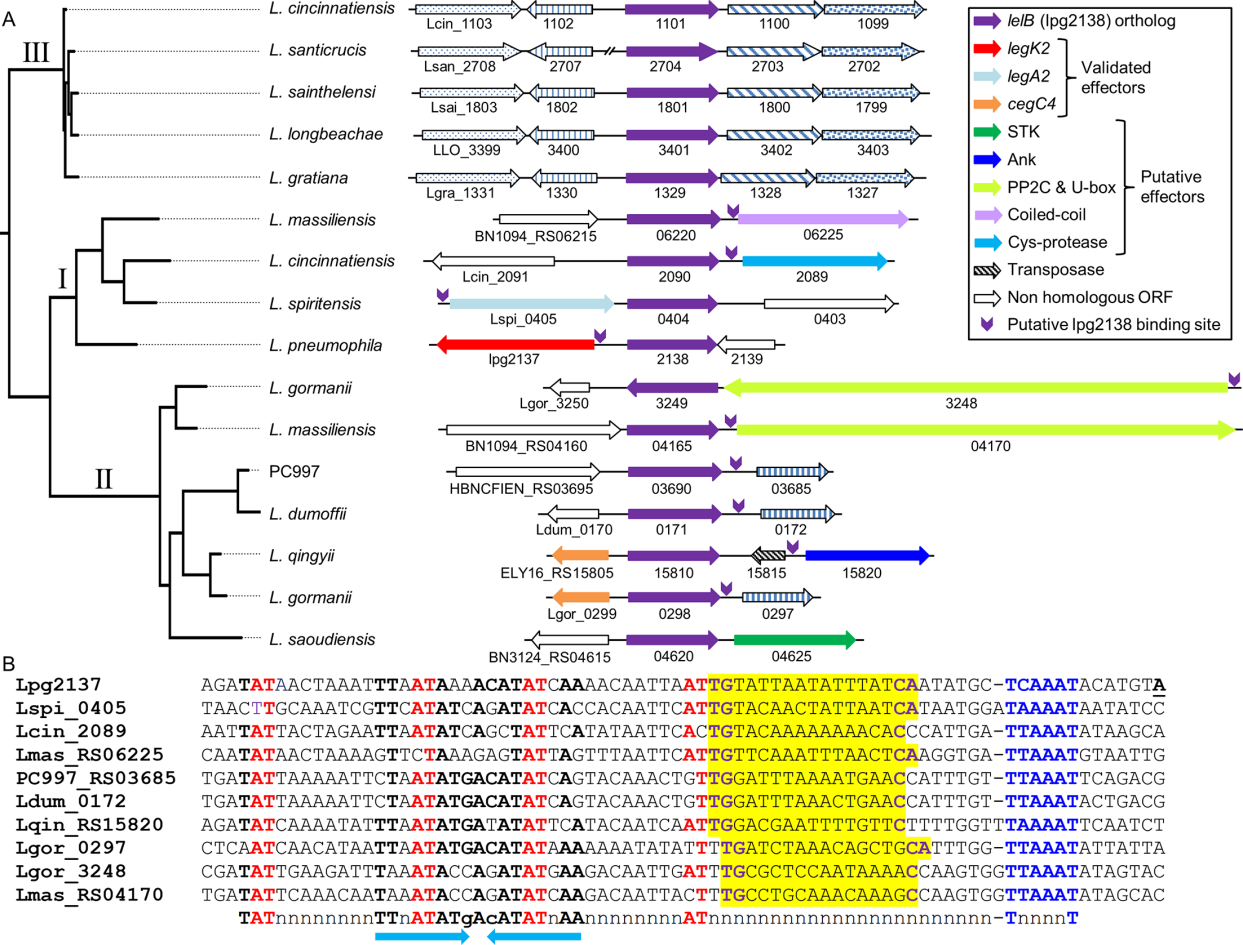
Distribution of lpg2138 (*lelB*) in the *Legionella* genus. (A) Schematic representation of the genes located in the genomic region adjacent to the lpg2138 (*lelB*) LTTR orthologs in *Legionella* species harboring the regulator. In three *Legionella* species (*L. cincinnatiensis*, *L. massiliensis*, and *L. gormanii*), two copies of the regulator were found in different genomic locations. Homologous genes are marked by the same color or pattern, and non-homologous genes are marked in white. The genes are indicated by their locus tag number. The position of the conserved regulatory element predicted to be recognized by LelB is marked by purple arrows pointing downward. The maximum-likelihood phylogeny tree on the left was reconstructed using the amino acid alignment of the lpg2138 orthologous ORFs. (B) The regulatory regions of genes located adjacent to lpg2138 and harbor a conserved regulatory element. The putative −10 promoter element is in blue, the nucleotides representing the common LTTR motif are in red, the putative Fis regulatory elements are shaded in yellow, and their conserved nucleotides are in purple; other conserved nucleotides are in bold, and the inverted repeat sequence is marked by arrows. The genes are indicated by their locus tag numbers. The prefix of the locus tags is the following: *L. massiliensis* - BN1094_RS# (Lmas), *L. qingyii* - ELY16_RS# (Lqin), and the uncharacterized species PC997 - HBNCFIEN_RS# (PC997).

The notion that some of the *L. pneumophila* EEGs transcriptional regulators are located adjacent to their target genes (39–41) led us to seek additional such regulatory systems. This resulted in the identification of the LTTR lpg2138 and the EEG *legK2* (lpg2137) (50) located adjacent in the *L. pneumophila* genome. To gain insights into the lpg2138 orthologs and their putative target genes, we examined all the fully sequenced *Legionella* species for close orthologs of lpg2138. Close orthologs of lpg2138 (with at least 45% identity, 65% similarity in amino acids, and a BLAST *e* value of ≥8e^−86^) were found in 12 characterized *Legionella* species and one uncharacterized *Legionella* species (Fig. 1A). In three *Legionella* species (*L. cincinnatiensis*, *L. massiliensis*, and *L. gormanii*), two paralogs of the regulator were found. The lpg2138 orthologs were divided into three clades according to their protein sequence. Clades I and II include 11 orthologs, most of which are located adjacent to genes encoding known effectors or proteins that harbor common effector domains (14, 16, 51, 52). Clade III is comprised of orthologs from five *Legionella* species, all of which are located at similar genomic positions (Fig. 1A), and the genes located adjacent to them encode proteins that seem unrelated to pathogenesis (the *L. longbeachae* genes and their orthologs encode: LLO_3399 - succinylglutamate desuccinylase; LLO_3400 - ubiquinone biosynthesis C-methylase; LLO_3402 - NAD-dependent epimerase; and LLO_3403 - ornithine cyclodeaminase). Careful examination of the upstream regulatory regions of the genes located adjacent to the lpg2138 orthologs in clades I and II led us to identify in most of them a putative regulatory element located at a similar distance from their putative −10 promoter element (Fig. 1). This conserved regulatory element constitutes a typical LTTR regulatory element (53–56) harboring the sequence motif AT-N_11_-AT-N_7_-AT-N_11_-AT and containing additional conserved positions which form an inverted-repeat sequence between the conserved ATs (Fig. 1B). No such regulatory element was identified in the genes located adjacent to clade III orthologs (clade III is described below in more detail). Following these and the experimental results presented hereafter, lpg2138 was named LelB for *Legionella* EEGs (effector-encoding genes) LTTR (LysR-type transcription regulator). LelA was the first *Legionella* LTTR that was found previously to regulate the expression of EEGs (41).

### *L. pneumophila* LelB regulates the expression of three EEGs

**Fig 2:**
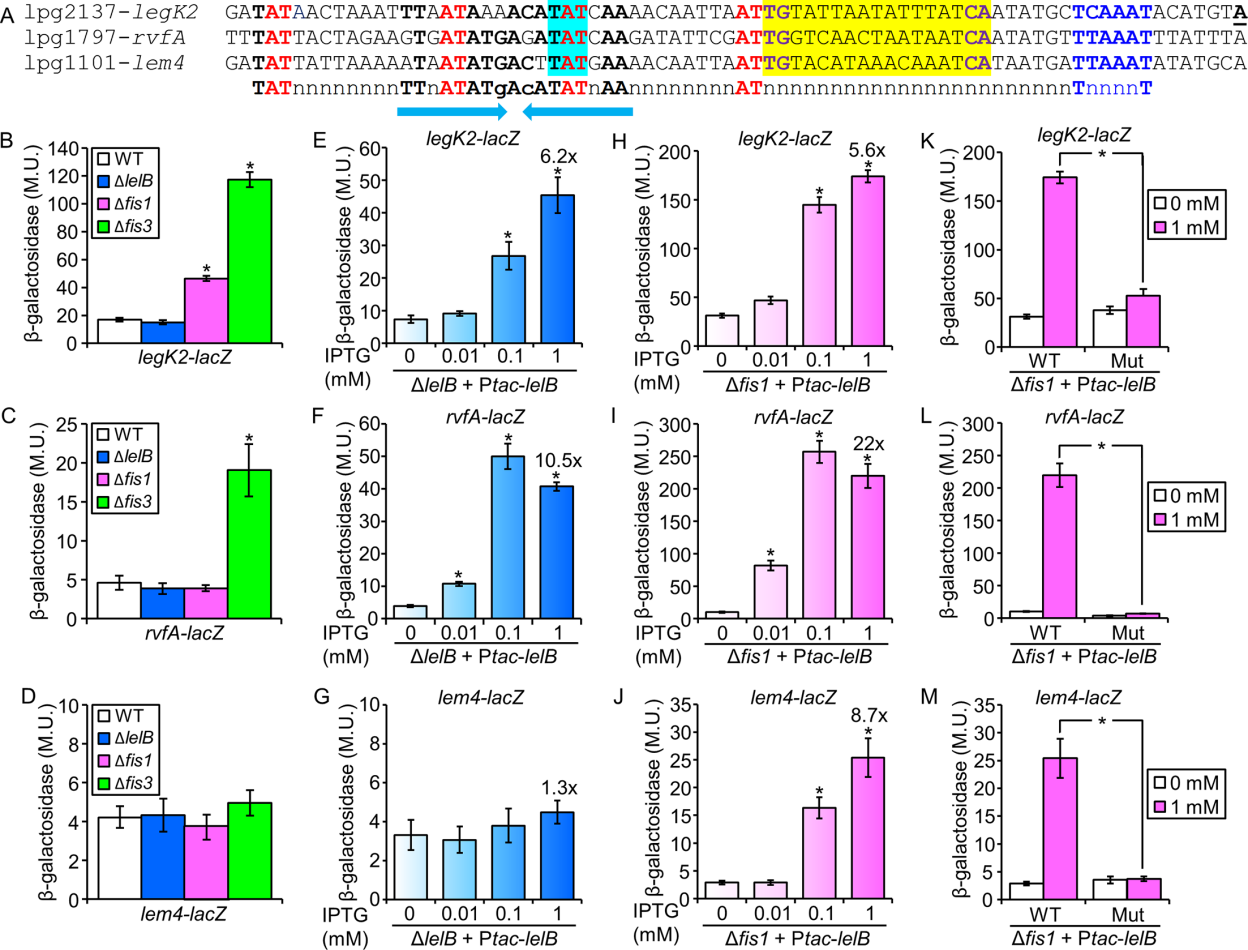
LelB activates the expression of three *L. pneumophila* EEGs. (A) The regulatory regions of three *L. pneumophila* EEGs harbor the putative LelB conserved regulatory element. The putative −10 promoter elements are in blue, the nucleotides representing the common LTTR motif are in red, the putative Fis regulatory elements are shaded in yellow and their conserved nucleotides are in purple, and the nucleotides shaded in cyan were mutated (TAT to ATA); other conserved nucleotides are in bold, and the inverted-repeat sequence is marked by arrows. (B–D) Expression of *legK2* (**B**), *rvfA* (**C**), and *lem4* (**D**) *lacZ* fusions was examined in wild-type *L. pneumophila* and the *lelB, fis1*, and *fis3* deletion mutants. The levels of expression of the *lacZ* fusions were found to be significantly different (*P < 10^−4^, unpaired Student’s *t*-test) between expression levels of the same *lacZ* fusion in the deletion mutants and those in the wild-type strain. (E–J). The expression of *legK2* (**E, H**), *rvfA* (**F, I**), and *lem4* (**G, J**) *lacZ* fusions was examined in *L. pneumophila* containing a deletion in *lelB* (**E–G**) and *fis1* (**H–J**). The bacteria examined contained a plasmid with the *L. pneumophila lelB* gene cloned under the control of the P*_tac_* promoter (activated by IPTG), and they were grown in medium containing different concentrations of IPTG (indicated below the bars). The levels of expression of the *lacZ* fusions were found to be significantly different (*P < 10^−4^, unpaired Student’s *t*-test) between expression levels of the same *lacZ* fusions examined without IPTG and with different IPTG concentrations. (K-M). LelB requires its putative regulatory element to activate the expression of its target genes. The expression of wild-type *lacZ* fusions (WT) of *legK2* (**K**), *rvfA* (**L**), and *lem4* (**M**), and the same fusions containing a mutation (Mut) in the suspected LelB regulatory element were examined in the *L. pneumophila fis1* deletion mutant. The bacteria examined contained a plasmid with the *L. pneumophila lelB* gene cloned under the control of the P*_tac_* promoter (activated by IPTG), and they were grown with (1 mM) and without IPTG. The levels of expression of the wild-type and mutated *lacZ* fusions were found to be significantly different (*P < 10^−4^, unpaired Student’s *t*-test) between the fusions examined with IPTG. β-Galactosidase activity was measured as described in the Materials and Methods. Data (expressed in Miller units [M.U.]) represent the average ± standard deviations (error bars) from at least three different biological replicates.

The identification of the conserved putative regulatory element described above (Fig. 1B) made it possible to search for it in the *L. pneumophila* genomic sequence. This analysis revealed that three *L. pneumophila* EEGs (*legK2*, *rvfA*, and *lem4*) harbor a highly similar regulatory element (Fig. 2A). To determine whether LelB regulates the expression levels of these three EEGs, we constructed a deletion mutant of the *lelB* gene. Examining this deletion mutant in *Acanthamoeba castellanii*, a natural amoeba host of *Legionella*, indicated that the *lelB* gene is dispensable for intracellular growth in this host cell (Fig. S1), and similar results were also reported in other host cells (57). Next, we examined the expression levels of these three EEGs-*lacZ* fusions in the *L. pneumophila* wild-type strain in comparison to their expression in the *lelB* deletion mutant (Fig. 2B through D), and no effect was observed. The low expression levels of these three EEGs might suggest that they are subjected to Fis repression, as was previously shown for numerous EEGs (38–41). Therefore, we examined the expression levels of these three EEGs in the *fis1* (lpg0542) and *fis3* (lpg1743) deletion mutants (Fig. 2B through D, a double deletion of *fis1* and *fis3* is non-viable). Two of the three EEGs had higher expression levels in the *fis1* and *fis3* deletion mutants, indicating that indeed the Fis proteins repress their expression and might hinder our ability to observe the LelB effect on their expression. These results led us to use an inducible expression system to examine the LelB regulation of the three EEGs. In this system, the *lelB* gene was cloned under the regulation of the P*_tac_* promoter (induced by IPTG), which was introduced into the *L. pneumophila lelB* deletion mutant together with the three EEGs-*lacZ* fusions, individually. Using this analysis, two of the three EEGs (*legK2* and *rvfA*) showed dose-dependent expression levels as the concentration of IPTG (controlling the expression level of the *lelB* gene) was increased (Fig. 2E through G). Since the expression levels of *lem4* were not affected by both the overexpression of LelB and the *fis1* and *fis3* deletion mutants, we considered the possibility that both Fis1 and Fis3 strongly repress its expression (as was previously shown with other EEGs (38–41)). To this end, we examined the three EEGs for activation by LelB in the *fis1* and *fis3* deletion mutants (Fig. 2H through J; Fig. S2A). In both *fis* deletion mutants, *lem4* was activated by LelB (compare Fig. 2G and J; Fig. S2A), and *rvfA* demonstrated higher expression levels compared to the *lelB* deletion mutant (compare Fig. 2F and I; Fig. S2A), indicating that relieving Fis repression allows LelB to better activate these EEGs. Therefore, we concluded that the LelB regulator activates the expression level of three *L. pneumophila* EEGs, indicating that it might function as their direct positive regulator.

### The LelB-regulated gene *rvfA* (lpg1797) is positioned adjacent to another LTTR-encoding gene, lpg1796

**Fig 3:**
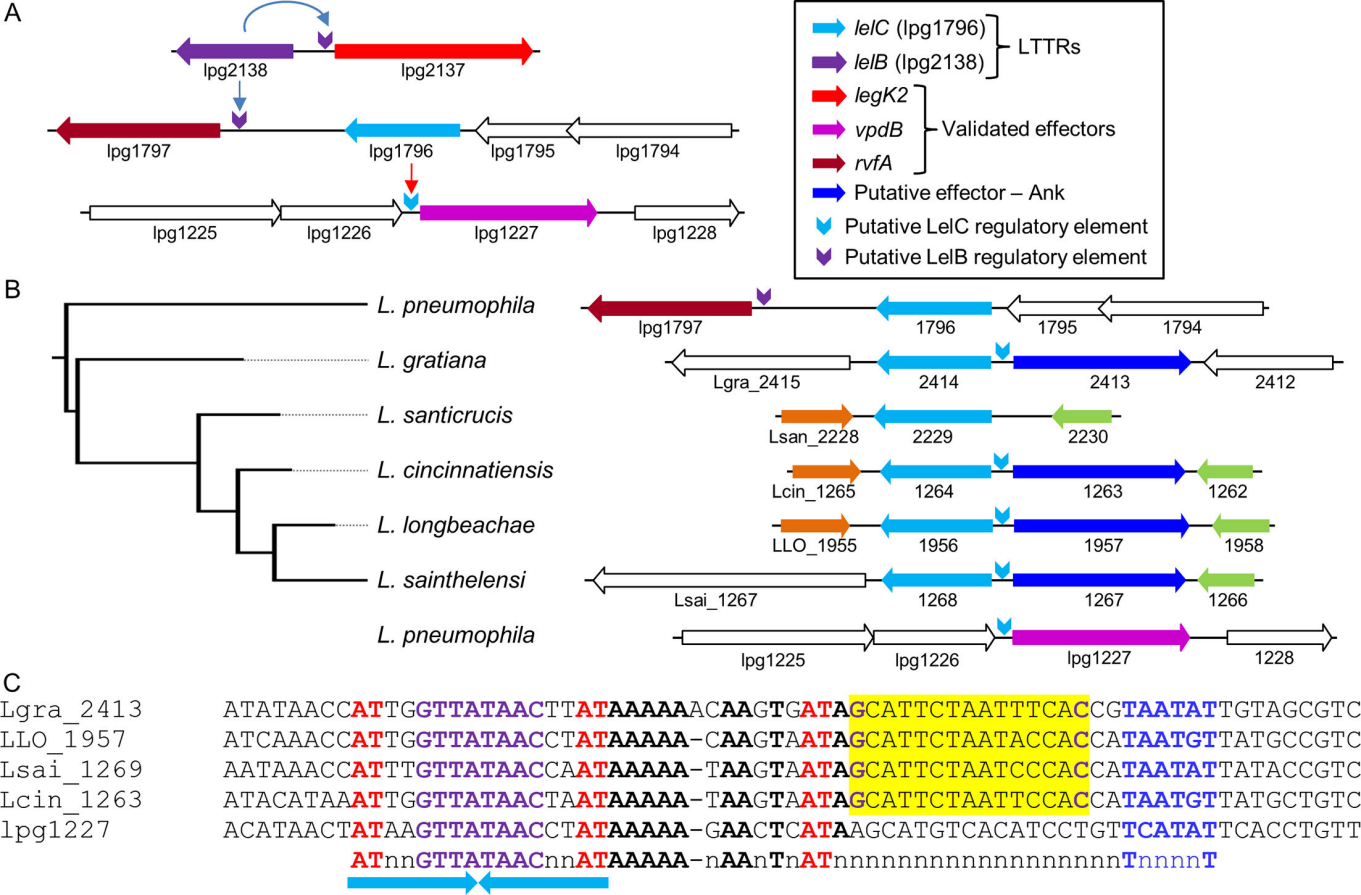
Distribution of lpg1796 (*lelC*) in the *Legionella* genus. (A) Schematic representation of the genomic regions of *lelB* and *lelC* and the EEGs they regulate. The genes are indicated by their locus tag number. The positions of the putative regulatory elements recognized by the LelB and LelC LTTRs are marked by purple and cyan arrows pointing downward, respectively; unrelated genes are marked in white. (B) Schematic representation of the genes located in the genomic region adjacent to the lpg1796 (*lelC*) LTTR orthologs in six *Legionella* species. Homologous genes are marked by the same color, and non-homologous genes are marked in white. The genes are indicated by their locus tag number. The position of the conserved regulatory elements predicted to be recognized by the LelB and LelC LTTRs is marked by purple and cyan arrows pointing downward, respectively. The maximum-likelihood phylogeny tree on the left was reconstructed using the amino acid alignment of the lpg1796 orthologous ORFs from the six *Legionella* species harboring the regulator. The lpg1227 (*vpdB*) genomic region is included even though it does not contain a *lelC* orthologous gene, to show the position of the putative regulatory element. (C). The regulatory regions of genes are located adjacent to lpg1796 and harbor a conserved regulatory element and the regulatory region of lpg1227 (*vpdB*). The putative −10 promoter element is in blue, the nucleotides representing the common LTTR motif are in red, the putative Fis regulatory elements are shaded in yellow, and their conserved nucleotides are in purple; other conserved nucleotides are in bold, and the inverted repeat sequence is marked by arrows.

As presented above, *rvfA* (lpg1797) was strongly activated by LelB (Fig. 2F and I). Examination of the *rvfA* genomic region revealed that another LTTR encoding gene (lpg1796) is located adjacent to it (Fig. 3A). Bioinformatic analysis indicated that lpg1796 close orthologs (with at least 74% identity, 82% similarity in amino acids, and a BLAST *e* value of ≥5e-^174^) are found in six *Legionella* species, and in four of them, the lpg1796 orthologs are positioned adjacent to the same gene encoding a putative effector (not present in *L. pneumophila*), which harbor an ankyrin repeat, usually found in *Legionella* effector proteins (14, 16) (Fig. 3B). Examination of the intragenic region between the genes encoding the lpg1796 orthologs and the orthologs of the putative EEG revealed a conserved regulatory element somewhat similar to LTTR consensus sequences (Fig. 3C). Interestingly, an identical putative regulatory element was also identified in the upstream regulatory region of a single *L. pneumophila* EEG–*vpdB* (lpg1227) (58) (Fig. 3C), which is not located adjacent to lpg1796 (Fig. 3A). Following these bioinformatic results and the experimental results presented hereafter, lpg1796 was named LelC for *Legionella* EEGs (effector-encoding genes) LTTR (LysR-type transcription regulator).

### LelC regulates the expression of the *L. pneumophila vpdB* EEG

**Fig 4:**
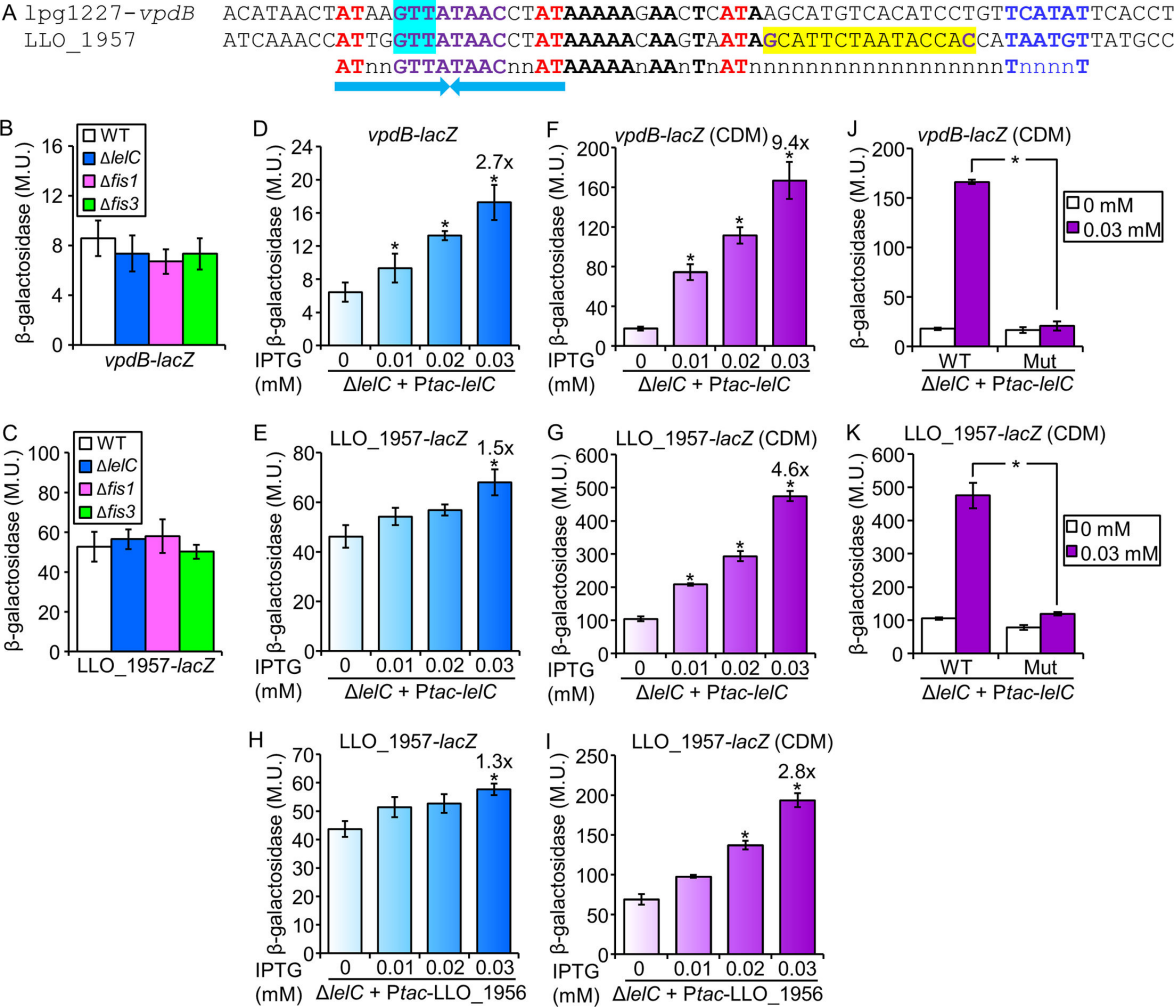
LelC activates the expression of *L. pneumophila* and *L. longbeachae* EEGs. (A) The regulatory regions of *L. pneumophila* EEG (*vpdB*) and *L. longbeachae* putative EEG (LLO_1957) harbor the LelC conserved regulatory element. The putative −10 promoter elements are in blue, the nucleotides representing the common LTTR motif are in red, the putative Fis regulatory elements are shaded in yellow and their conserved nucleotides are in purple, the nucleotides shaded in cyan were mutated (GTT to CAA), other conserved nucleotides are in bold, and the inverted-repeat sequence is marked by arrows. (B, C) The expression of *vpdB* (**B**) and LLO_1957 (**C**) *lacZ* fusions was examined in wild-type (WT) *L. pneumophila* and the *lelC*, *fis1*, and *fis3* deletion mutants. (D–G) The expression of *vpdB* (**D, F**) and LLO_1957 (**E, G**) *lacZ* fusions was examined in *L. pneumophila* containing a deletion in *lelC* in AYE medium (**D, E**) or CDM (**F, G**). The bacteria examined contained a plasmid with the *L. pneumophila lelC* gene cloned under the control of the P*_tac_* promoter (activated by IPTG), and they were grown in medium containing different concentrations of IPTG (indicated below the bars). The levels of expression of the *lacZ* fusions were found to be significantly different (*P < 10^−4^, unpaired Student’s *t*-test) between expression levels of the same *lacZ* fusions examined without IPTG and with different IPTG concentrations. (H, I). A similar analysis to the one presented in panels (E–G) was also performed using the *L. longbeachae* LelC ortholog (LLO_1956). (J, K) LelC requires its putative regulatory element to activate the expression of its target genes. The expression of wild-type *lacZ* fusions (WT) of *vpdB* (**H**) and LLO_1957 (**I**) and the same fusions containing a mutation (Mut) in the suspected LelC regulatory element was examined in *L. pneumophila* containing a deletion in *lelC*. The bacteria examined contained a plasmid with the *L. pneumophila lelC* gene cloned under the control of the P*_tac_* promoter (activated by IPTG), and they were grown in CDM with (1 mM) and without IPTG. The levels of expression of the wild-type and mutated *lacZ* fusions were found to be significantly different (*P < 10^−4^, unpaired Student’s *t*-test) between the fusions examined with IPTG. β-Galactosidase activity was measured as described in the Materials and Methods. Data (expressed in Miller units [M.U.]) represent the average ± standard deviations (error bars) from at least three different biological replicates.

Since the putative LelC regulatory element was identified only in the regulatory region of a single *L. pneumophila* EEG (*vpdB*), we also included in our analysis the *Legionella longbeachae* LLO_1957 encoding a putative effector which is located adjacent to the *L. longbeachae* lpg1796 ortholog (LLO_1956) (Fig. 3B and 4A). To determine whether LelC regulates the expression levels of *vpdB* and LLO_1957, we constructed a deletion mutant of the *lelC* gene. Examining this deletion mutant in *A. castellanii* indicated that the *lelC* gene is dispensable for intracellular growth in this host cell (Fig. S1), and similar results were also reported in other host cells (57). Examination of the *vpdB* and LLO_1957 expression levels in *L. pneumophila lelC* deletion mutant as well as in the two *L. pneumophila fis* deletion mutants resulted in no effect on their expression (Fig. 4B and C). Next, we examined whether the *L. pneumophila* LelC overexpression affects the expression levels of *vpdB* and LLO_1957, and both genes showed weak (but significant) levels of activation (Fig. 4D and E). Examination of the LelC overexpression constructs in the background of *fis1* and *fis3* deletion mutants resulted in better activation only for LLO_1957 (compare Fig. 4D and E with Fig. S3A and B), which harbors a putative Fis regulatory element (Fig. 4A). These results, and the knowledge that LTTRs harbor a sensory domain that affects their activity, led us to examine the activation by LelC in different growth conditions. To this end, the bacteria were grown in a chemically defined medium (CDM), which was previously shown to support *Legionella* growth (59, 60). Examination of the LelC overexpression constructs in CDM led to stronger activation by the *L. pneumophila* LelC of both *vpdB* and LLO_1957 in comparison to the activation obtained using the standard *Legionella* non-defined growth medium AYE (compare Fig. 4D, E, F through G, respectively). In addition, we also examined the effect of the overexpression of the *L. longbeachae* LelC ortholog (LLO_1956) on the expression of LLO_1957 in both AYE and CDM and obtained similar results to those with the *L. pneumophila* LelC ortholog (Fig. 4H and I). Collectively, we concluded that the LelC regulator activates the expression level of the *L. pneumophila* EEG *vpdB* and the *L. longbeachae* putative EEG (LLO_1957).

### LelB and LelC require the conserved regulatory element located upstream of their target EEGs to activate their expression

To examine the connection between the LelB and LelC regulators and the regulatory elements identified in their putative target genes, we constructed mutations in the consensus sequences identified in each of the five genes examined (*legK2*, *rvfA*, and *lem4* for LelB; Fig. 2A and *vpdB* and LLO_1957 for LelC; Fig. 4A). The *lacZ* fusions harboring these mutations were cloned into the inducible expression system described above and examined with *lelB* (*legK2*, *rvfA*, and *lem4*) in the *fis1* deletion mutant, and with *lelC* (*vpdB* and LLO_1957) in the *lelC* deletion mutants in CDM. The results obtained indicated that the mutations in the regulatory elements identified completely abrogated the ability of LelB and LelC to activate the expression of the corresponding EEGs (Fig. 2K through M, 4J and K). To further determine the specificity of activation by LelB and LelC, we examined the reciprocal effect of LelB and LelC overexpression on each other’s target EEGs, as well as on two other EEGs (*lubX* and *legK3*) known to be regulated by Fis and RegK3 (38, 40), respectively. All these analyses were performed in the *fis1* deletion mutant for LelB and in CDM for LelC (in which the highest degree of activation was observed for each regulator). No activation was obtained in these assays (Fig. S2B and S3C), supporting the specificity of LelB and LelC to the activation of the target genes we originally identified. Collectively, our results further indicate that the conserved regulatory elements we identified play a critical role in the activation of *legK2*, *rvfA*, and *lem4* by LelB and the activation of *vpdB* and LLO_1957 by LelC.

### LelB and LelC directly bind to the regulatory region of the effectors they regulate

**Fig 5:**
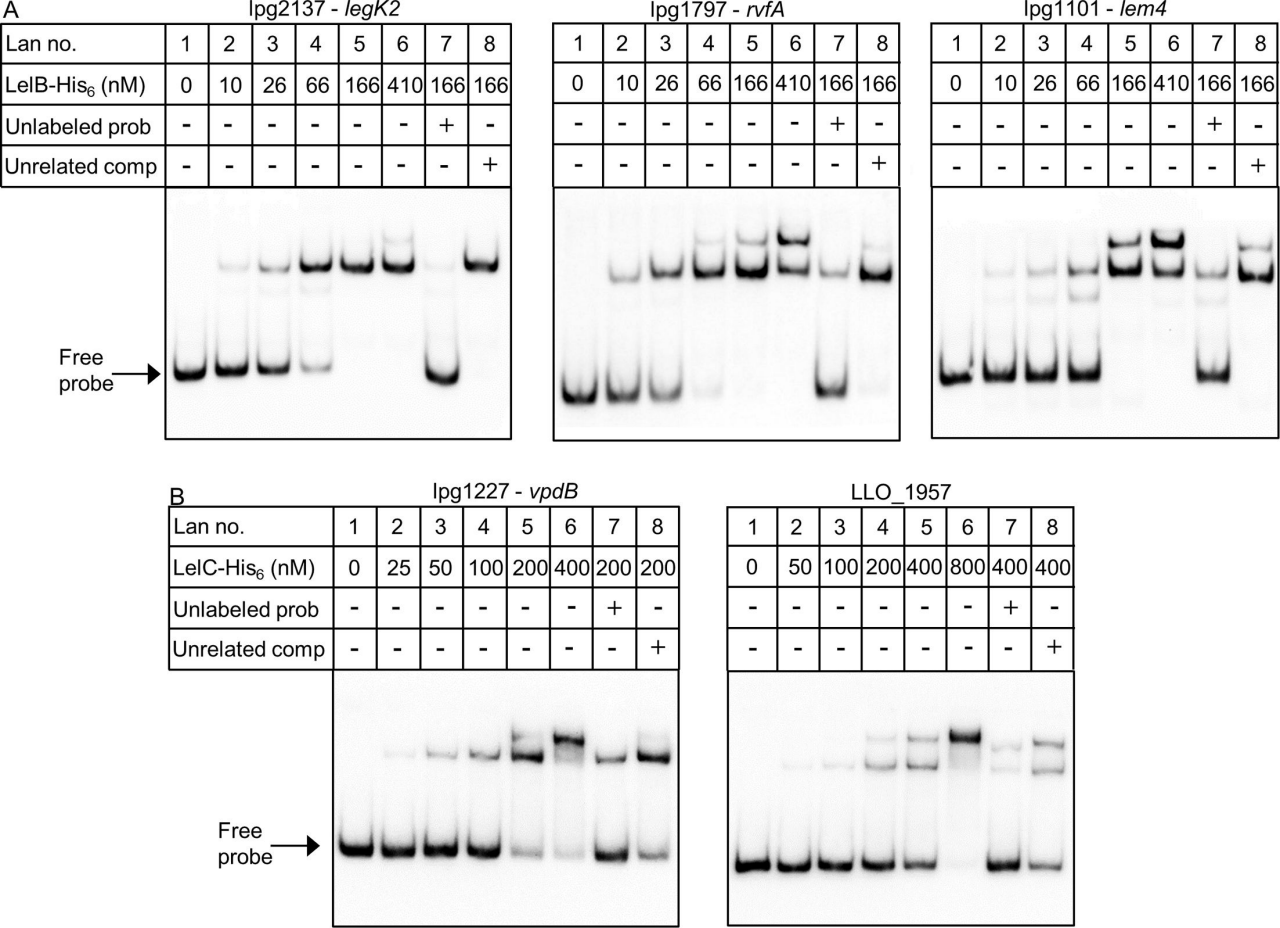
The *L. pneumophila* LelB-His_6_ and LelC-His_6_ proteins bind to the regulatory regions of their target genes. Gel mobility shift assays were performed with purified LelB-His_6_ protein (**A**) and the DIG-labeled *legK2*, *rvfA*, and *lem4* regulatory regions, and with purified LelC-His_6_ protein (**B**) and the DIG-labeled *vpdB* and LLO_1957 regulatory regions. The first lane did not contain any protein. The rest of the lanes contained increasing amounts of the LelB-His_6_ (**A**) or LelC-His_6_ (**B**) proteins. The competition assays (lanes 7 and 8 in each panel) were performed using an unlabeled probe as a specific competitor (unlabeled probe) or the same amount of the regulatory region of another EEG (*regK3*) (Unrelated comp).

To further test our results, the *L. pneumophila* LelB and LelC proteins were His-tagged, overexpressed, purified (Fig. S4), and used for gel mobility shift assays with the regulatory regions of *legK2*, *rvfA*, and *lem4* for LelB and *vpdB* and LLO_1957 for LelC. The *L. pneumophila* LelB-His_6_ and LelC-His_6_ proteins bound to the regulatory region of the corresponding genes, as evident by a migration shift of the DNA probe (Fig. 5). The amounts of shifted probes positively correlated with the amounts of LelB-His_6_ or LelC-His_6_ proteins (Fig. 5A and B, lanes 2–6). In addition, competition with an unlabeled probe reduced the band shift (Fig. 5A and B, compare lanes 5 and 7). To further test the binding specificity, we performed additional competition assays with an unlabeled probe containing the regulatory region of another EEG (*legK3*), which showed no reduction in the band shift (Fig. 5A and B, compare lanes 7 and 8). Collectively, the mobility shift assays, together with the LelB and LelC overexpression analysis, and the analyses of the mutations in the LelB and LelC consensus sequences (Fig. 2 and 4), establish that LelB is a direct activator of *legK2*, *rvfA,* and *lem4* and that LelC is a direct regulator of *vpdB* in *L. pneumophila*.

### LelB and LelC expression is regulated by the RpoS sigma factor

**Fig 6:**
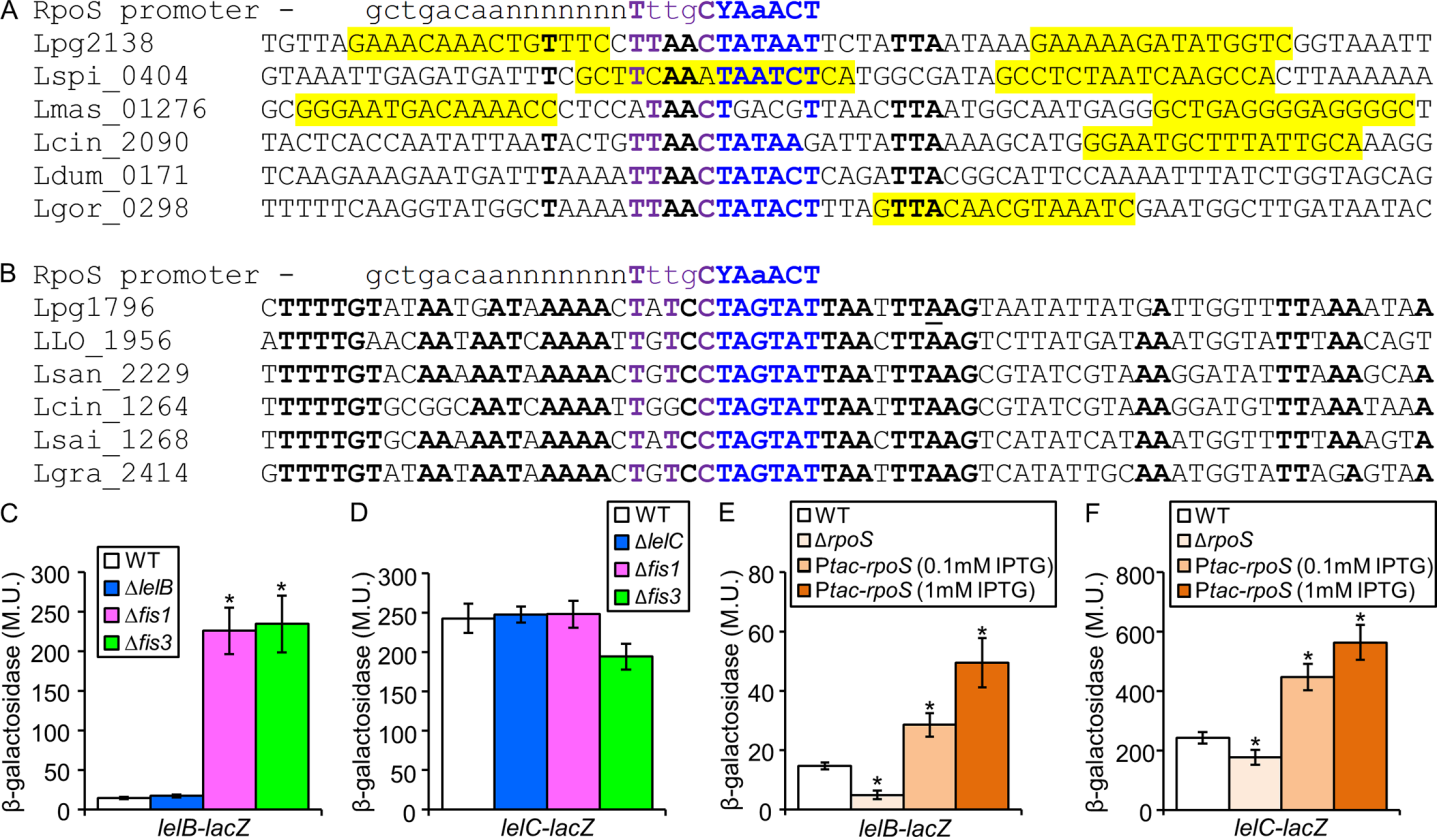
The expression of the *lelB* and *lelC* genes is activated by RpoS. (A, B) The regulatory regions of the *lelB* (**A**) and *lelC* (**B**) orthologous genes. The −10 promoter elements are in blue, the RpoS extended −10 promoter is in purple, the putative Fis regulatory elements are shaded in yellow, relatively conserved nucleotides are marked in bold, the experimentally validated transcription start site of the *L. pneumophila lelC* gene is in bold and underlined, and the RpoS consensus sequence is indicated. (C, D) Expression of *lelB* (**C**) and *lelC* (**D**) *lacZ* fusions was examined in wild-type *L. pneumophila*, the *lelB* or *lelC* deletion mutants, and the *fis1* and *fis3* deletion mutants. The levels of expression of the *lelB lacZ* fusion were found to be significantly different (*P < 10^−4^, unpaired Student’s *t*-test) between expression levels in the *fis1* and *fis3* deletion mutants and those in the wild-type strain. (E, F) The expression of the *lelB* (**E**) and *lelC* (**F**) *lacZ* fusions in wild-type *L. pneumophila*, the *rpoS* deletion mutants, and the *rpoS* deletion mutant with *rpoS* cloned under P*_tac_* control was examined. The levels of expression of the *lelB* and *lelC lacZ* fusions were found to be significantly different (*P < 10^−4^, unpaired Student’s *t*-test) between the expression in the wild-type strain and the *rpoS* deletion mutant as well as with RpoS complementation. β-Galactosidase activity was measured as described in the Materials and Methods. Data (expressed in Miller units [M.U.]) represent the average ±standard deviations (error bars) from at least three different biological replicates.

We were interested in exploring how the *lelB* and *lelC* genes themselves are regulated. Examination of the regulatory region of the *lelB* and *lelC* orthologs from different *Legionella* species indicated the existence of putative Fis regulatory elements in the *lelB* regulatory region and that the putative extended −10 promoter sequence of both *lelB* and *lelC* closely matches that of the consensus sequence of the *E. coli* RpoS (61, 62), and the *L. pneumophila lelA* RpoS promoter (41) (lpg2138 (*lelB*) and lpg1796 (*lelC*), Fig. 6A and B, respectively). Examination of the level of expression of the *lelB* and *lelC* genes indicated that the *lelB* gene was expressed at very low levels and was strongly upregulated in the *fis1* and *fis3* deletion mutants (Fig. 6C). The *lelC* gene was expressed at much higher levels and was not affected by the *fis* deletions (Fig. 6D). To examine whether the RpoS sigma factor regulates the expression levels of *lelB* and *lelC*, their expression levels were examined in *rpoS* deletion mutants, and, indeed, a significant reduction in their expression levels was observed (Fig. 6E and F). These results fit the previously published microarray analysis, comparing wild-type *L. pneumophila* to an *rpoS* deletion mutant (46). Furthermore, overexpression of RpoS increased the expression level of both *lelB* and *lelC* (Fig. 6E and F), indicating that RpoS positively regulates the expression of both regulators. Our results revealed that the three LTTRs (LelA (41), LelB, and LelC) controlling the expression of EEGs in *L. pneumophila* are connected to the EEG regulatory network through RpoS and Fis.

### Distribution of the LelB orthologs and putative target genes in the *Legionella* genus

The validation of the conserved target regulatory elements recognized by LelB and LelC (Fig. 2K through M, 4J and K) made it possible to search for these regulatory elements in the genomic sequences of all the *Legionella* species harboring them. This analysis revealed that the regulatory element recognized by LelC is found solely in the regulatory region of the genes located adjacent to the regulator and in the *L. pneumophila vpdB* upstream regulatory region (Fig. 3C). However, the search for LelB target regulatory elements showed differences between clade I, II, and clade III (Fig. 1A). In the *Legionella* species harboring the LelB orthologs belonging to clades I and II, the following situations were found: in five *Legionella* species (*L. spiritensis*, *L. cincinnatiensis*, *L. dumoffii,* and in *L. gormanii* with both paralogs), the LelB target regulatory element was identified only in the gene located adjacent to the regulator-encoding gene, in three *Legionella* species (*L. pneumophila*, *L. massiliensis,* and *L. qingyii*) beside in the gene located adjacent to the regulator, several (two, seven, and a single gene, respectively) additional genes located distantly from the regulator were found to contain the LelB regulatory element, and in a single *Legionella* species (*L. saoudiensis*), a single gene located distantly from the regulator was found to contain the LelB regulatory element (see Fig. 1; Fig. S5, S6 and Data set S1). On the contrary, the search for the LelB target regulatory element in the five species harboring the clade III LelB orthologs led to an intriguing result. In these five *Legionella* species, between 19 and 36 genes positioned throughout their genomes were found to contain a putative LelB regulatory element (Fig. S5, S7A through E; Data set S2) (see below for further details about these proteins).

To identify additional differences between the LelB clades (besides the difference in the number of predicted target genes), we investigated whether the genes encoding these orthologs undergo HGT. To do this, we compared the LelB protein tree with the *Legionella* species phylogenetic tree (Fig. S8). This analysis revealed that clade III LelB orthologs closely align with the species phylogeny, suggesting vertical inheritance. By contrast, clade I and clade II LelB orthologs exhibit notable discrepancies, which may indicate HGT events (Fig. S8). Further supporting this hypothesis, in several clade I and clade II *lelB* orthologs, we identified genomic islands containing both the *lelB* ortholog and its predicted target gene (Fig. S9). By contrast, no such genomic islands were detected for the clade III *lelB* orthologs.

Collectively, these findings suggest that clade I and II LelB orthologs appear to function as local regulators that undergo HGT within the *Legionella* genus and regulate the expression of adjacently located genes and a few distantly located genes, whereas clade III LelB orthologs appear to function as global regulators that remain fixed at the same genomic locus and regulate the expression of numerous putative EEGs located throughout the genome.

### Examination of pairs of LelB paralogs for cross-regulation

**Fig 7:**
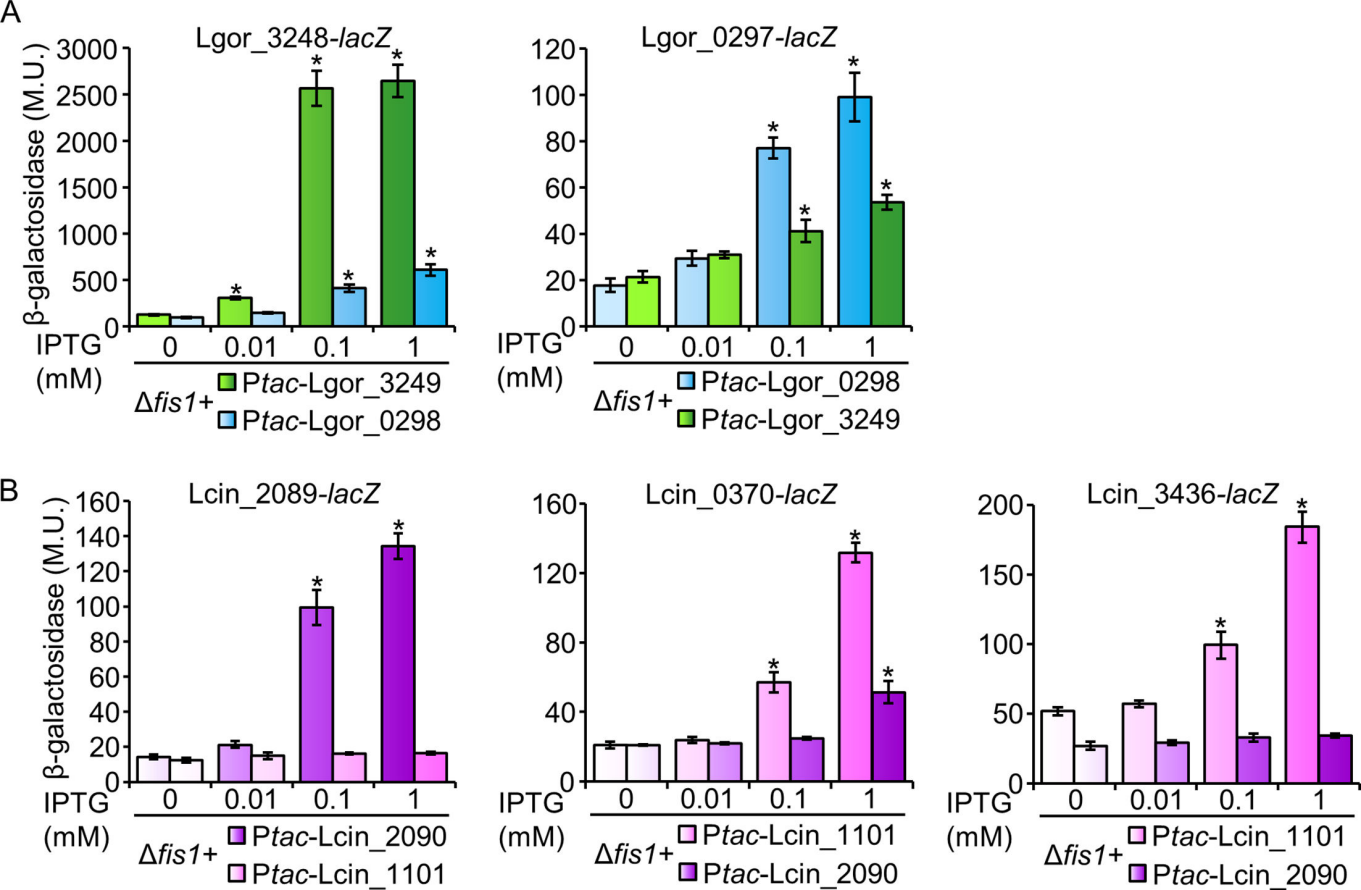
Cross-regulation occurs between *L. gormanii* LelB paralogs but not between *L. cincinnatiensis* LelB paralogs. Bacterial strains carrying a plasmid with a *lacZ* fusion were examined, where each fusion was cloned together with *L. gormanii* (**A**) or *L. cincinnatiensis* (**B**) *lelB* paralogs. The *lelB* paralogs were expressed under the P*_tac_* promoter, activated by IPTG, in the *L. pneumophila fis1* deletion mutant. Strains were tested in medium without or with IPTG (0.01 mM, 0.1 mM, and 1 mM). The examined genes are indicated above the graphs. The levels of expression of the *lacZ* fusions were found to be significantly different (*P < 10^−4^, unpaired Student’s *t*-test) between the strains without IPTG and those with IPTG. β-Galactosidase activity was measured as described in the Materials and Methods. Data (expressed in Miller units [M.U.]) represent the average ± standard deviations (error bars) from at least three different biological replicates.

In three *Legionella* species (*L. gormanii*, *L. massiliensis*, and *L. cincinnatiensis*), two LelB paralogs were identified. In two of the species (*L. massiliensis* and *L. cincinnatiensis*), the paralogs belong to different LelB clades (clade I and II in *L. massiliensis* and clade I and III in *L. cincinnatiensis*), while in *L. gormanii,* both paralogs belong to clade II (Fig. S8). We wondered whether the LelB paralogs located adjacent to their putative target gene exclusively regulate the adjacent gene or if cross-regulation occurs between them. To this end, we examined the regulation mediated by the two *L. gormanii* paralogs. We constructed an overexpression system for the Lgor_0298 LTTR and its putative target gene (Lgor_0297), as well as for the Lgor_3249 LTTR and its putative target gene (Lgor_3248), including reciprocal constructs to test cross-regulation. Both the natural and reciprocal pairs were cloned in an identical configuration, with the target gene positioned adjacent to the regulator. The results obtained showed that both natural regulator-target pairs activated their adjacent target gene (Fig. 7A). However, in the reciprocal analysis, activation was still observed but was weaker than that of the natural target gene (Fig. 7A). These results suggest that the natural regulator-target pairs likely co-evolve to fit one another, but due to the high degree of homology between the regulators and between their regulatory elements, cross-regulation may occur.

The pair of LelB paralogs in *L. cincinnatiensis* represents the only case in which paralogs from clade I (or clade II) and clade III are found in the same species. Since paralogs from these two clades differ in several parameters, including the number of their suspected target genes, the position of their suspected target genes, and their genomic organization (see above), we decided to also examine the *L. cincinnatiensis* paralogs. We constructed a similar system to the one described above for the *L. gormanii* LelB paralogs. The Lcin_2090 LTTR and its putative target gene (Lcin_2089) were examined, as well as the Lcin_1101 LTTR and two putative target genes that are not located adjacent to either of the two *lelB* paralogs. These two putative EEGs (Lcin_0370 and Lcin_3436) are present in three or four of the *Legionella* species harboring a LelB ortholog from clade III, and they also harbor the putative LelB regulatory element (Data set S2); thus, they are suspected to be part of the global regulon of LelB. Reciprocal constructs were also constructed for both LelB paralogs to test cross-regulation. The results obtained showed that the natural regulator-target pair (Lcin_2090 LTTR and Lcin_2089), as well as Lcin_1101 LTTR, activated their respective target genes (Fig. 7B). However, in the reciprocal analysis, only a minor effect of Lcin_2090 on Lcin_0370-*lacZ* at the highest IPTG concentration was obtained (Fig. 7B). These results further support our previous findings (described above) that LelB orthologs from clade III are different from the clade I orthologs not only in their genomic position and number of target genes but also in their ability to activate each other’s target genes.

### Genes regulated by LelB orthologs from clade III predominantly encode putative effector proteins

**Fig 8:**
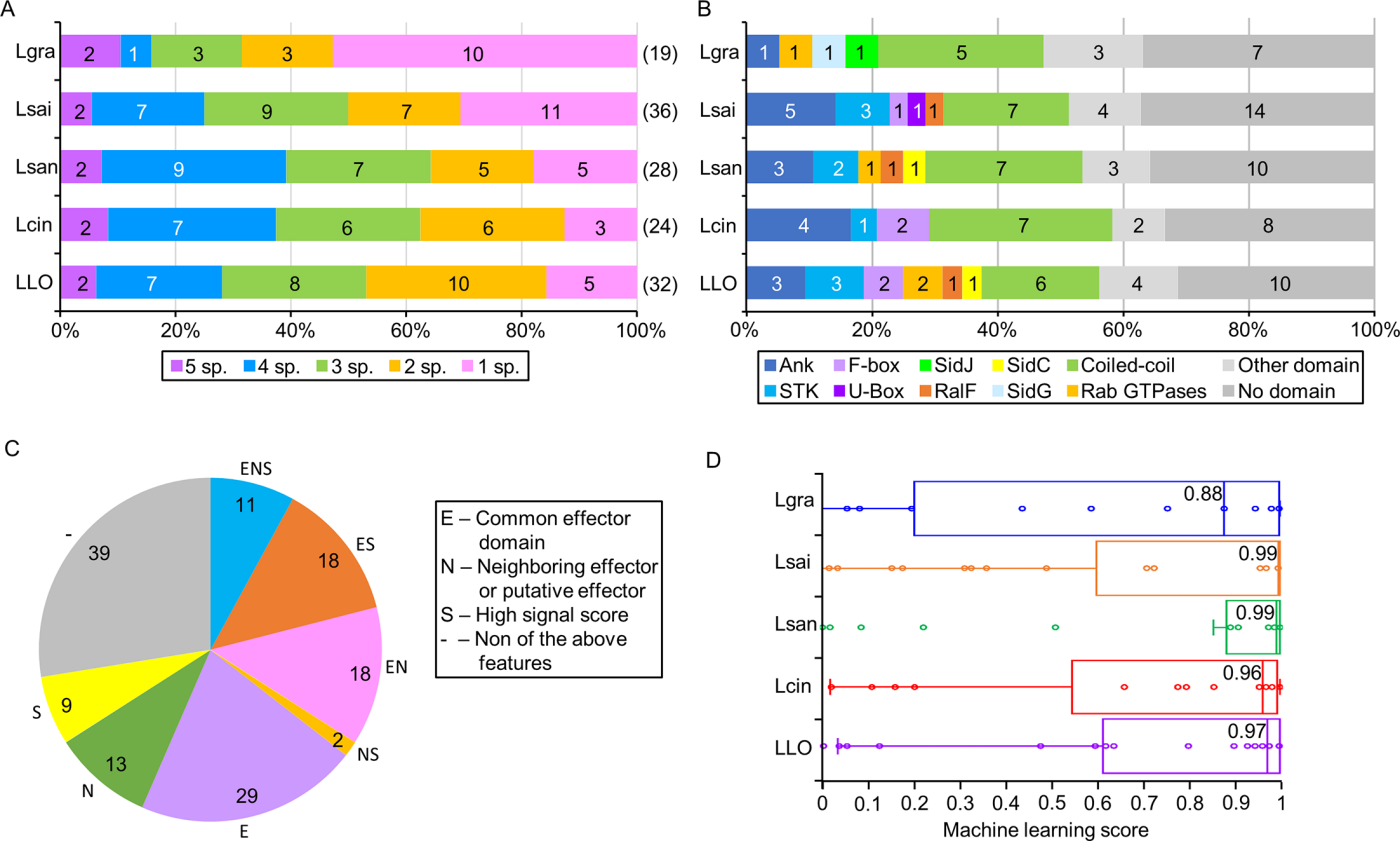
Properties of putative effectors predicted to be regulated by LelB. (A) The distribution of the LelB putative target genes between the five *Legionella* species belonging to the clade III LelB orthologs (see Fig. 1), *L. gratiana* (Lgra), *L. sainthelensi* (Lsai), *L. santicrucis* (Lsan), *L. cincinnatiensis* (Lcin), and *L. longbeachae* (LLO), was examined. The number of target genes shared between the five *Legionella* species is indicated. (B) Distribution of common *Legionella* effector domains in the proteins encoded by the LelB putative target genes, ankyrin repeats (Ank), serine/threonine kinase domain (STK), and E3 ligase domains (F-box and U-box). In addition, orthologs of the *L. pneumophila* SidJ, SidC, RalF, and SidG effectors were identified. (C) Distribution of features common to *L. pneumophila* effectors found in genes harboring the LelB regulatory element and the proteins they encode. The number of proteins harboring common *Legionella* effector domains (E), the number of genes harboring the LelB regulatory element located adjacent to other EEGs or putative EEGs (N), the number of proteins harboring a high type IV secretion signal score (according to (19)) (S), and the number of genes/proteins harboring none of the above features (-), are presented. (D) Box-and-whiskers plot of the machine learning effector prediction scores of the genes harboring the LelB regulatory element. The machine learning scores were predicted as in Reference (14). The median score for each *Legionella* species is indicated near the median line.

The surprising observation that a large number of genes harbor the LelB regulatory element in the five *Legionella* species belonging to clade III led us to examine the properties of the 139 proteins (belonging to 69 orthologous groups) encoded by the genes harboring the LelB regulatory element (Data set S2). First, we examined how many of these genes are shared between the five species. We found that only two genes are shared between all five species, and about 40% of the genes are present only in one or two of the species (Fig. 8A), indicating that mostly different genes are predicted to be regulated by LelB in these five species. One of the most interesting findings was their predicted protein domains. About half of these proteins contain eukaryotic protein domains commonly found in *Legionella* effector proteins, including ankyrin repeats, U-box and F-box ubiquitin E3 ligase domains, GEF (guanine nucleotide exchange factor) domains, serine/threonine kinase domains, to name a few (Fig. 8B). We then examined several features that are typically found in *Legionella* effectors: (i) presence of common effector domains (14, 16, 51, 52), (ii) presence of type IV C-terminal secretion signal (18, 19), and (iii) presence of neighboring EEGs or putative EEGs (14, 20). This analysis revealed that more than 70% of these genes were found to harbor at least one of these features, and 35% of them harbor at least two of these features (Fig. 8C). Furthermore, examining the previously calculated machine-learning effector prediction scores of these proteins, indicated that their median score was higher than 0.96 (out of 1) in four out of the five species, and in the fifth species, the median score was 0.88 (Fig. 8D) (14). Previous analysis of *L. pneumophila* ORFs harboring similarly high machine-learning effector prediction scores indicated that 90% of them indeed translocate into host cells (14). Collectively, these observations suggest that in these five *Legionella* species, the LelB orthologs function as global regulators, and they regulate the expression of numerous putative EEGs located throughout their genomes.

### LelB controls the expression level of *L. longbeachae* EEGs and putative EEGs harboring the LelB regulatory element

**Fig 9:**
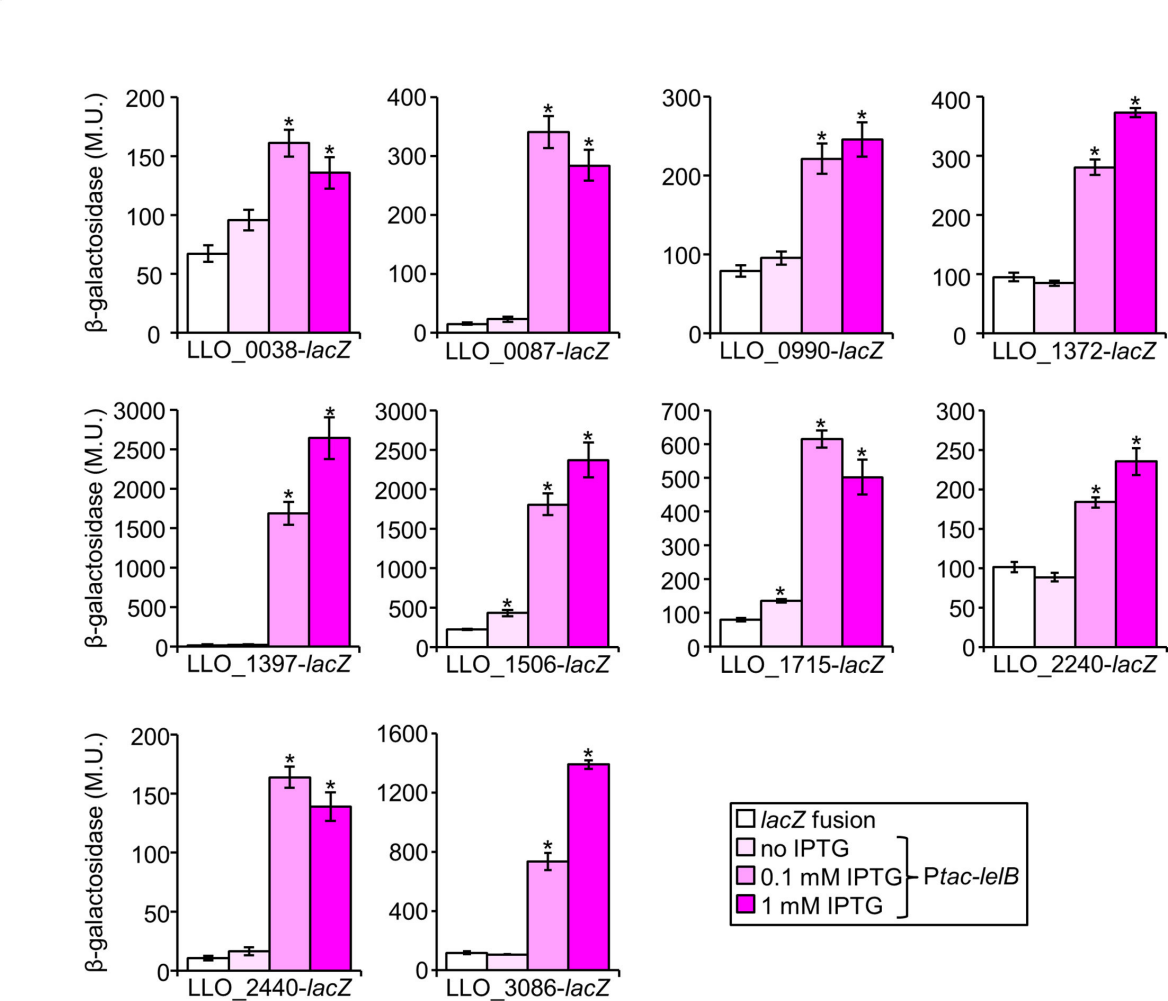
LelB activates the expression of *L. longbeachae* genes harboring the LelB putative regulatory element. The bacteria examined contain a plasmid with a *lacZ* fusion and the same *lacZ* fusion with the *lelB* gene cloned under the control of the P*_tac_* promoter (activated by IPTG) and were examined in the *fis1* deletion mutant. The strains harboring the plasmids containing the regulator were examined in medium with (0.1 mM and 1 M) or without IPTG. The *L. longbeachae* genes examined are indicated below the graphs. The levels of expression of the *lacZ* fusions were found to be significantly different (*P < 10^−4^, unpaired Student’s *t*-test) between the *lacZ* fusion without the regulator and with the regulator grown with IPTG. β-Galactosidase activity was measured as described in the Materials and Methods. Data (expressed in Miller units [M.U.]) represent the average ± standard deviations (error bars) from at least three different biological replicates.

To experimentally determine whether LelB regulates the expression of the genes described above, we chose to examine 10 *L. longbeachae* genes harboring the putative LelB regulatory element (*L. longbeachae* was chosen for this analysis since it harbors only a single LelB ortholog from clade III). These genes harbor common effector domains (Data set S2), and two of these genes (LLO_1397 - RalF and LLO_1506 - CetLl4) are validated effectors (19, 63). We constructed *lacZ* fusions for these 10 genes and examined their expression level in the *L. pneumophila fis1* deletion mutant, in which the best activation by LelB was observed (Fig. 2). The expression levels of the genes were examined without the regulator, as well as with the *L. pneumophila lelB* cloned under an IPTG-inducible promoter in the absence and presence of IPTG. The expression levels of all 10 genes examined, which harbor the LelB regulatory element, were activated by LelB (Fig. 9). These results predict that the majority of the large collection of genes we identified, which are likely to encode effector proteins, are regulated by LelB. These results further support our finding that in five *Legionella* species, LelB functions as a global regulator of EEGs, and in other species, it functions as a local regulator of EEGs, thus presenting a remarkable transition in the function of LTTRs in the regulation of EEGs in the *Legionella* genus.

## DISCUSSION

All known members of the *Legionella* genus utilize the Icm/Dot type IV secretion system to translocate proteins, termed effectors, into their diverse repertoire of host cells. The number of effectors predicted to be translocated by all members of the *Legionella* genus is enormous, estimated to be several thousand (14, 15). In *L. pneumophila* alone, about 320 effectors have been experimentally validated (18, 20, 64). These EEGs have been shown by now to be regulated at the level of gene expression by five global regulatory systems and three local regulators (see Introduction), and two novel local regulators (LelB and LelC) were described here.

**Fig 10:**
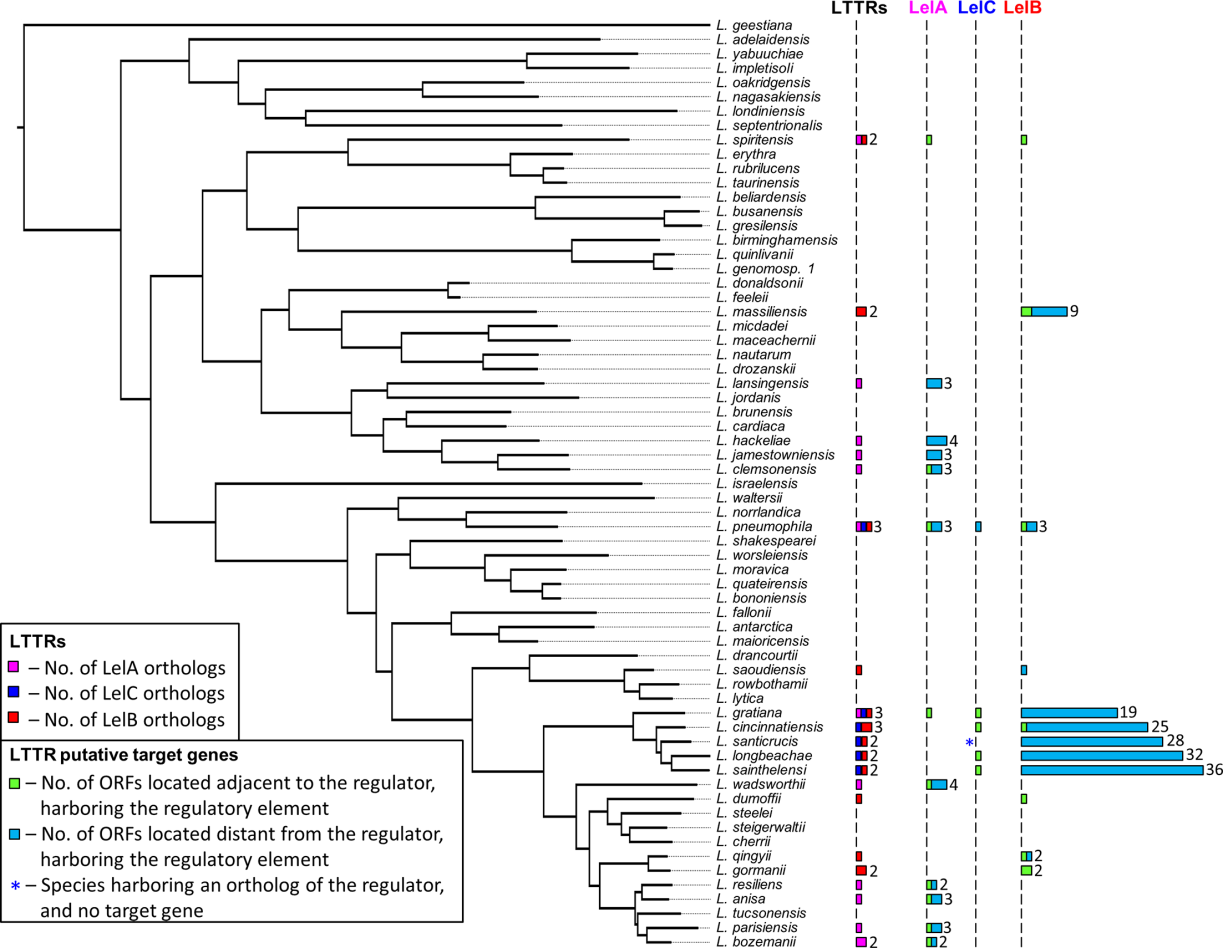
Distribution of the LelA, LelB, and LelC orthologs and their target genes in the *Legionella* genus. A maximum-likelihood tree of 65 characterized *Legionella* species was reconstructed based on the concatenated amino acid alignment of eight orthologous ORFs present in all *Legionella* species. For each species, the following are also illustrated: the number of LelA (pink), the number of LelC (blue), the number of LelB (red), the number of ORFs located adjacent to the regulator harboring the LTTR (indicated at the top) regulatory element (green), and the number of ORFs located elsewhere in the genome harboring the LTTR (indicated at the top) regulatory element (blue). Blue asterisks indicate species harboring an ortholog of the regulator and no target gene.

The two *L. pneumophila* LTTRs (LelB and LelC) described in this study, together with the previously described LTTR (LelA), were found to regulate the expression of seven EEGs. The *L. pneumophila* LelA and LelB control the expression of an EEG located adjacent to the gene encoding the regulator, as well as two other EEGs located distant in the genome ((41) and Fig. 1). The *L. pneumophila* LelC regulator controls the expression of a single EEG located distantly from the *lelC* gene; however, in the other *Legionella* species harboring a LelC ortholog, it regulates the expression of a gene encoding a putative effector located adjacent to the regulator encoding gene (Fig. 3). Expanding the view on these three LTTRs to their orthologs throughout the whole *Legionella* genus unveils the dynamics of EEGs (and putative EEGs) regulation by LTTRs (Fig. 10). The LTTR genus-wide analysis led to several interesting findings: (i) in most genes encoding orthologs of these LTTRs (22 out of 34), a target gene (in most cases an EEG or a putative EEG) harboring the regulatory element recognized by the regulator is located adjacent to the gene encoding the regulator; (ii) in 10 of these 22 orthologs, the target regulatory element recognized by the regulator was found not just upstream to the gene located adjacent to the regulator, but also upstream to additional gene(s) located distantly in the genome. Notably, some of these distant genes were experimentally validated as the regulator target genes ((41) and this study); (iii) in 11 orthologs, no gene harboring the regulatory element was found to be located adjacent to the gene encoding the regulator, and gene(s) harboring the regulatory element (in most cases EEGs or putative EEGs) were found distant in the genome; and (iv) most intriguing, in 5 of these 11 orthologs, the LelB ortholog become a global regulator, and its regulatory element was found upstream to numerous genes (Fig. 10), many of which are predicted to encode effector proteins (Fig. 8). It is important to note that the genomic synteny at the regulator position in these five closely related *Legionella* species is almost identical (Fig. 1), probably indicating a single HGT event which occurred before the speciation of these species from their last common ancestor. Interestingly, the genomic synteny at the position of the *lelA* regulator in two species (*L. jamestowniensis* and *L. hackeliae*), in which the target regulatory element was found only in genes positioned distant from the regulator encoding gene (the same three genes in both species and one additional in *L. hackeliae*), is also the same (41), probably reflecting a similar evolutionary process as described above.

Our analyses indicate that in different *Legionella* species, orthologs of the same LTTRs regulate the expression of (i) a single adjacent gene, (ii) an adjacent gene and genes located distant from the regulator, (iii) only genes located distant from the regulator, or (iv) become a global regulator which controls the expression of numerous genes scattered throughout the genome. These four settings might indicate the evolutionary process that occurs in the regulation of EEGs by LTTRs. The finding that in the majority of the LelA, LelB, and LelC, orthologs regulate a gene located adjacent to the regulator-encoding gene might suggest that the first and most frequent event that occurs is the aquation of an LTTR, its target regulatory element, and at least one target gene, by HGT. After that, such a “regulator-effector island” is established in a *Legionella* species, and the regulatory element recognized by the regulator can be acquired by additional EEGs located distantly from the regulator. Probably at a later stage, the adjacent gene is sometimes lost, and only genes located distantly are now regulated by the regulator. In a much later stage, the regulatory element recognized by the regulator can be acquired by multiple genes, resulting in the transition of an LTTR from a “regulator-effector island” into a global regulator of EEGs. Collectively, our findings suggest that the regulation of EEGs and putative EEGs by LTTRs in the *Legionella* genus is highly dynamic.

## MATERIALS AND METHODS

### Bacterial strains, plasmids, and primers

The *L. pneumophila* wild-type strain used in this study was JR32, a streptomycin-resistant, restriction-negative mutant of *L. pneumophila* Philadelphia-1, which is a wild-type strain in terms of intracellular growth (65). In addition, mutant strains derived from JR32, as well as other *Legionella* species that were used in this study, are listed in Data set S3 in the supplemental material. The *E. coli* strains used in this work are also listed in Data set S3. Plasmids and primers used in this work are listed in Data sets S4 and S5, respectively.

### Plasmid construction

To construct *lacZ* translational fusions (Data set S4), the 300 bp putative regulatory regions of the *lelB*, *lelC*, LLO_1957, LLO_0038, LLO_0087, LLO_0990, LLO_1372, LLO_1397, LLO_1454, LLO_1506, LLO_1715, LLO_1995, LLO_2329, LLO_2440, Lcin_0370, Lcin_2089, Lcin_3436, Lgor_0297, and Lgor_3248 genes were amplified by PCR using the primers listed in Data set S5. The PCR products were then digested with BamHI and EcoRI, cloned into pGS-lac-02, and sequenced, and the resulting plasmids are listed in Data set S4.

Site-directed mutagenesis was performed by regular PCR or the PCR overlap extension approach (66), as previously described (25), to construct the following substitution mutations: a substitution mutation (TAT to ATA) in the putative LelB-binding site in the regulatory region of the *legK2*, *rvfA*, and *lem4* genes; a substitution mutation (GTT to CAA) in the putative LelC-binding site in the regulatory region of the *vpdB* and LLO_1957 genes. The primers used for the site-directed mutagenesis are listed in Data set S5, and the resulting plasmids are listed in Data set S4.

To construct IPTG-inducible *lelB* (lpg2138), the *L. pneumophila lelB* gene was amplified by PCR using the primers listed in Data set S5. The PCR product was then digested with EcoRI and BamHI and cloned into pMMB207C to generate pML-pMMB207c-Ptac-lpg2138. The insert of this plasmid was sequenced and then digested with BamHI, and a Kanamycin resistance cassette (Pharmacia) was cloned into it to generate pCA-pMMB207c-Ptac-lpg2138-Km. The resulting plasmid was then digested with XbaI (for *L. pneumophila* genes) or SphI (for *L. longbeachae* genes) and NsbI, and the resulting fragment, containing P*_tac_-lelB* together with the *lacI* gene, was cloned into the plasmids containing the *lacZ* fusions of the *legK2*, *rvfA*, *lem4*, *vpdB*, *lubX*, *sidH*, LLO_0038, LLO_0087, LLO_0990, LLO_1372, LLO_1397, LLO_1454, LLO_1506, LLO_1715, LLO_1995, LLO_2329, and LLO_2440 genes digested with XmnI and XbaI or SphI, as well as plasmids containing the mutations in the LelB regulatory element in the regulatory region of *legK2*, *rvfA*, and *lem4* genes, resulting in plasmids listed in Data set S4.

To construct IPTG-inducible *lelC* (lpg1796), the *L. pneumophila lelC* gene was amplified by PCR using the primers listed in Data set S5. An internal EcoRI restriction site located inside the *lelC* coding region was eliminated by site-directed mutagenesis without changing the amino acid sequence of the protein, using the primers listed in Data set S5. The PCR product was then digested with EcoRI and BamHI and cloned into pMMB207C to generate pCA-pMMB207c-Ptac-lpg1796. The resulting plasmid was then digested with XbaI and NsbI, and the resulting fragment, containing P*_tac_-lelC* together with the *lacI* gene, was cloned into the plasmids containing the *lacZ* fusions of the *vpdB*, *rvfA*, *lubX*, *sidH*, and LLO_1957 genes digested with XmnI and XbaI, as well as plasmids containing a mutation in the LelC-binding site in the regulatory region of *vpdB* and LLO_1957, resulting in plasmids listed in Data set S4.

To construct IPTG-inducible LLO_1956, the *L. longbeachae* LLO_1956 gene was amplified by PCR using the primers listed in Data set S5. An internal EcoRI restriction site located inside the LLO_1956 coding region was eliminated by site-directed mutagenesis without changing the amino acid sequence of the resulting protein, using the primers listed in Data set S5. The PCR product was then digested with EcoRI and BamHI and cloned into pMMB207C to generate pCA-pMMB207C-P_tac_-LLO_1956. The resulting plasmid was then digested with XbaI and NsbI, and the resulting fragment, containing P*_tac_*-LLO_1956 together with the *lacI* gene, was cloned into the plasmids containing the *lacZ* fusion of the LLO_1957 gene digested with XmnI and XbaI, resulting in the plasmid listed in Data set S4.

To construct IPTG-inducible Lgor_0298 and Lgor_3249, the *L. gormanii* Lgor_0298 and Lgor_3249 genes were amplified by PCR using the primers listed in Data set S5. Two internal NsbI restriction sites located inside the Lgor_3249 coding region were eliminated by site-directed mutagenesis without changing the amino acid sequence of the resulting protein, using the primers listed in Data set S5. The PCR product was then digested with EcoRI and BamHI and cloned into pMMB207C to generate pCA-pMMB207c-Ptac-Lgor_0298 and pCA-pMMB207c-Ptac-Lgor_3249. The insert of these plasmids was sequenced and then digested with BamHI, and a Kanamycin resistance cassette (Pharmacia) was cloned into it to generate pCA-pMMB207c-Ptac-Lgor_0298-Km and pCA-pMMB207c-Ptac-Lgor_3249-Km. The resulting plasmid was then digested with SphI and NsbI, and the resulting fragments, containing P*_tac_*-Lgor_0298 or P*_tac_*-Lgor_3249 together with the *lacI* gene, were cloned into the plasmids containing the *lacZ* fusion of the Lgor_0297 and Lgor_3248 genes digested with XmnI and SphI, resulting in the four plasmids listed in Data set S4.

To construct IPTG-inducible Lcin_1101 and Lcin_2090, the *L. cincinnatiensis* Lcin_1101 and Lcin_2090 genes were amplified by PCR using the primers listed in Data set S5. Two internal EcoRI restriction sites located inside the Lcin_2090 coding region were eliminated by site-directed mutagenesis without changing the amino acid sequence of the resulting protein, using the primers listed in Data set S5. The PCR product was then digested with EcoRI and BamHI and cloned into pMMB207C to generate pCA-pMMB207c-Ptac-Lcin_1101 and pCA-pMMB207c-Ptac-Lcin_2090. The insert of these plasmids was sequenced and then digested with BamHI, and a Kanamycin resistance cassette (Pharmacia) was cloned into it to generate pCA-pMMB207c-Ptac-Lcin_1101-Km and pCA-pMMB207c-Ptac-Lcin_2090-Km. The resulting plasmid was then digested with SphI and NsbI, and the resulting fragments, containing P*_tac_*-Lcin_1101 or P*_tac_*-Lcin_2090 together with the *lacI* gene, were cloned into the plasmids containing the *lacZ* fusion of the Lcin_0370, Lcin_2089, and Lcin_3436 genes digested with XmnI and SphI, resulting in the four plasmids listed in Data set S4.

In addition, the *L. pneumophila* RpoS sigma factor (lpg1284), cloned under the P*_tac_* control in pDT-pMMB-Ptac-rpoS (41), was digested with XbaI and NsbI, and the resulting fragment, containing P*_tac_*-RpoS, together with the *lacI* gene, was cloned into the plasmids containing the *lacZ* fusion of the *lelB* and *lelC* genes digested with XmnI and XbaI, resulting in the plasmid listed in Data set S4.

To construct deletion substitution mutants in the *L. pneumophila lelB* and *lelC* genes, a 1 kb DNA fragment located on each side of the planned deletions was amplified by PCR using the primers listed in Data set S5. The resulting plasmids were digested with suitable enzymes, and the inserts were used for a four-way ligation containing the Kanamycin resistance cassette. The plasmids generated, pMG-pUC18-lpg2138-UP-Km-DW, and pNS-lpg1796-km (Data set S4), were digested with PvuII, and the resulting fragments were cloned into the pLAW344 allelic exchange vector digested with EcoRV to generate the plasmids pMG-pLAW344-UP-Km-DW-lpg2138, and pNS-lpg1796-pLAW-km (Data set S4). The allelic exchange deletion substitution mutants were constructed as previously described (67). The resulting deletion substitution mutants were examined for intracellular growth in *A. castellanii* as previously described (60).

For the construction of the plasmid expressing the His-tagged LelB and LelC, the *lelB* and *lelC* genes were amplified by PCR using the primers listed in Data set S5. An internal NdeI restriction site located inside the *lelC* coding region was eliminated by site-directed mutagenesis without changing the amino acid sequence of the protein, using the primers listed in Data set S5. The resulting fragments were cloned into pET-21a and sequenced to generate the plasmids listed in Data set S4.

### β-Galactosidase assay

β-Galactosidase assays were performed as previously described (25). *L. pneumophila* strains were grown for 48 hours on charcoal-yeast extract (CYE) plates containing chloramphenicol (Cm). The bacteria were scraped off the plate and suspended in ACES-yeast extract (AYE) broth, and the bacterial OD_600_ was calibrated to 0.1 in fresh AYE, containing different concentrations of IPTG (when indicated) and Cm. The resulting cultures were grown on a roller drum for about 18 hours, until reaching an OD_600_ of about 3.2 (early stationary phase) and used for the β-galactosidase assay. When indicated that the bacteria were examined after growth in chemically defined medium (CDM) (60), the bacteria were scraped off the CYE plate and suspended in CDM, and the bacterial OD_600_ was calibrated to 0.1 in CDM, containing different concentrations of IPTG (when indicated) and Cm. The cultures were grown on a roller drum for about 36 hours, until reaching an OD_600_ of about 2 (early stationary phase), and used for the β-galactosidase assay. The assays were done for 20, 50, or 100 µL of culture, and the substrate for β-galactosidase hydrolysis was *o*-nitrophenyl-β-D-galactopyranoside.

### Protein purification and gel mobility shift assay

LelB-His_6_ and LelC-His_6_ were purified from *E. coli* BL21(DE3) using nickel bead columns (Qiagen), according to the manufacturer’s instructions. After purification, the fractions containing the protein were dialyzed overnight against a buffer containing 10 mM Tris (pH 7.5), 50 mM KCl, 5 mM MgCl_2_, 0.1 mM EDTA, 0.1 mM dithiothreitol (DTT), and 30% Glycerol, then with the same buffer containing 50% Glycerol for a few hours, and the purified protein was then stored at −20℃. A gel mobility shift assay was performed as previously described (38), with a few modifications. The putative regulatory region of *legK2*, *rvfA*, *lem4*, *vpdB*, and LLO_1957 (151 bp, 150 bp, 150 bp, 152 bp, and 154 bp, respectively) was amplified by PCR using the primers listed in Data set S5 and 3ʹ end-labeled with digoxigenin (DIG) using DIG-11-ddUTP (Roche). For LelB, increasing amounts of the purified LelB-His_6_ protein (between 10 and 410 nM) were mixed with 2 nM of the DIG-labeled probe in buffer containing 10 mM Tris (pH 7.5), 50 mM KCl, 5 mM MgCl_2_, 0.1 mM EDTA, 0.1 mM DTT, 60 µg/mL poly[d(I-C)], 60 µg/mL bovine serum albumin (BSA), and 30 µg/mL herring sperm DNA. For LelC, increasing amounts of the purified LelC-His_6_ protein (between 25 and 400 nM) were mixed with 1 nM of the DIG-labeled probe in buffer containing 20 mM Hepes (pH 7.6), 30 mM KCl, 10 mM (NH4)_2_S0_4_, 0.2% (wt/vol) Tween 20, 1 mM EDTA, 1 mM DTT, 25 µg/mL poly[d(I-C)], 25 µg/mL poly[d(A-T)], 250 µg/mL bovine serum albumin, and 25 µg/mL herring sperm DNA. For the competition experiments, a 100-fold (for LelB) or 200-fold (for LelC) excess of the unlabeled probe or the unrelated *legK3* regulatory region (156 bp) was allowed to bind the LelB-His_6_ or LelC-His_6_ proteins for 15 min before the addition of the DIG-labeled probe. The binding reaction was carried out for 30 min at room temperature, and the samples were then loaded onto a 5% polyacrylamide–0.25 × Tris-acetic acid–EDTA (TAE) gel in 0.5 × TAE running buffer. Following electrophoresis, the gel was transferred to a nylon membrane and fixed by UV cross-linking. The DIG-labeled DNA fragments were detected using the Amersham ImageQuant-800 imaging system according to the manufacturer’s instructions.

### Reconstruction of phylogenetic trees

Trees were reconstructed based on alignments produced using MAFFT (68) of the proteins indicated for each tree. The trees were reconstructed using RAxML (69) under the LG + GAMMA evolutionary model with 100 bootstrap resampling.

## Data Availability

All relevant data are within the manuscript and its supplemental files.

## SUPPLEMENTAL MATERIAL

Supplemental figures mbio.01268-25-s0001.pdf Fig. S1 to S9.

Supplemental data sets mbio.01268-25-s0002.pdf Data sets S1 to S5.

ASM does not own the copyrights to Supplemental Material that may be linked to, or accessed through, an article. The authors have granted ASM a non-exclusive, world-wide license to publish the Supplemental Material files. Please contact the corresponding author directly for reuse.
