# Supplementary material for: Versatile regulation of effectors by novel orthologous regulators in the Legionella genus
Source: mBio. 2025 May 30;16(7):e01268-25. doi: 10.1128/mbio.01268-25 (PMC12239595; doi:10.1128/mbio.01268-25)
Supplement: Supplemental figures — Fig. S1 to S9. [file mbio.01268-25-s0001.pdf]

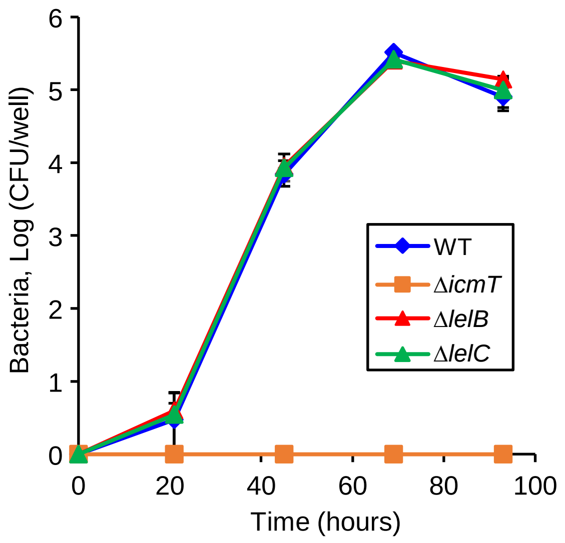

Figure S1. The *lelB* and *lelC* genes are dispensable for *L. pneumophila* intracellular growth in *A. castellanii*. Intracellular growth assay comparing *L. pneumophila* JR32 wild-type strain (WT, blue diamond), a *lelB* deletion mutant (Δ*lelB*, red triangle), a *lelC* deletion mutant (Δ*lelC*, green triangle), and an *icmT* deletion mutant (Δ*icmT*, orange square) in *A. castellanii*. CFU – colony-forming units.

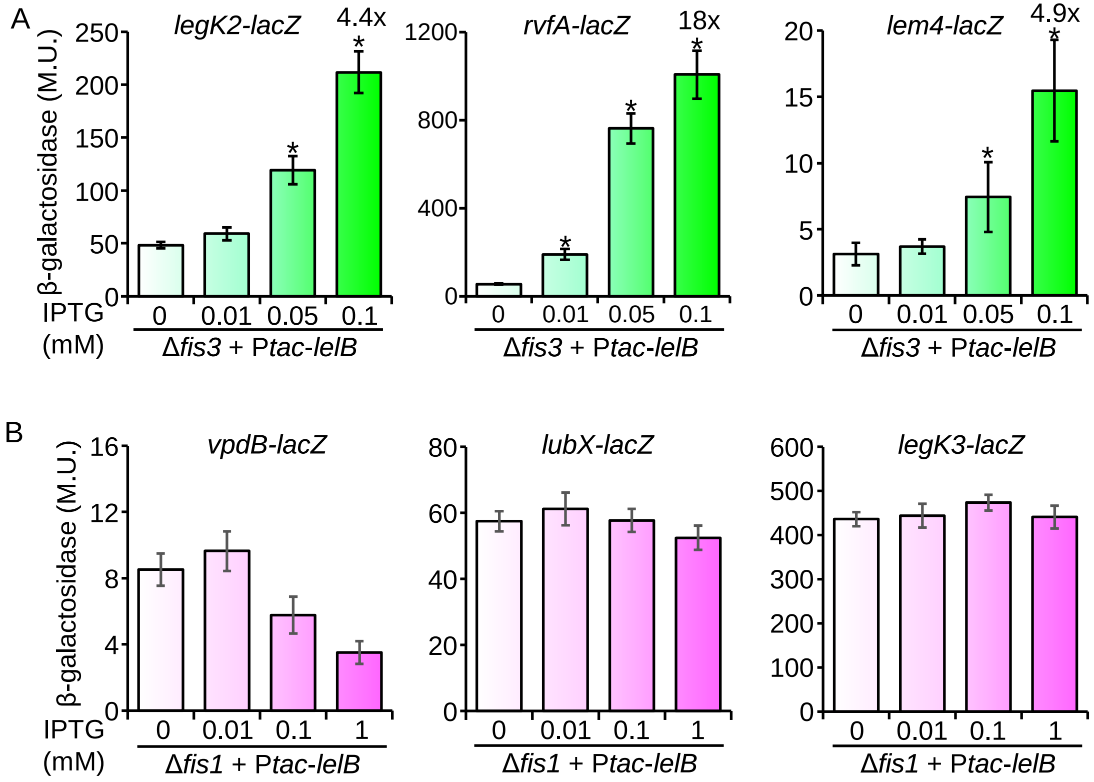

Figure S2. The effect of LelB on the expression of its target EEGs and control EEGs. A. The expression levels of *legK2*, *rvfA*, and *lem4 lacZ* fusions were examined in *L. pneumophila fis3* deletion mutant. The bacteria examined contained a plasmid with the *L. pneumophila lelB* gene cloned under the control of the P*tac* promoter (activated by IPTG), and they were grown in media containing different concentrations of IPTG (indicated below the bars). The expression in the *fis3* deletion mutant was not examined using 1 mM IPTG, since this concentration inhibited bacterial growth, probably due to the high expression of the EEGs regulated by LelB from the genome. The expression levels of the *lacZ* fusions were found to be significantly different (**p* $< 10^{-4}$, unpaired Student's *t*-test) between expression levels of the same *lacZ* fusions examined without IPTG and with different IPTG concentrations. B. The expression of *lacZ* fusions of *vpdB*, *lubX*, and *legK3*, were examined in *L. pneumophila fis1* deletion mutant. The bacteria examined contained a plasmid with the *L. pneumophila lelB* gene cloned under the control of the P*tac* promoter (activated by IPTG), and they were grown in media containing different concentrations of IPTG (indicated below the bars). β-Galactosidase activity was measured as described in the Materials and Methods section. Data (expressed in Miller units [M.U.]) are the average ± standard deviations (error bars) of the results from at least three different biological replicates.

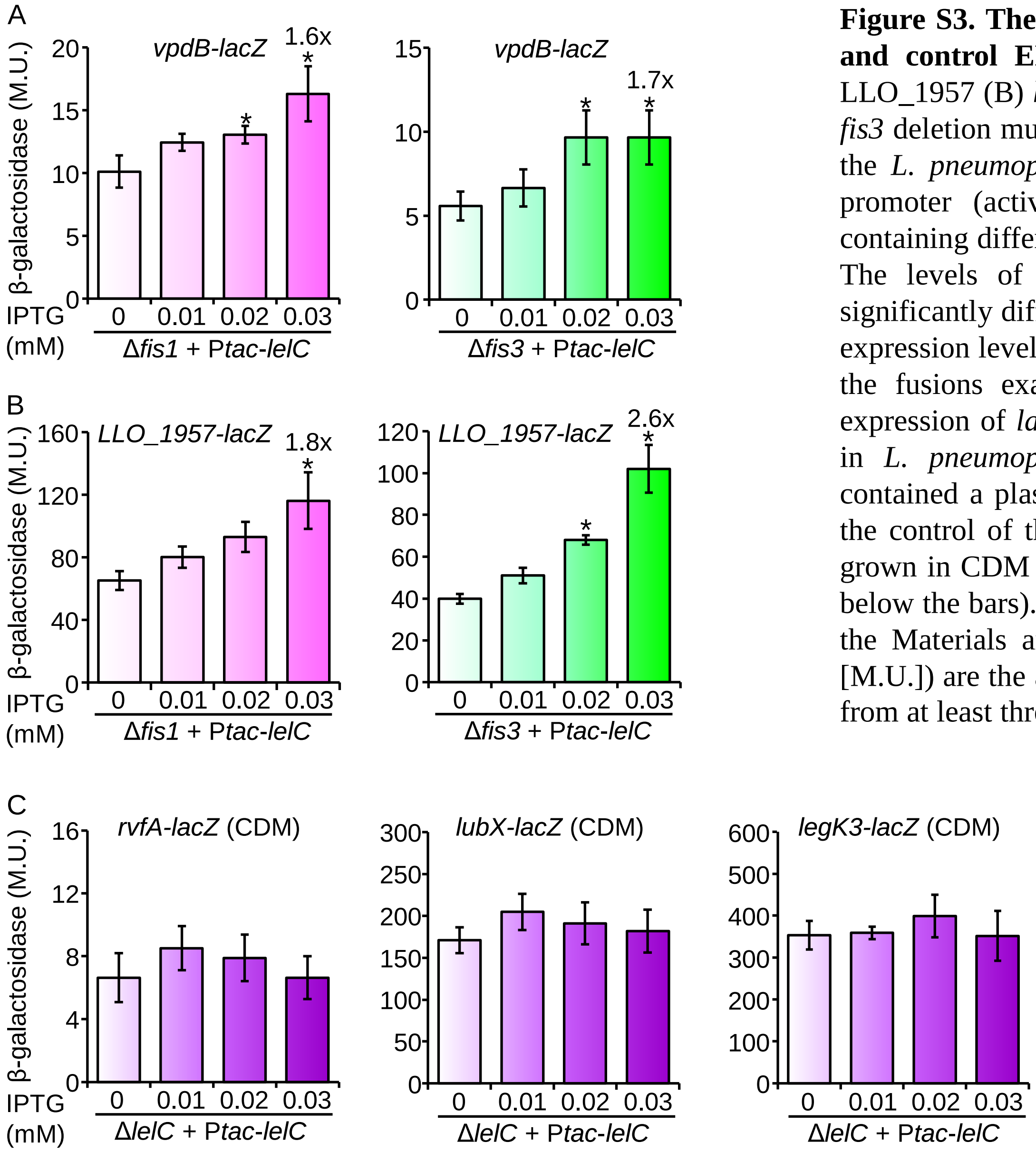

Figure S3. The effect of LelC on the expression of its target EEGs and control EEGs. A-B. The expression levels of *vpdB* (A) and LLO_1957 (B) *lacZ* fusions were examined in *L. pneumophila fis1* and *fis3* deletion mutants. The bacteria examined contained a plasmid with the *L. pneumophila lelC* gene cloned under the control of the P*tac* promoter (activated by IPTG), and they were grown in media containing different concentrations of IPTG (indicated below the bars). The levels of expression of the *lacZ* fusions were found to be significantly different (*$p < 10^{-4}$, unpaired Student's *t*-test) between the expression levels of the same *lacZ* fusions examined without IPTG and the fusions examined with different IPTG concentrations. C. The expression of *lacZ* fusions of *legK2*, *lubX*, and *legK3*, were examined in *L. pneumophila lelC* deletion mutant. The bacteria examined contained a plasmid with the *L. pneumophila lelC* gene cloned under the control of the P*tac* promoter (activated by IPTG), and they were grown in CDM containing different concentrations of IPTG (indicated below the bars). β-Galactosidase activity was measured as described in the Materials and Methods section. Data (expressed in Miller units [M.U.]) are the average ± standard deviations (error bars) of the results from at least three different biological replicates.

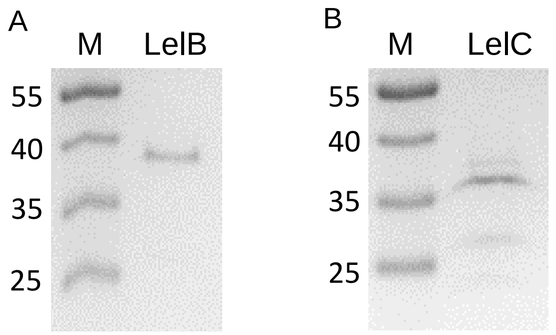

Figure S4. Purified recombinant proteins used for gel mobility shift assays. SDS-PAGE analysis of affinity-purified LelB-His$_6$ (37.6 kDa) (A) and LelC-His$_6$ (36.8 kDa) (B) visualized by Coomassie Brilliant Blue staining. Molecular weight markers (M) are indicated on the left of each panel in kilodaltons (kDa).

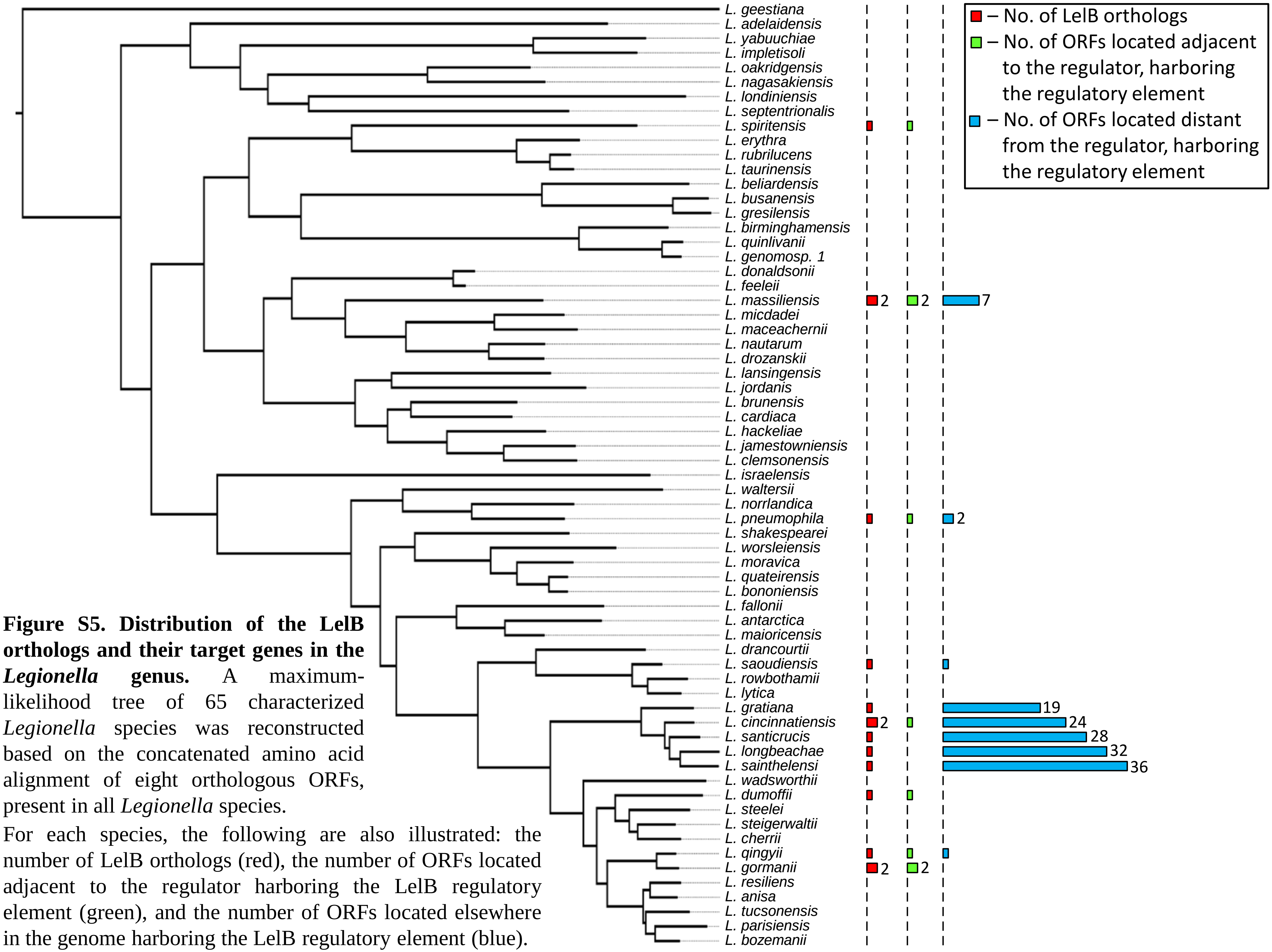

Figure S5. Distribution of the LelB orthologs and their target genes in the *Legionella* genus. A maximum-likelihood tree of 65 characterized *Legionella* species was reconstructed based on the concatenated amino acid alignment of eight orthologous ORFs, present in all *Legionella* species.

For each species, the following are also illustrated: the number of LelB orthologs (red), the number of ORFs located adjacent to the regulator harboring the LelB regulatory element (green), and the number of ORFs located elsewhere in the genome harboring the LelB regulatory element (blue).

```
Lpg2137        AGATATAACTAAATTTAATAAAACATATCAAAACAATTAATTGTATTAATATTTATCAATATGC-TCAAATACATGTA
Lspi_0405      TAACTTGCAAATCGTTCATATCAGATATCACCACAATTCATTGTACAACTATTAATCATAATGGATAAAATAATATCC
Lcin_2089      AATTATTACTAGAATTAATATCAGCTATTCATATAATTCACTGTACAAAAAAAACACCCATTGA-TTAAATATAAGCA
Lmas_RS06225   CAATATAACTAAAAGTTCTAAAGAGTATTAGTTTAATTCATTGTTCAAATTTAACTCAAGGTGA-TTAAATGTAATTG
Ldum_0172      TGATATTAAAAATTCTAATATGACATATCAGTACAAACTGTTGGATTTAAACTGAACCATTTGT-TTAAATACTGACG
PC997_RS03685  TGATATTAAAAATTCTAATATGACATATCAGTACAAACTGTTGGATTTAAAATGAACCATTTGT-TTAAATTCAGACG
Lqin_RS15820   AGATATCAAAATATTTAATATGATATATTCATACAATCAATTGGACGAATTTTGTTCTTTTGGTTTAAAATTCAATCT
Lgor_3248      CGATATTGAAGATTTAAATACCAGATATGAAGACAATTGATTTGCGCTCCAATAAAACCAAGTGGTTAAATATAGTAC
Lmas_RS04170   TGATATTCAAACAATAAATACCAGATATCAAGACAATTACTTTGCCTGCAAACAAAGCCAAGTGGTTAAATATAGCAC
Lgor_0297      CTCAATCAACATAATTAATATGACATATAAAAAAATATATTTTGATCTAAACAGCTGCATTTGG-TTAAATATTATTA
Lpg1797        ATTTATTACTAGAAGTGATATGAGATATCAAGATATTCGATTGGTCAACTAATAATCAATATGT-TTAAATTTATTTA
Lpg1101        GGATATTATTAAAAATAATATGACTTATGAAAACAATTAATTGTACATAAACAAATCATAATGA-TTAAATATATGCA
Lqin_RS15005   TGATATCAAAAAAATGGAAATGACATATTAATATAATTGATTGGATATACTTTGCTCAAAAAAATTAAAATGGCAACT
Lmas_RS07190   TGATATCGAAAAAAACTCTATGACATATTAATATATATAATTGGCTAAATATTGTTCAAAAAGGATAGAATAAGCCCT
Lmas_RS00240   ATGTATCATAAATTTGGATATGATATATCAACAAATATAATTAGATACTTTCGAACCCCTATAGCTAAAATACCTTCG
Lmas_RS03320   TGATATTAGAATTATGAATATGAGATATCGCCAGATTTCATTGGTCGTTTACTATTCTCTGTGT-TTAAATGTCGTGC
Lmas_RS07100   CCGCATCAAACTTATCAATATGACATATCAACACAAGTAATTGGATTTTTGTTGCTCTACATGGATAAAATTCAATCT
Lmas_RS07675   TCATATTAAGTCCCTCAATATGACATATCCAAACAAGTAATTGGATAAATATTAATCACCTTGG-TTTAATGTCTATT
Lmas_RS10135   CCTTATTAAAATCTTTAATATGACATATCAGCACAATCAATTGTATTAAAAACATTCTTTTAGA-TAAAATATACTTC
Lmas_RS17670   GGTTATTCAAACATTAAATATGACATATCAGTATAACTAATTGGATTTATATAGGTTTATTTGA-TTAAATCCGCCAA
Lsao_RS12735   GCTTATTTTAGAAACATATAGTATATATCAAGCCACATAGTTCGTCATTAATGGCGAATAAATC-TCAAATAGTAATT
                  TATnnnnnnnnTTnATATgAcATATnAAnnnnnnnnnATnnnnnnnnnnnnnnnnnnnnnnnn-TnnnnT
```

Figure S6. The regulatory region of putative LelB-regulated genes from *Legionella* species harboring LelB orthologs from clades I and II. The putative -10 promoter elements are in blue, the nucleotides representing the common LTTR motif are in red, additional conserved nucleotides are in bold, the inverted-repeat sequence is marked by arrows, the putative Fis regulatory elements are shaded in yellow and their conserved nucleotides are in purple. The genes are indicated by their locus tag numbers, and the locus tags of the genes located adjacent to the regulator are indicated in bold. The prefix of the locus tags is the following: *L. massiliensis* - BN1094_RS# (Lmas), *L. qingyii* - ELY16_RS# (Lqin), *L. saoudiensis* - BN3124_RS# (Lsao), and the uncharacterized species PC997 - HBNCFIEN_RS# (PC997).

Figure S7A

```
LLO_0038   ACATATTTGTACAATATATATCACATATAAACACAATGAATTGTACAAATGATTTTCATGATGATTAAAATACAA
LLO_0087   TAGTATCGATTAATTTGATATGACATATTAATATTACTAATTGGATTAATTAAGATCGTTCCGA-TTATATAGCG
LLO_0095   GAATATCAAATAAATTGATATGACATATGATGATAATCAATTGTACAAATACAGGCCTTGGTGA-TTAAATTACG
LLO_0245   TGATATCAATTTAATCAATATGATATATAAACATAATGAATTGTACAAATAGGCGCCTTAATGT-TTGAATTATG
LLO_0614   AGTTATTTGTAAATTTCATATCACATATTAACAAATTTTATTGGCAAAAAAAATATCTATTTGTGTAGCATTTCG
LLO_0706   ATATATTTTTCCTATCGATAACACATATAAAAACAATCAATTGGATAACTTCAATCTTGAGTGATTAAAATATGA
LLO_0990   TATTATCAAAATATTTGATATGACATATAAGTAGAATTAATTGGATTGATTTGTTCAATTTGAA-TAAAATTTGA
LLO_1060   TAATATCTATCTTATTTATATGACATATAAAAACAATAACTTGGATATTTTTTATGCAACACAATTATAATTAGA
LLO_1123   AATTATTTATAAGTTTTATATGATATATCAATATAATAAATTGGTTAAATATTGCATAAAGTGA-TTAAATAAAA
LLO_1372   TCATATCAAATAATTTGATATGACATATAAATATAATTAATTTGATTTGCTTAGTGCCATCTGGCTAAATTTAGT
LLO_1397   AGATATGAAAAGTTTTCATAATTCTTATGAAAAACCTTGATTGGCGCTTTGTTGATCATTAGTGATAAATTAAAC
LLO_1404   ACATATTTAACAAACATATATCACATATAAACATAATCAATTGGAATTATTTTGGTCAATTGGA-TTAAATATCA
LLO_1454   TGTTATTTACATATTTAATATAACATATAAATTTAATCAATTTGATTATATTTAATCCATGTCGATAATATTTAA
LLO_1473   CATTATTTGTAAAAATTATATGACATATTAAAACAATTAATTGGTATTATATGTCTCATTTTGA-TTAAATTTTA
LLO_1506   TACTATTTGTATATTTCATATCATATATGAGTTTTATTAATTGGATTTTATGTAATCATATTGA-TTAAATTTAT
LLO_1715   AAGTATTTGAATGTTTAATATGATATATAAAAATAATGAATTGGATAAAATTGGCTCATGATGAGCATAATATCA
LLO_1737   AATTATTTATAAATCTTATATGACAAATCAAATTAACAAATTGGACTAAAAATGACCCTCATGGTTAAAATGAAT
LLO_1741   TAGTATTAGTAATTTTCATATTGCATATAGATATTTCAAATTGGATTAAATTTAACAACAATGA-TTTAATCTAT
LLO_1917   ACATATTTGTATCCTGTATATGATATATAAAAATAATCGATTGTACTTAATTGAATCATGATGACTATATTATGG
LLO_1995   AGTTATAAAATTTTTTGATATAACTTATGAATAAAATTAATTTGATGAATATATGGTGTTATTGCTTTAATAATC
LLO_2109   ACTTATCAATTTTTTTGATATGACATATAAAATAATCTAATTGGTCAAATATGAGAAACATTGT-TCTAATTACC
LLO_2209   TATTATTTGCACATTTGATATGTGAATTAAGTATTTCTAATTGGACACATACACACCTTATTGA-TTTAATGTTA
LLO_2240   TAATATTTGAAATTTTCGTATAACATATAAGAACAATCAATTGGATATTGATTTAACTTAGTGA-TTAAATTAAG
LLO_2329   AGATATAAAAATAATTCATTACATATATAAAAAAAATCAATTATTCAAATACAAATCATGTTGC-TTTAATGCTT
LLO_2440   TAATATTTAATTTATTTATATGTCATATGAATTTAACAAATTGAGAAATCATGGAACATATAGGATAAAATAATT
LLO_2558   AACTATTTAATAGTTTCATATCATATATACACCCAATGAATTGGACTCATTTTGACCAGCATGA-TTAAATATGA
LLO_2746   TGTTATCTAAAAAATTTATAACTCATATAAATTGAATCAATTGGATTAATGTAGATCAATTTGA-TTAAATATAA
LLO_3042   AAATATCAATTTTTCTAATATCATTTATAAAAAATCAAATTGGCAATAAAATAATCTTGATGG-TTAAATATAA
LLO_3086   AGATATATAACCTGTTGATATGACATATTCATAAAATCAATTGGATTGATTTTGATCCCTATGG-TTAAATATTT
LLO_3245   AGATATCTAAACCCTAAATATGACTAATAAAATTTTCAATTGGAAAATTATTGTTCAGTACTGATAAAATCAAA
LLO_3386   TTATATTTGTTGAGTTGATATCACTTATAAATACAATCAATTGGATTAAATATAAACATAATGA-TTAAATTTAT
LLO_4077   AAATATTAAATAATTTGATATCCCATATTAGTATAATCAATTTGATTTGTAAGTTCATGTGCGT-TTAAATTTTA
              TATnnnnnnnTTnATATgAcATATnAAnnnnnnnnATnnnnnnnnnnnnnnnnnnnnnnn-TnnnnT
```

Figure S7B

```
Lsai_0081   TAATATTTAAAATATTAATATGTCATATGAATATAATCACTTGTCAATATATACATCAAAAAAGATAAAATATAT
Lsai_0267   AATTATTTGAATGTTTAATATGATATATAAAAATAATGAATTGGATAATATGTGCCCACAATGAGCATAATATGA
Lsai_0288   AATTATTTATAAATCTTATATCACAAATGAAATTATCAAATTGGATTTAAAATGTCTTTTTTGGTTAAAATAAAT
Lsai_0397   TATTATTTGTTCGTTTGATATATGAAATAAGTATTTTTTATTGGACTAAAAATAATCCTTTAGA-TTTAATGTAT
Lsai_0428   TAATATTTGAAATTTTCGTATAACATATAAGAACAATCAATTGGATATTAATTGATCTTAATGTTTAAATTAAGT
Lsai_0488   ACATATTTGAAAAATGTATATGATATATAAATACAACTCATTGTACAAAAATAAATCTAAAGAT-TAAATTAAAA
Lsai_0542   GACTATCAAATTATTTAATATGACATATAAGAATAATTGTTTGTACTTATTTAGTGTTTGCTGG-TTAAATATTG
Lsai_0659   GATTATCAAATTTATTAATATCATAAATGAATATAATTTATTGGAATATTAACCCTTAAATTTGATTAAATATAA
Lsai_0676   AGATATGAAAAATTCTCATAATTTTTATGAAAATTCCCGATTAGCCAATTAATATTCAATCATGATAAATTCAAA
Lsai_0716   AATTATTGAATCGTTTCATATCATATATAAATCCAATGAATTGGACTGGTTGTGTTCAAAATGA-TTAAATATGA
Lsai_0772   ACATATTTGCACAATATATATCACATATAAATAGAATGAATTGTACAAATAATTCTCATGGTGA-TTAAATTATA
Lsai_0823   TACTATCAAATCATTTGATATGACATATTAATATAACTAATTGGATTGATTAAGCTCGGTCCGG-TTATATAGCG
Lsai_0828   AAACATCAAATTATTTGATATGACATATCAAGATTTCCTATTGGCCTAGACAATTTTTAATCAG-TTAAATAAAC
Lsai_0927   AGATATTAGTTAATTCAATATGATATATAAATATAATTAATTGTTCAAATATCAATCTTAGTGC-TTGAATTATG
Lsai_1210   ACATATTTGTATCTTGTATATGATATATCAAAACAATTGACTGTACTTATTAATAACAGAATGTTTATATTAATT
Lsai_1466   TATTATCAAATTATTTGATATGACAAATAAAAAAAATTAATTGGTTTAACTAATTTATTTTGGA-TAAAGTTGA
Lsai_1492   AATTATTAAAAAATCTCATATGAAAAATCAAAACATTTGATTGGTTTTAAATTAACCATTTAAT-TAAAATTATG
Lsai_1671   AGAAATTTGTAGATTTCATATGATATATAAATAAATTGAATTGGATGGTATTTGTTCATAATGA-TTAAATATTA
Lsai_1673   GAGTATTAACCCTTTTTATATGACATATCAACACAATGAATTGGCTATTTTTAGCTCAAAGAGT-TAAAAATTTA
Lsai_1773   AATAATTTATTTTTTTGCTATCACATATACATATATTGAATTGGATTAATATTGCTCTATAAGATTTAATTGTAC
Lsai_1837   CTTTATCAATAAGATTGATATGAAGTATGAATACAATCAATTGTATTTATTTTAATCTTTGTGG-TTTAATTTGA
Lsai_1862   AGATATATAATATGTTCATATGATATATTCATAAAATCAATTGGATTGATTTTTGTCTATATGA-TTAAATAATT
Lsai_1967   AAATATGAATTAAATTCATATGATAAATCAAAATATTCAATTGGCCAATAATTGCACATAAAGATTATCATTAGG
Lsai_1990   TCATATTAAAAAAATTGATATGAGATATAAATATTATCAATTTTACTTAAATCGATCTTTGTGAATAAAATATGT
Lsai_1991   CATTATAAGTAAAATTCATATGTCATATAAATATTATTAATTGGAATTATTTTGGTCAATAAGT-TTAAATTTAA
Lsai_2153   ACATATTTATTCCAATTATATGACATATAAATTTAATTAATTGTAATTTATTTAAGCAAATAGA-TAAAATTAAT
Lsai_2349   TCATATTTGCTGAATTGATATCACTTATAAATACAATCAATTGGATTAAATATAAACACAATGA-TTAAATACAT
Lsai_2426   CTTTATCAGGCCAATTGATATGAGATATAAATAAATTGCATTGTAAAAATATTTATCTTAATGC-TTAAATATTA
Lsai_2658   AGATATTAAATTATTTGATATCCTATATTAATATAATCAATTTGATTTACAAGATAAGAGATGT-TTAAATTTTA
Lsai_2857   AGATATCAAATAATTTAATATGTCTTATAAATTACATTGATTGTACATATTTAATTAAAAATGA-TTAAATTTAA
Lsai_2881   CAATATCTATCTTATTTATATGACATATAAAAACAATGACTTGGATATTTTTTACTCAATCTAATTATAATTAGA
Lsai_2938   AATTATTTGTAAATTTTATATGATATATCAATATAATAAATTGGTTAAATATTGAGTAAAGTGG-TTAAATAAAA
Lsai_3224   ATATATTTATCAATTTCATATAATATATAAGAACATTCTATTGGATTTATATTGCACTTATTGGATAGTATTTAA
Lsai_3463   TCATATCAAAATATTTGATATGACATATTAAAAGATTGCTTAGTCTTAAAAATCATTTGTTGG-TTAAATATAG
Lsai_3469   AATTATTAGTTTTTTTGATATGACATATAAATTAATCTAATTAGTTATAAAAATAAAAATTTGA-TTGAATATGG
Lsai_3483   ACATATTTAACAAATAGATATCACATATAAACATAATCAATTGGAATTATATTGTTCAATTGGA-TTAAATAGTA
               TATnnnnnnnnTTnATATgAcATATnAAnnnnnnnnATnnnnnnnnnnnnnnnnnnnnnnn-TnnnnT
```

Figure S7C

```
Lsan_0225   AATTATCAAATTATTTGATATCACATATAAATACATCTCATTGGTTATTTTAATCTCACTTTGG-TTAAATTGCG
Lsan_0813   CATTATTCAAAATTTTCATATGGTATATAAATATAATCAATTGCACAAATAATGATCTGCATGA-TTAAATGTTG
Lsan_0856   AATTATTTATAAAATTTATATGATATATCAATACAATAAATTGGTTAAATATTAAATAAAATGA-TTAAATATAA
Lsan_1213   AGATATATAACGCTTTGATATGACATATAAACATAATCAATTGGATTGTTTTTGCTCTATATGG-CTAAATAATA
Lsan_1632   TATTATTTGTAAATTTCATATCACATATAAACAAATTTTATTGGCAAAAAAAAGACCTATTTGTGTAGTATTTCA
Lsan_1808   TACTATTTAATAATTTAATATGACATATTAATATAATTAATTGGATTGATTGAACTCAGTCCGG-TTATATAGCG
Lsan_1835   TAATATAAAATTATTTGATATGACATATAAGTATAATTTATTGTACTTATTAAGATTTTATTGG-TTAAATAGTA
Lsan_1873   AACTGTTTAATAATTTCATATCATATATAAATCCAATGAATTGGACTCATTTTGATCAACATGA-TTAAATATGA
Lsan_1904   TAATATTTAAAATATTAATATGTCATATGAATATAACAACTTGTTAATAGATACATCAAAAAAGATAAAATATAT
Lsan_1926   ACTTATTTAATAATTTCATATCATATATAAATTCAATTCATTGGATTTATATTGATTAAATTGG-TTAAATAAAA
Lsan_1942   AGATATTTAGAAATCTTATATGACAAATAAATACAATTGATTGGATATATATCGCTCTGGATGTATAAAATAATA
Lsan_2248   GCATATGAAAAATTTTCATAATTCTTATGAAAAATCCTCATTGGTACATTTTAACCCATTAATGATAAATTTAAT
Lsan_2656   GCATATTAAATTATTTAATATCACATACCAATAATTCTCATTGGTTTTTATTTGATTATTTTGA-TTAAATTAGC
Lsan_2722   ATATATTTGCCGAATTGATATCACTTATAAATACAATCAATTGGATTAAATATGAACACAGTGA-TTAAATACTT
Lsan_3057   TAATATCTATCTTATTTATATGACATATAAAAACAATAACTTGGATATTTTTTACTCAACACAATTATAATTAGA
Lsan_3152   TAATATTCGAAATTTTCGTATAACATATAAAAACAATCAATTGGATATTAATTTAACTTAATGT-TTAAATTAAG
Lsan_3243   ATATATTTAACAGGCGTATATCACATATAAACATAATCAATTGGAATTATATTGCTCAATTAGG-TTAAATAGTA
Lsan_3284   TGATATTTACATATTTAATATGATATATAAAAATAATCAATTTGATTTATGAGAAATCTATCTGATAATTTTTAG
Lsan_3301   CCTTATTTGTAAAAGTTATATGTCATATAAAAACAATCAGTTGGTCATATATATACCAATATGA-TTAAATATAA
Lsan_3490   CCTTATCAAATTATTTGATATCAGATATAAATATATCCAATTAGATTTAATTGATTTATTTTGA-TTCAATTAAG
Lsan_3518   ACATATTTGTATCTCATATATGATATATAAAAACAATTAATTGTACTTATTTTACCCATAGTGA-TTAAATTAAA
Lsan_3541   AATTATTTATAAATCTTATATGACAAATGAAATTATCAAATTGGATTTAAAATGTCTTTTTTGATTAAAATAAAG
Lsan_3545   TAGTATTAGTAATTTTCATATTGCATATAAAAATTTCAAATTGGATTGAAATAACCAACAATGG-TTTAATTTAA
Lsan_3616   AAGTAACTAATTTTTTTATATCACATATAAATACATTTAATTGGATTAATCGAGATCTGATTGTTTTAACTATGA
Lsan_3805   TATTATCAAATTATTTGATATCCAATATAAATACAATCAATTTGATTTTAGATCTCATTGTATG-TAAATTCTAC
Lsan_4005   AGTTATTAAATATTTTGATATAACATATGCATATATTAAATTGGACTAATATTGCACTATACGA-TTTAATGGTA
Lsan_4087   ACATATTTGCCCTATCAATAACACATATAAAAACAATCAATTGGATAATTTCGATCTTGTTTGA-TTAAATTTTA
Lsan_4176   TGTTATCAAATTATTTGATATGACATATAAATTTAATTAATTTGATTTACTTAATGCCTTTTGGCTAAATTTAGT
               TATnnnnnnnnTTnATATgAcATATnAAnnnnnnnnATnnnnnnnnnnnnnnnnnnnnnnn-TnnnnT
```

Figure S7D

```
Lcin_0095   ACATATTTAACAAATATATATCACATATAAACATAATCAATTGGAATTATATTGGTCAATTAGA-TTAAATAGAA
Lcin_0210   TATTATTTGTAAATTTCATATCACATATTAGCAAATTTTATTGGTAAAAAAAACGTCTATTTGTGTAGCATCTCG
Lcin_0244   CATTATTTGAATCTTTTATATCACACATAAAAATTATCGATTGGATTATTATTGGGTTAATTGA-TTAAATTATG
Lcin_0276   ATATATTTATCTATTTCATATAACATATAAAAACATTTAATTGGATTATTATAGACCTTATTGCATAACATTTAA
Lcin_0370   GGTTATTTGTAAAAATTATATGACATATAAAAATAATTAATTGGATTTGTATTAACCAATAAGATTAAACTATAA
Lcin_0588   CATTATTAGCAAAAATTATATGACAAATGAAAATATTTTATTTGTCATATAAAATCATTTTGAT-TAAATTTAAA
Lcin_0614   TCATATCAAAATATTTGATATTACATATTCGAAAAATTGCTTTGTTTTAAAAAGCATTAATTAA-TTAAAATGAA
Lcin_0754   CATTATTTAAAATTTTCATATGGTATATAAATACAATCAATTGTGCTAATAATCACCCGCATGA-CTAAATATTG
Lcin_0957   CATTATCGAATAATTTGATATGACATATTAATAGAATTAATTGGATTGATTGAGATCGGTGCGG-TTATATAGCG
Lcin_1020   AGTTATGAATTTTTTTGATATGAGATACAAATAAAACTAATTGGATTTTGTGTGGCTTAACTGA-TGAAATTGTT
Lcin_1052   AAATATTAAAATGGTTGATATGACACATAAAATTAATTGATTGGTTATTTTTCAAGATAAATGA-TTTTATATTA
Lcin_1066   AATTATCAAATATTTTGATACAACATATACATATATTAAATTGGACCAATATTACACGATAAGA-TTTAATAGAA
Lcin_1968   TGTTATCTAAAAATTTTATAACTCATATAAATATAATTAATTGGATTAATATTAATCATATTGA-TTAAATATAA
Lcin_2089   AATTATTACTAGAATTAATATCAGCTATTCATATAATTCACTGTACAAAAAAAACACCCATTGA-TTAAATATAA
Lcin_2244   TAATATTTAAAATATTTATATGTCATATGAATATAATAACTTGTTAATATACAGCCCAACCAGAATAAAATATAT
Lcin_2339   GTTTATTTAAAAGATTCATATGAGAAATCAAAATACTCAATTGGATTTAATTTGATCACATTGT-TAGTATATTA
Lcin_2353   AGCTATTTAATAATTTTATATCATATATAAATTCAATGAATTGGACTCATTTTAGACAAAATGA-TTAAATATGA
Lcin_2397   AATTATTTATAAATTTTATATGATATATCAATATAATAAATTGGTTAAGTATTGAATAAAATGG-TTAAATAAAA
Lcin_2935   AACTATTAAATTATTTGATATCACATATAAATAAAATCAATTAGATTTATTGCGATCACTTTGG-TTAAATTAAA
Lcin_2947   AGTTATGAATTTTTTTAATATGACTTACGAATAAAACTAATTGGATTAATTAAGGTTAAATTGA-TTAAATATTT
Lcin_3034   CGATATCAAATAATTCAATATCTCTTATAAATATAACTGATTGTATTTTATTAGTAGATAATGG-TTAAATAAAA
Lcin_3077   AGATATGAAATTATTTAATATGACATATCAATAGAATTAATTGGAACTTTGAATTATAAATTTGGTTTAATTGCA
Lcin_3214   AAATATTTGAATGTTTAATATGATATATAAAAATAATGAATTGGATAATTGATGAACATAATGAGCATAATATGA
Lcin_3436   AGATATATAACGCTTTGATATGACATATAAACAGAATCAATTGGATTATATTTGCCCTATATGG-CTAAATAATA
               TATnnnnnnnTTnATATgAcATATnAAnnnnnnnnATnnnnnnnnnnnnnnnnnnnnnn-TnnnnT
```

Figure S7E

```
Lgra_0289   GACTATTTATCAATTTGGTATGACATATAAATTTAATCAATTGGATGTTTTTATACTTACTGG-TTAAATAAAA
Lgra_0492   GAATATTAAATAAATATATATCACATATAAAGATAATCAATTGGATTTATATTAATCAATTGGA-TTAAATAATG
Lgra_0649   AATTATTTATAAATTTCATATGAGAAATCAAAATGTTTAATTGGATTAAAATTGCCCAATTAGACTAATATGAGG
Lgra_0861   AATTATCTAAAAAATTCATATGAGATATAAAAACATTCAATTGGATCAATAATCAACACTATAG-TCAAATGATT
Lgra_0977   TATTATTCAAATTTTTCATATCGTATATGAATACAATCAATTGTACAAATAATGATCTGCATGA-TTAAATATTG
Lgra_1152   TAGCATCAAAAAATCTCATATGACATACAAATAAAACCAATTGTACAAATAACAAATATGATGA-TTAAATATTT
Lgra_1359   TGATATCAAATAATTTTATATCATATATCAAAATAATTTATTAGATTTATTAAATTATCTTTGG-CTAAATGCTT
Lgra_1713   AGTTATTTGCAATCCTTATATTATATATAAATACATTCGATTGTATTAATTTAAATCTAGATGG-TTAAATATAT
Lgra_1745   CAATATTCAATTCATTGATATAACATATAAACACAATCAATTGTACTAATATTAATCTTTATGA-TTAAATAATA
Lgra_2006   AGTTATTTGAATATTTAATATGATATATAAAAATAATGAATTGGATAAATAGTAATCGTAATGCTCATAATGTGA
Lgra_2035   AATTATAAGAATGATTGATATGACACATAAAATTAATCCATTGGTAATTTTTTTGGAAAATGCTTTTATTTTAAT
Lgra_2049   TGTTATTTATCTTCTTCATATGATATATAAAAATAACCATTTGGATGTTTTTTGATTAATATGATTATAATTGGA
Lgra_2129   AGATATTTAATTGTTTGCTATGACATAGAAATACGTTCTATTGGTTAAATTATCACCATAATGC-TTAAATAAAG
Lgra_2260   AATTATTTAAACGATTCATATGTGAAATCAAAATATCTCGTTGGAATTAATTTGCTCATATTGACTAATATAATA
Lgra_2446   AATTATCAAATTATTTCATATCATATATCGATATAATTTATTTTACTTACTGACTAAATATTGA-TTAAATAGTA
Lgra_2470   AAATATTTAAAATTTTCGTATAACATATAAAAACAACCGATTGGATATTAATTTTACTTAATGT-TTAAATTAAG
Lgra_2687   TATTATTAAATTATTTGATATCCAATATGAATAAAATCAATTTGATTTGCGATTCCTGGGTGTG-TAAATTTTAT
Lgra_2869   AACTATTAAAAAAATTGATAATAAATATAAATACAATCAATTTTACAAATATATCTCTTGATGG-TTAAATATTT
Lgra_3177   ACATATTTGCACAATATATATGACATATAAAAACAATCAATTGTACACATATTAGCCAAGATGG-TTAAATGATA
               TATnnnnnnnnTTnATATgAcATATnAAnnnnnnnnATnnnnnnnnnnnnnnnnnnnnnnnnn-TnnnnT
```

Figure S7. The regulatory region of putative LelB-regulated genes from *Legionella* species harboring LelB orthologs from clade III. The regulatory regions of genes harboring the LelB regulatory element identified in *L. longbeachae* (A), *L. sainthelensi* (B), *L. santicrucis* (C), *L. cincinnatiensis* (D), and *L. gratiana* (E). The putative -10 promoter elements are in blue, the nucleotides representing the common LTTR motif are in red, additional conserved nucleotides are in bold, the inverted-repeat sequence is marked by arrows, the putative Fis regulatory elements are shaded in yellow and their conserved nucleotides are in purple. The genes are indicated by their locus tag numbers.

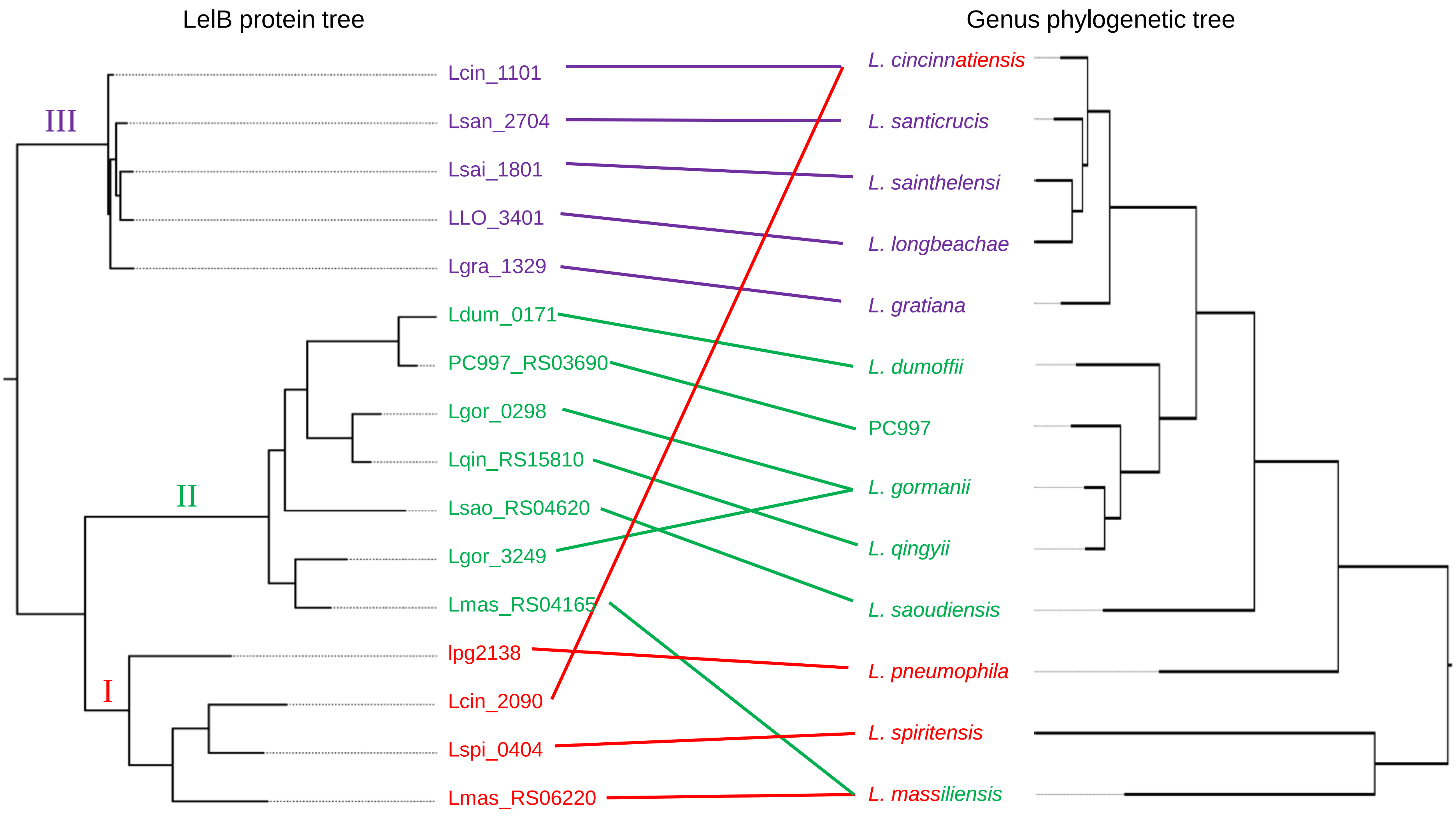

Figure S8. LelB orthologs from clades I and II undergo HGT in the *Legionella* genus. A. A maximum-likelihood phylogenetic tree of the amino acid alignment of the LelB orthologous ORFs from the 13 *Legionella* species harboring the regulator. The protein sequences used are identified by the locus tags as follows: *L. saoudiensis* - BN3124_RS04620, *L. massiliensis* - BN1094_RS06220, and PC997 - HBNCFIEN_RS03690. B. A maximum-likelihood tree of 12 characterized and one uncharacterized *Legionella* species was reconstructed based on the concatenated amino acid alignment of eight orthologous ORFs, present in all analyzed *Legionella* species. The orthologs belonging to each clade are marked with a different color: clade I - red, clade II - green, and clade III - purple. Each LelB ortholog is connected to the corresponding *Legionella* species on the phylogenetic tree.

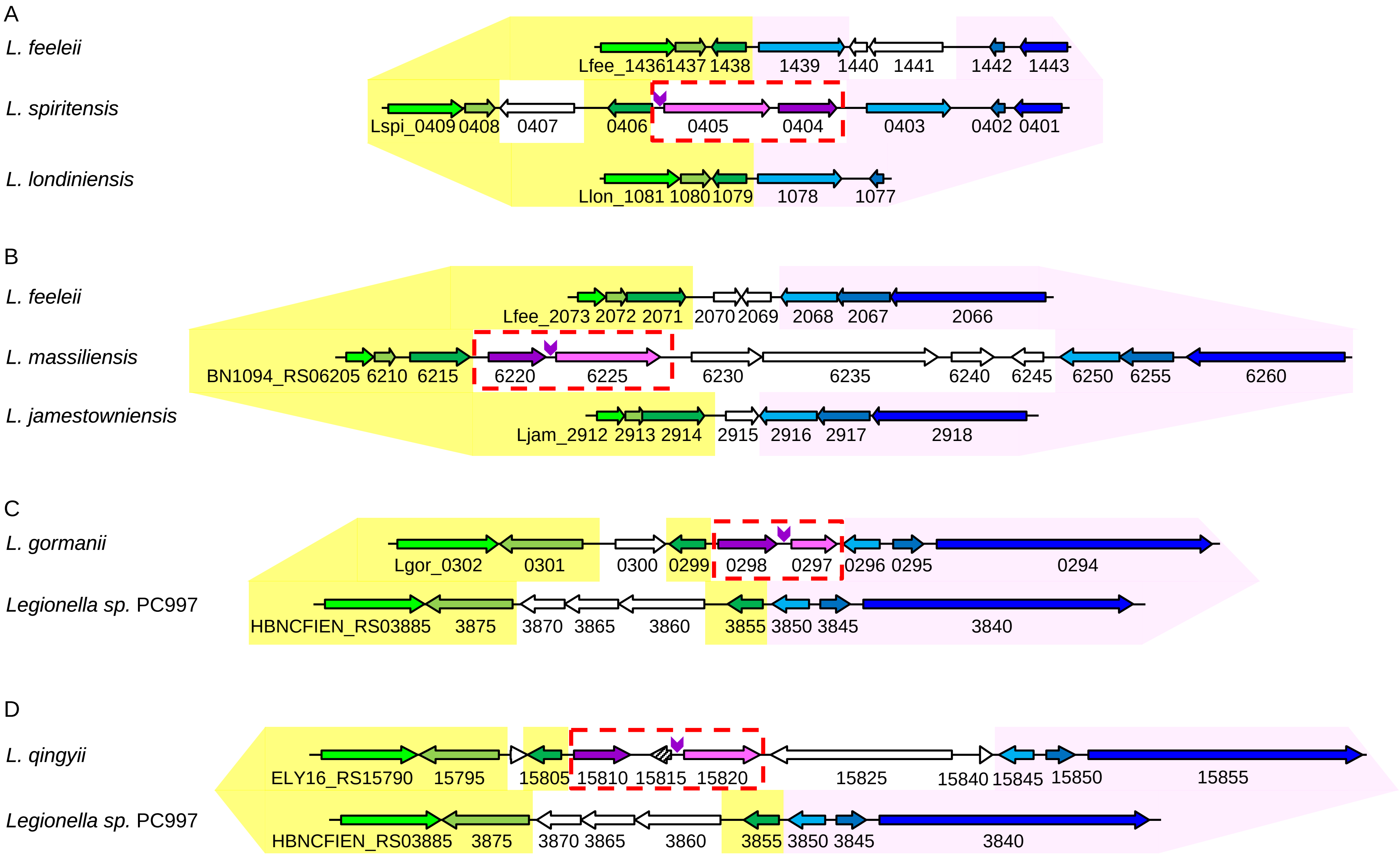

Figure S9. LelB orthologs from clades I and II are found as genomic islands together with their putative target genes. Schematic representation of the the genomic region adjacent to the *lelB* orthologs in *Legionella* species both harboring and lacking the regulator. In all panels (A-D), the *lelB* orthologs are marked in purple and their putative target genes in pink, homologous genes within each panel are marked by the same color, non-homologous genes are shown in white, insertion sequence is hatched. The genes are identified by their locus tag number. The position of the conserved regulatory element predicted to be recognized by LelB is indicated by purple arrows pointing downward. Yellow (left) and pink (right) shading highlights similar genomic regions, with the *lelB* ortholog and its putative target gene enclosed in a red box.
