# Supplementary material for: Versatile regulation of effectors by novel orthologous regulators in the Legionella genus
Source: mBio. 2025 May 30;16(7):e01268-25. doi: 10.1128/mbio.01268-25 (PMC12239595; doi:10.1128/mbio.01268-25)
Supplement: Supplemental data sets — Data sets S1 to S5. [file mbio.01268-25-s0002.pdf]

Dataset S1. Properties of proteins encoded by genes harboring the LelB regulatory element from *Legionella* species harboring LelB orthologs from clade I and II

***Legionella* sp.**	**Locus tag #**	**Protein**	**Effector domains**	**Orthologs with a site ***	**Orthologs without a site ***	**Size (AA)**
L. pneumophila	**Lpg2137**	LegK2	STK	-	-	538
	Lpg1797	RvfA	CC	-	Lmas_RS05470, Lqin_RS06570, Lgor_2979	426
	Lpg1101	Lem4	HAD, PI4P	-	-	322
L. spiritensis	**Lspi_0405**	Lspi_0405	Ank (LegA2)	-	LegA2	554
L. cincinnatiensis	**Lcin_2089**	Lcin_2089	C58	-	-	483
L. dumoffii	**Ldum_0172**	Ldum_0172	-	**Lgor_0297, PC997_RS03685**	-	227
L. gormanii	**Lgor_3248**	Lgor_3248	PP2C, U-box	**Lmas_RS04170**	-	1859
	**Lgor_0297**	Lgor_0297	-	**Ldum_0172, PC997_RS03685**	-	228
L. qingyii	**Lqin_RS15820**	WP_110144116	Ank	-	-	401
	Lqin_RS15005	WP_126955726	-	Lmas_RS07190	Lgor_2392, Lqin_RS01385	568
Legionella sp. PC997	**PC997_RS03685**	WP_182392736	-	**Ldum_0172, Lgor_0297**	-	227
L. massiliensis	**Lmas_RS06225**	WP_043873418	CC	-	-	550
	**Lmas_RS04170**	WP_043873087	PP2C, U-box	**Lgor_3248**	-	1780
	Lmas_RS07190	WP_043873580	-	Lqin_RS15005	Lgor_2392, Lqin_RS01385	569
	Lmas_RS00240	WP_043872439	-	-	-	243
	Lmas_RS03320	WP_043872946	-	-	-	256
	Lmas_RS07100	WP_043873563	Ank (LegA8/AnkX)	-	LegA8/AnkX	506
	Lmas_RS07675	WP_043873867	-	-	-	64
	Lmas_RS10135	WP_043874282	-	-	-	696
	Lmas_RS17670	WP_044012530	-	-	Ldum_3189	323
L. saoudiensis	Lsao_RS12735	WP_058535162	-	-	-	99

Orthlogs marked in bold are located adjacent to the LTTR encodig gene

* Orthologs from *Legionella* species harboring the regulator

# The prefix of the locus tages are the the following: Lmas - BN1094, Lsao - BN3124, PC997 - HBNCFIEN and Lqin - ELY16.

STK - Serine/threonine protein kinase domain

Ank - Ankyrin repeat

CC - Coiled-coil domain

PI4P - DrrA phosphatidylinositol 4-phosphate binding domain

PP2C - Protein phosphatases, family 2C

U-box - E3 ubiquitin ligase domain

C58 - C58 peptidase domain, found in the *Legionella pneumophila* effector LegA7, *Pseudomonas* effector AvrPphB and the *Yersinia* effector YopT.

HAD - Haloacid dehydrogenase domain

Dataset S2A: Properties of *L. longbeachae* proteins encoded by genes harboring the LelB regulatory element

Locus tag	Neighaboring genes	Effector domains	Size (AA)	%GC	Signal score	Machine learning score	No. of sp. with site	Lcin	Lsai	Lsan	Lgra
LLO_0038	LLO_0037 - Ank	**STK**	599	37.5	8.381	1.0000	3	Lcin_0283	Lsai_0772 Lsai_0637 Lsai_1210	Lsan_3518	-
LLO_0087	LLO_0088 - Coiled-coil	**Ankyrin repeat**	817	40.6	3.961	0.9287	4	Lcin_0957 Lcin_3077	Lsai_0823 Lsai_0156	Lsan_0225 Lsan_1808 Lsan_2700 Lsan_3490	Lgra_2953
LLO_0095	-	-	375	33.4	1.113	1.0000	1	-	-	-	Lgra_3272
LLO_0245	LLO_0246 - Ank	-	732	34.6	3.912	1.0000	2	-	Lsai_0927	-	-
LLO_0614	-	**Colied coil**	783	37.2	7.083	1.0000	3	Lcin_0210	Lsai_1515	Lsan_1632	Lgra_0790
LLO_0706	-	-	98	34.7	-6.150	0.0370	2	-	-	Lsan_4087	-
LLO_0990	LLO_0991 - Rho-GAP	**Ankyrin repeat**	577	35.3	0.780	0.6191	2	-	Lsai_1466	-	-
LLO_1060	-	IcmD (DotP) domain	133	39.1	0.164	0.0030	4	Lcin_1547	Lsai_2881	Lsan_3057	Lgra_2049
LLO_1123	-	**Coiled-coil**	608	33.2	6.324	1.0000	4	Lcin_2397	Lsai_2938	Lsan_0856	-
LLO_1372	LLO_1371 - Ank	**SidC N-terminal domain**	953	36.3	1.918	0.9997	2	-	-	Lsan_4176	-
LLO_1397 - RalF	-	**RalF (GEF)**	384	31.8	3.793	0.9985	3	-	Lsai_0676	Lsan_2248	-
LLO_1404	LLO_1403 - CetLl3	PPR repeat	470	34.3	-6.114	0.0543	5	Lcin_0095	Lsai_3483	Lsan_3243	Lgra_0492
LLO_1454	-	**Rab GTPase (LepB)**	519	35.2	-0.296	0.9455	2	-	-	Lsan_3284	-
LLO_1473	-	**Coiled-coil**	430	33.6	2.712	0.9997	3	Lcin_0370	-	Lsan_3301	-
LLO_1506 - CetLl4	-	PCRF domain	324	33.5	7.502	0.9690	1	-	-	-	-
LLO_1715	LLO_1716 - RleA (Rho GTPase)	**Ankyrin repeat**	343	35.2	-4.312	0.4768	3	Lcin_0001 Lcin_0069	Lsai_0267	Lsan_2172 Lsan_2173	Lgra_2006
LLO_1737	-	-	372	32.1	2.921	1.0000	4	Lcin_0244	Lsai_0288	Lsan_3541	-
LLO_1741	LLO_1742 - Ank	**Coiled-coil**	837	35.0	7.784	1.0000	2	-	-	Lsan_3545	-
LLO_1917	-	**STK**	320	32.4	-3.678	0.9980	1	-	-	-	-
LLO_1995	-	**F-box**	316	37.0	-0.179	0.9852	3	Lcin_1020 Lcin_2048 Lcin_2947	Lsai_3469	Lsan_3903	Lgra_0968 Lgra_1039 Lgra_1944 Lgra_3368
LLO_2109	-	**F-box**	312	37.4	2.040	0.9762	3	Lcin_1020 Lcin_2048 Lcin_2947	Lsai_3469	Lsan_3903	Lgra_0968 Lgra_1039 Lgra_1944 Lgra_3368
LLO_2209	LLO_2210 - SidE homology (DUB)	-	558	39.5	-5.100	0.0340	2	-	Lsai_0397	-	-
LLO_2240	LLO_2238 - Lem27	**Coiled-coil**	163	29.9	-5.339	0.1251	5	Lcin_2949	Lsai_0428	Lsan_3152	Lgra_2470
LLO_2329 - RleC	LLO_2327 - SH2	**Ras GTPase**	330	30.5	2.283	0.6369	2	-	-	-	Lgra_0861
LLO_2440	LLO_2439 - sdhB	**STK**	593	32.1	0.550	1.0000	4	Lcin_2244 Lcin_2503 Lcin_2761	Lsai_0081	Lsan_1904 Lsan_3051	Lgra_1757
LLO_2558	-	-	251	36.8	2.838	0.9617	4	Lcin_2353	Lsai_0716	Lsan_1873	-
LLO_2746	-	**HAD and Coiled-coil**	344	36.9	4.399	0.7995	2	Lcin_1968	-	-	-
LLO_3042	-	**Colied coil**	502	31.7	2.479	0.8992	2	-	Lsai_1990	-	-
LLO_3086	-	**Colied coil**	430	35.6	5.511	0.9843	4	Lcin_3436	Lsai_1862	Lsan_1213	-
LLO_3245	-	Dymeclin	462	38.5	2.808	0.5962	1	-	-	-	-
LLO_3386	-	-	461	33.6	-0.564	0.9980	3	-	Lsai_2349	Lsan_2722	-
LLO_4077	LLO_2809 - Coiled-coil	-	65	34.8	-5.928	0.0122	1	-	-	-	-

Locus tages marked in red include the LelB regulatory element

Hight signal scores (>5) and machine learning scores (>0.95) are marked in red

Genes with %GC different (±4%) from the genomic GC content are marked in red:

L. longbeachae	37.1%
L. sainthelensi	37.1%
L. santicrucis	36.7%
L. cincinnatiensis	36.8%
L. gratiana	36.8%

Common effector domains are marked in blue:

STK	serine/threonine protein kinase
PCRF	Peptide chain release factor
PPR	Pentatricopeptide repeat
IcmD	Not in the Icm/Dot region
Dymeclin	Peripheral membrane protein dynamically associated with the Golgi apparatus
SH2	Src homology domain
IcmD (DotP) domain	This protein harbors an IcmD domain, but it is not the IcmD protein found in the Icm/Dot pathogenesis region II

Dataset S2B. Properties of *L. sainthelensi* proteins encoded by genes harboring the LelB regulatory element

Locus tag	Neighaboring genes	Effector domains	Size (AA)	%GC	Signal score	Machine learning score	No. of sp. with site	Llng	Lcin	Lsan	Lgra
Lsai_0081	Lsai_0079 - SdhB	**STK**	601	32.8	5.490	1.0000	4	LLO_1169 LLO_2440	Lcin_2244 Lcin_2503 Lcin_2761	Lsan_1904 Lsan_3051	Lgra_1757
Lsai_0267	Lsai_0268 - RleA (Rho GTPase)	**Ankyrin repeat**	343	34.3	-5.816	0.4897	3	LLO_1715	Lcin_0001 Lcin_0069	Lsan_2172 Lsan_2173	Lgra_2006
Lsai_0288	-	-	355	34.4	6.685	1.0000	4	LLO_1737	Lcin_0244	Lsan_3541	-
Lsai_0397	-	-	558	38.8	-2.640	0.3413	2	LLO_2209	-	-	-
Lsai_0428	Lsai_0426 - Lem27	**Coiled-coil**	184	29.7	-5.015	0.1518	5	LLO_2240	Lcin_2949	Lsan_3152	Lgra_2470
Lsai_0488	Lsai_0489 - lpg1751	-	543	31.6	6.005	0.9997	2	-	-	-	Lgra_3177
Lsai_0542	-	**Ankyrin repeat**	765	33.3	-1.677	0.9737	1	-	-	-	-
Lsai_0659	Lsai_0658 - SidG domain	Cysteine protease	318	31.6	4.951	0.9982	1	LLO_2165	Lcin_2511	Lsan_3412	Lgra_1110
Lsai_0676	Lsai_0674 - Ank	**RalF (GEF)**	387	30.0	8.569	0.9977	3	LLO_1397	-	Lsan_2248	-
Lsai_0716	-	-	255	38.7	5.213	0.9623	4	LLO_2558 LLO_2006	Lcin_2353	Lsan_1873	-
Lsai_0772	Lsai_0771 - Ank	**STK**	599	36.2	9.093	0.9985	3	LLO_0038	Lcin_0283	Lsan_3518	-
Lsai_0823	-	**Ankyrin repeat**	817	40.7	0.955	0.7086	4	LLO_0087 LLO_1894	Lcin_0957 Lcin_3077	Lsan_0225 Lsan_1808 Lsan_2700 Lsan_3490	Lgra_2953
Lsai_0828	-	**U-Box**	276	40.1	1.728	0.3108	1	LLO_2979	-	-	Lgra_0482
Lsai_0927	Lsai_0928 - Ank	-	722	34.7	3.733	0.9997	2	LLO_0245	-	-	-
Lsai_1210	-	**STK**	582	35.2	6.418	1.0000	3	LLO_0038	Lcin_0283	Lsan_3518	-
Lsai_1466	-	**Ankyrin repeat**	554	36.0	-1.004	0.3589	2	LLO_0990	-	-	-
Lsai_1492	-	Methyltransferases	650	37.2	5.438	0.7248	1	-	-	-	-
Lsai_1671	-	**Coiled-coil**	687	32.0	5.218	1.0000	1	LLO_1307	-	-	-
Lsai_1673	-	**Coiled-coil**	413	36.8	3.875	1.0000	1	-	-	-	-
Lsai_1773	-	-	466	37.5	-5.284	0.0337	3	-	Lcin_1066	Lsan_4005	-
Lsai_1837	-	-	67	38.2	0.521	0.1752	1	-	-	-	-
Lsai_1862	-	**Coiled-coil**	424	36.5	5.988	0.9699	4	LLO_3086	Lcin_3436	Lsan_1213	-
Lsai_1967	Lsai_1966 - Ank	**Coiled-coil**	762	36.1	4.787	0.9997	1	-	-	-	-
Lsai_1990	-	**Coiled-coil**	523	32.3	4.450	0.9995	2	LLO_3042	-	-	-
Lsai_1991	-	-	517	32.8	6.055	0.9564	1	-	-	-	Lgra_2243
Lsai_2153	Lsai_2154 - SidE domain	-	382	31.4	1.601	0.9959	1	LLO_3007	-	-	-
Lsai_2349	-	-	945	35.8	7.959	1.0000	3	LLO_3386	-	Lsan_2722	-
Lsai_2426	Lsai_2428 - LnaB	-	901	36.2	0.404	0.9960	1	LLO_4094	-	-	-
Lsai_2658	-	**Coiled-coil**	646	34.2	2.646	1.0000	3	LLO_2809	Lcin_2027	Lsan_3805	Lgra_2687
Lsai_2857	-	Protease	1556	36.7	0.999	0.3256	2	-	Lcin_3034	-	-
Lsai_2881	-	IcmD (DotP) domain	133	37.6	-0.850	0.0002	4	LLO_1060	Lcin_1547	Lsan_3057	Lgra_2049
Lsai_2938	-	-	610	33.6	5.463	1.0000	4	LLO_1123	Lcin_2397	Lsan_0856	-
Lsai_3224	-	**Ankyrin repeat**	432	31.4	-0.922	0.9997	2	LLO_2847	Lcin_0276	-	Lgra_2656
Lsai_3463	-	-	306	33.4	5.775	1.0000	3	LLO_0656	Lcin_0614	-	Lgra_1359
Lsai_3469	-	**F-box**	328	37.2	1.361	0.9745	3	LLO_1365 LLO_1995 LLO_2109 LLO_2144	Lcin_1020 Lcin_2048 Lcin_2947	Lsan_3903	Lgra_0968 Lgra_1039 Lgra_1944 Lgra_3368
Lsai_3483	-	PPR repeat	470	33.6	-5.438	0.0150	5	LLO_1404	Lcin_0095	Lsan_3243	Lgra_0492

Dataset S2C. Properties of *L. santicrucis* proteins encoded by genes harboring the LelB regulatory element

Locus tag	Neighaboring genes	Effector domains	Size (AA)	%GC	Signal score	Machine learning score	No. of sp. with site	Llng	Lcin	Lsai	Lgra
Lsan_0225	-	**Ankyrin repeat**	856	40.2	1.487	0.8549	4	LLO_0087 LLO_1894	Lcin_0957 Lcin_3077	Lsai_0823 Lsai_0156	Lgra_2953
Lsan_0813	-	Cupin	162	28.4	-8.830	0.0849	2	-	Lcin_0754	-	-
Lsan_0856	-	**Coiled-coil**	639	33.4	7.566	1.0000	4	LLO_1123	Lcin_2397	Lsai_2938	-
Lsan_1213	Lsan_1212 - F-box	**Colied coil**	314	35.2	6.050	0.8916	4	LLO_3086	Lcin_3436	Lsai_1862	-
Lsan_1632	-	**Colied coil**	782	34.8	10.954	1.0000	3	LLO_0614	Lcin_0210	Lsai_1515	Lgra_0790
Lsan_1808	-	**Ankyrin repeat**	816	40.8	7.233	0.8975	4	LLO_0087 LLO_1894	Lcin_0957 Lcin_3077	Lsai_0823 Lsai_0156	Lgra_2953
Lsan_1835	-	Acetyltransferase (GNAT)	315	33.2	-0.026	0.9085	1	-	-	-	-
Lsan_1873	-	-	260	37.3	4.213	0.9747	4	LLO_2558 LLO_2006	Lcin_2353	Lsai_0716	-
Lsan_1904	Lsan_1906 - SdhB	**STK**	607	33.0	0.524	1.0000	4	LLO_1169 LLO_2440	Lcin_2244 Lcin_2503 Lcin_2761	Lsai_0081	Lgra_1757
Lsan_1926	Lsan_1928 - Coiled-coil	-	529	40.0	9.219	0.9960	1	-	Lcin_0016	Lsai_0212	-
Lsan_1942	-	-	565	34.6	8.664	0.9945	1	LLO_1184 LLO_1619	-	Lsai_2232	Lgra_1206
Lsan_2248	Lsan_2250 - Ank	**RalF (GEF)**	389	30.2	3.883	0.9967	3	LLO_1397	-	Lsai_0676	-
Lsan_2656	Lsan_2657 - STK	-	486	35.5	1.638	0.5094	1	-	-	-	-
Lsan_2722	-	-	1264	33.6	3.256	0.9975	3	LLO_3386	-	Lsai_2349	-
Lsan_3057	-	IcmD (DotP) domain	133	37.3	-1.764	0.0001	4	LLO_1060	Lcin_1547	Lsai_2881	Lgra_2049
Lsan_3152	Lsan_3150 - Lem27	-	163	27.8	-5.014	0.2209	5	LLO_2240	Lcin_2949	Lsai_0428	Lgra_2470
Lsan_3243	-	PPR repeat	471	34.5	-6.766	0.0170	5	LLO_1404	Lcin_0095	Lsai_3483	Lgra_0492
Lsan_3284	Lsan_3286 - PieE	**Rab GTPase (LepB)**	522	35.1	6.283	0.9874	2	LLO_1454	-	-	-
Lsan_3301	-	**Coiled-coil**	173	36.6	6.263	0.9930	3	LLO_1473	Lcin_0370	-	-
Lsan_3490	-	**Ankyrin repeat**	1074	39.3	5.528	0.9807	4	LLO_0087 LLO_1894	Lcin_0957 Lcin_3077	Lsai_0823 Lsai_0156	Lgra_2953
Lsan_3518	-	**STK**	582	35.1	8.479	1.0000	3	LLO_0038	Lcin_0283	Lsai_0772 Lsai_0637 Lsai_1210	-
Lsan_3541	-	-	359	33.9	4.515	1.0000	4	LLO_1737	Lcin_0244	Lsai_0288	-
Lsan_3545	Lsan_3546 - Ank	**Coiled-coil**	847	35.1	7.809	1.0000	2	LLO_1741	-	-	-
Lsan_3616	-	**Coiled-coil**	190	33.9	3.877	0.9992	1	LLO_0177	-	Lsai_0857	-
Lsan_3805	-	**Coiled-coil**	820	32.8	-0.792	1.0000	3	LLO_2809	Lcin_2027	Lsai_2658	Lgra_2687
Lsan_4005	-	-	468	38.2	-5.917	0.0035	3	-	Lcin_1066	Lsai_1773	-
Lsan_4087	-	-	572	34.3	0.891	0.9909	2	LLO_0706	-	-	-
Lsan_4176	-	**SidC N-terminal**	968	35.8	1.987	1.0000	2	LLO_1372	-	-	-

Dataset S2D. Properties of *L. cincinnatiensis* proteins encoded by genes harboring the LelB regulatory element

Locus tag	Neighaboring genes	Effector domains	Size (AA)	%GC	Signal score	Machine learning score	No. of sp. with site	Llng	Lsai	Lsan	Lgra
Lcin_0095	Lcin_0096 - CetLl3	PPR repeat	471	35.2	-8.581	0.0174	5	LLO_1404	Lsai_3483	Lsan_3243	Lgra_0492
Lcin_0210	-	**Coiled-coil**	783	35.2	7.314	1.0000	3	LLO_0614	Lsai_1515	Lsan_1632	Lgra_0790
Lcin_0244	-	-	379	33.2	3.715	0.9995	4	LLO_1737	Lsai_0288	Lsan_3541	-
Lcin_0276	-	**Ankyrin repeat**	415	31.4	6.297	1.0000	2	LLO_2847	Lsai_3224	-	Lgra_2656
Lcin_0370	-	**Coiled-coil**	165	37.6	6.799	0.9922	3	LLO_1473	-	Lsan_3301	-
Lcin_0588	-	-	71	32.9	-6.153	0.0197	1	-	-	-	-
Lcin_0614	-	**Coiled-coil**	305	33.0	7.091	1.0000	3	LLO_0656	Lsai_3463	-	Lgra_1359
Lcin_0754	-	Cupin	177	28.1	-4.336	0.1086	2	-	-	Lsan_0813	-
Lcin_0957	Lcin_0956 - Coiled-coil	**Ankyrin repeat**	817	41.3	3.723	0.7853	4	LLO_0087 LLO_1894	Lsai_0823 Lsai_0156	Lsan_0225 Lsan_1808 Lsan_2700 Lsan_3490	Lgra_2953
Lcin_1020	-	**F-box**	316	36.3	-0.058	0.9772	3	LLO_1365 LLO_1995 LLO_2109 LLO_2144	Lsai_3469	Lsan_3903	Lgra_0968 Lgra_1039 Lgra_1944 Lgra_3368
Lcin_1052	-	-	295	39.1	-0.027	0.9827	2	LLO_3441	Lsai_1705	Lsan_3989	Lgra_2035
Lcin_1066	-	-	466	36.8	-3.014	0.0289	3	-	Lsai_1773	Lsan_4005	-
Lcin_1968	Lcin_1966 - PH domain	**HAD and Coiled-coil**	344	36.0	4.102	0.7765	2	LLO_2746	-	-	-
Lcin_2244	Lcin_2242 - SdhB	**STK**	601	32.9	6.656	1.0000	4	LLO_1169 LLO_2440	Lsai_0081	Lsan_1904 Lsan_3051	Lgra_1757
Lcin_2339	-	-	186	41.5	2.004	0.6598	2	-	-	-	Lgra_2260
Lcin_2353	-	-	257	38.6	4.404	0.9692	4	LLO_2558 LLO_2006	Lsai_0716	Lsan_1873	-
Lcin_2397	-	**Coiled-coil**	588	33.4	2.086	1.0000	4	LLO_1123	Lsai_2938	Lsan_0856	-
Lcin_2935	Lcin_2934 - STK	-	156	29.7	-2.008	0.9923	1	-	-	-	-
Lcin_2947	-	**F-box**	310	40.6	-0.703	0.9768	3	LLO_1365 LLO_1995 LLO_2109 LLO_2144	Lsai_3469	Lsan_3903	Lgra_0968 Lgra_1039 Lgra_1944 Lgra_3368
Lcin_2949	Lcin_2951 - Lem27	**Coiled-coil**	160	28.6	-4.993	0.2015	5	LLO_2240	Lsai_0428	Lsan_3152	Lgra_2470
Lcin_3034	-	Protease	1564	37.7	4.743	0.7952	2	-	Lsai_2857	-	-
Lcin_3077	-	**Ankyrin repeat**	551	36.7	9.830	0.9548	4	LLO_0087 LLO_1894	Lsai_0823 Lsai_0156	Lsan_0225 Lsan_1808 Lsan_2700 Lsan_3490	Lgra_2953
Lcin_3214	Lcin_3215 - Coiled-coil	**Ankyrin repeat**	76	32.5	-3.417	0.1593	1	-	-	-	-
Lcin_3436	Lcin_3435 - Coiled-coil	**Coiled-coil**	317	35.6	5.468	0.8553	4	LLO_3086	Lsai_1862	Lsan_1213	-

Dataset S2E. Properties of *L. gratiana* proteins encoded by genes harboring the LelB regulatory element

Locus tag	Neighaboring genes	Effector domains	Size (AA)	%GC	Signal score	Machine learning score	No. of sp. with site	Llng	Lcin	Lsai	Lsan
Lgra_0289	Lgra_0288 - Lem17	-	70	27.2	-6.867	0.1953	1	-	-	-	-
Lgra_0492	Lgra_0493 - RavZ- C-ter	PPR repeat	470	35.7	-1.652	0.0814	5	LLO_1404	Lcin_0095	Lsai_3483	Lsan_3243
Lgra_0649	-	SidG	661	34.0	-1.125	0.9997	1	LLO_4073	-	Lsai_0658 Lsai_3300	Lsan_0438 Lsan_1105 Lsan_1299
Lgra_0861	-	Ras-GTPase	295	31.4	-1.456	0.5875	1	LLO_2329	-	-	-
Lgra_0977	-	Cupin	306	29.1	-4.538	0.0637	1	-	Lcin_0899	-	-
Lgra_1152	-	Cupin	343	31.5	-5.494	0.0542	1	-	Lcin_0899	-	-
Lgra_1359	Lgra_1358 - acetyltransferase	Coiled-coil	310	32.8	6.623	1.0000	3	LLO_0656	Lcin_0614	Lsai_3463	-
Lgra_1713	-	Coiled-coil	530	33.1	7.893	1.0000	1	LLO_2996	Lcin_1796	Lsai_2166	Lsan_0164
Lgra_1745	-	-	262	33.3	5.820	0.9461	1	-	-	-	-
Lgra_2006	Lgra_2007 - RleA (Rho GTPase)	Ankyrin repeat	346	35.2	-7.071	0.4368	3	LLO_1715	Lcin_0001 Lcin_0069	Lsai_0267	Lsan_2172 Lsan_2173
Lgra_2035	-	-	259	41.9	-0.860	0.9812	2	LLO_3441	Lcin_1052	Lsai_1705	Lsan_3989
Lgra_2049	-	IcmD (DotP) domain	133	39.8	-2.739	0.0000	4	LLO_1060	Lcin_1547	Lsai_2881	Lsan_3057
Lgra_2129	-	Coiled-coil	249	32.0	3.215	0.8778	1	-	-	-	-
Lgra_2260	-	-	185	37.3	4.127	0.7543	2	-	Lcin_2339	-	-
Lgra_2446	-	-	301	34.7	-1.047	0.9879	1	-	-	-	-
Lgra_2470	Lgra_2472 - Lem27	Coiled-coil	164	28.7	-4.516	0.2061	5	LLO_2240	Lcin_2949	Lsai_0428	Lsan_3152
Lgra_2687	-	Coiled-coil	658	33.2	3.300	0.9997	3	LLO_2809	Lcin_2027	Lsai_2658	Lsan_3805
Lgra_2869	-	SidJ	707	34.2	-4.337	0.9975	1	LLO_0802 LLO_3096	Lcin_1682	Lsai_1871	Lsan_1200
Lgra_3177	-	-	481	30.8	2.292	0.9999	2	-	-	Lsai_0488	-

Dataset S3: Strains used in this study

**Strain name**	**Feature(s)**	**Reference or source**
Legionella pneumophila		
JR32	Homogeneous salt-sensitive isolate of Philadelphia-1 Sm^r r-, m+	1
GS3011	JR32 *icmT* ::Km	2
MG2138	JR32 lpg2138::Km	This study
NS1796	JR32 lpg1796::Km	This study
YSF-rpoS	JR32 *rpoS* ::Km	3
ZT-Fis1	JR32 *fis1* ::Km	4
ZT-Fis3	JR32 *fis3* ::Km	4
**Other *Legionella species***		
L. longbeachae	ATCC33484	ATCC
L. cincinnatiensis	ATCC43753	ATCC
L. gormanii	NCTC11401	NCTC
Escherichia coli		
MC1022	*araD139* Δ(*ara leu*)*7697* Δ(*lacZ*)*M15 galU galK strA*	5
MC1061	*araD139* Δ*(ara leu*)*7697* Δ*lacX74 galU galK strA*	6
BL21(DE3)	Δ*lon* , Δ*ompT* , T7 polymerase under *lacUV5* promoter	7

1. Sadosky AB, Wiater LA, and Shuman HA. 1993. Identification of *Legionella pneumophila* genes required for growth within and killing of human macrophages. Infect Immun 61: 5361-5373.

2. Segal G, and Shuman HA. 1998. Intracellular multiplication and human macrophage killing by *Legionella pneumophila* are inhibited by conjugal components of IncQ plasmid RSF1010. Mol. Microbiol. 30:197-208.

3. Shapira N, Zusman T, and Segal G. 2024. The LysR-type transcriptional regulator LelA co-regulates various effectors in different *Legionella* species. Mol. Microbiol. 121:243–259

4. Zusman T, Speiser Y, and Segal G. 2014. Two Fis regulators directly repress the expression of numerous effector-encoding genes in *Legionella pneumophila*. J Bacteriol 196:4172-4183.

5. Casadaban MJ, Chou J, and Cohen SN. 1980. In vitro gene fusions that join an enzymatically active beta-galactosidase segment to amino-terminal fragments of exogenous proteins: *Escherichia coli* plasmid vectors for the detection and cloning of translational initiation signals. J Bacteriol 143: 971-980.

6. Casadaban MJ, and Cohen SN. 1980. Analysis of gene control signals by DNA fusion and cloning in *Escherichia coli*. J Mol Biol 138: 179-207.

7. Studier FW, and Moffatt BA. 1986. Use of bacteriophage T7 RNA polymerase to direct selective high-level expression of cloned genes. J Mol Biol. 189:113-130.

Dataset S4: Plasmids used in this study

locus tag	Gene	Plasmids	Description	Reference
lacZ* fusions from *L. pneumohila				
lpg1101	*lem4*	pZT-lpg1101-lacZ	The regulatory region of *lem4* in pGS-lac-02	1
lpg1797	*rvfA*	pML-lpg1797-LacZ	The regulatory region of *legL6* in pGS-lac-02	This study
lpg2137	*legK2*	pZT-lpg2137-lacZ	The regulatory region of *legK2* in pGS-lac-02	1
lpg2138	*lelB*	pCA-lpg2138-lacZ	The regulatory region of *lelB* in pGS-lac-02	This study
lpg1227	*vpdB*	pCH-lpg1227-lacZ	The regulatory region of *vpdB* in pGS-lac-02	1
lpg1796	*lelC*	pCA-lpg1796-lacZ	The regulatory region of *lelC* in pGS-lac-02	This study
lpg2556	*regK3*	pZT-lpg2556-lacZ	The regulatory region of *regK3* in pGS-lac-02	1
lpg2830	*lubX*	pZT-lpg2830-lacZ	The regulatory region of *lubX* in pGS-lac-02	1
lacZ* fusions from *L. longbeachae				
LLO_1957		pCA-LLO_1957-lacZ	The regulatory region of LLO_1957 in pGS-lac-02	This study
LLO_0038		pCA-LLO_0038-lacZ	The regulatory region of LLO_0038 in pGS-lac-02	This study
LLO_0087		pCA-LLO_0087-lacZ	The regulatory region of LLO_0087 in pGS-lac-02	This study
LLO_0990		pCA-LLO_0990-lacZ	The regulatory region of LLO_0990 in pGS-lac-02	This study
LLO_1372		pCA-LLO_1372-lacZ	The regulatory region of LLO_1372 in pGS-lac-02	This study
LLO_1397	*ralF*	pCA-LLO_1379-lacZ	The regulatory region of LLO_1397 in pGS-lac-02	This study
LLO_1506	*cetLl4*	pCA-LLO_1506-lacZ	The regulatory region of LLO_1506 in pGS-lac-02	This study
LLO_1715		pCA-LLO_1715-lacZ	The regulatory region of LLO_1715 in pGS-lac-02	This study
LLO_2240		pCA-LLO_2240-lacZ	The regulatory region of LLO_2240 in pGS-lac-02	This study
LLO_2440		pCA-LLO_2440-lacZ	The regulatory region of LLO_2440 in pGS-lac-02	This study
LLO_3086		pCA-LLO_3086-lacZ	The regulatory region of LLO_3086 in pGS-lac-02	This study
lacZ* fusions from *L. cincinnatiensis				
Lcin_0370		pCA-Lcin_0370-lacZ	The regulatory region of Lcin_0370 in pGS-lac-02	This study
Lcin_2089		pCA-Lcin_2089-lacZ	The regulatory region of Lcin_2089 in pGS-lac-02	This study
Lcin_3436		pCA-Lcin_3436-lacZ	The regulatory region of Lcin_3436 in pGS-lac-02	This study
lacZ* fusions from *L. gormanii				
Lgor_0297		pCA-Lgor_0297-lacZ	The regulatory region of Lgor_0297 in pGS-lac-02	This study
Lgor_3248		pCA-Lgor_3248-lacZ	The regulatory region of Lgor_3248 in pGS-lac-02	This study
***lacZ* fusions containing a mutation in the lpg2138 regulatory element**				
lpg1101	*lem4*	pCA-lpg1101-TAT3-lacZ	pZT-lpg1101-lacZ with TAT to ATA mutation at its *lelB* regulatory element	This study
lpg1797	*rvfA*	pCA-lpg1797-TAT3-lacZ	pML-lpg1797-LacZ with TAT to ATA mutation at its *lelB* regulatory element	This study
lpg2137	*legK2*	pCA-lpg2137-TAT3-lacZ	pZT-lpg2137-lacZ with TAT to ATA mutation at its *lelB* regulatory element	This study
***lacZ* fusions containing mutations in the lpg1796 regulatory element**				
lpg1227	*vpdB*	pCA-lpg1227-LTTR-m1-lacZ	pCH-lpg1227-lacZ with GTT to CAA mutation at its *lelC* regulatory element	This study
LLO_1957		pCA-LLO_1957-LTTR-m1-lacZ	pCA-LLO_1957-lacZ with GTT to CAA mutation at its *lelC* regulatory element	This study
**Regulators under P*tac* control**				
lpg2138	*lelB*	pML-pMMB207c-Ptac-lpg2138	*lelB* in pMMB207C under P*tac* control	This study
		pCA-pMMB207c-Ptac-lpg2138-Km	pML-pMMB207c-Ptac-lpg2138 with the Km-cassette at the BamHI site	This study
lpg1796	*lelC*	pCA-pMMB207c-Ptac-lpg1796	*lelC* in pMMB207C under P*tac* control	This study
lpg1284	*rpoS*	pDT-pMMB-Ptac-rpoS	*rpoS* in pMMB207C under P*tac* control	2
LLO_1956		pCA-pMMB207c-Ptac-LLO_1956	LLO_1956 in pMMB207C under P*tac* control	This study
Lgor_0298		pCA-pMMB207c-Ptac-Lgor_0298	Lgor_0298 in pMMB207C under P*tac* control	This study

		pCA-pMMB207c-Ptac-Lgor_0298-Km	pCA-pMMB207c-Ptac-Lgor_0298 with the Km-cassette at the BamHI site	This study
Lgor_3249		pCA-pMMB207c-Ptac-Lgor_3249	Lgor_3249 in pMMB207C under P*tac* control	This study
		pCA-pMMB207c-Ptac-Lgor_3249-Km	pCA-pMMB207c-Ptac-Lgor_3249 with the Km-cassette at the BamHI site	This study
Lcin_1101		pCA-pMMB207c-Ptac-Lcin_1101	Lcin_1101 in pMMB207C under P*tac* control	This study
		pCA-pMMB207c-Ptac-Lcin_1101-Km	pCA-pMMB207c-Ptac-Lcin_1101 with the Km-cassette at the BamHI site	This study
Lcin_2090		pCA-pMMB207c-Ptac-Lcin_2090	Lcin_2090 in pMMB207C under P*tac* control	This study
		pCA-pMMB207c-Ptac-Lcin_2090-Km	pCA-pMMB207c-Ptac-Lcin_2090 with the Km-cassette at the BamHI site	This study
L. pneumophila lacZ **fusions containing P*tac*-regulator**				
lpg1101	*lem4*	pCA-Ptac-lpg2138-Km-lpg1101-lacZ	*lacI* and P*tac*-*lelB* in pZT-lpg1101-lacZ	This study
		pCA-Ptac-lpg2138-Km-lpg1101-TAT3-lacZ	*lacI* and P*tac*-*lelB* in pCA-lpg1101-TAT3-lacZ	This study
lpg1797	*rvfA*	pCA-Ptac-lpg2138-Km-lpg1797-lacZ	*lacI* and P*tac*-*lelB* in pML-lpg1797-LacZ	This study
		pCA-Ptac-lpg2138-km-lpg1797-TAT3-lacZ	*lacI* and P*tac*-*lelB* in pCA-lpg1797-TAT3-lacZ	This study
		pCA-Ptac-lpg1796-lpg1797-lacZ	*lacI* and P*tac*-*lelC* in pML-lpg1797-LacZ	This study
lpg2137	*legK2*	pMG-Ptac-lpg2138-lpg2137-lacZ	*lacI* and P*tac*-*lelB* in pZT-lpg2137-lacZ	This study
		pCA-Ptac-lpg2138-km-lpg2137-TAT3-lacZ	*lacI* and P*tac*-*lelB* in pCA-lpg2137-TAT3-lacZ	This study
lpg1227	*vpdB*	pCA-Ptac-lpg1796-lpg1227-lacZ	*lacI* and P*tac*-*lelC* in pCH-lpg1227-lacZ	This study
		pCA-Ptac-lpg1796-lpg1227-LTTR-m1-lacZ	*lacI* and P*tac*-*lelC* in pCA-lpg1227-LTTR-m1-lacZ	This study
		pCA-Ptac-lpg2138-Km-lpg1227-lacZ	*lacI* and P*tac*-*lelB* in pCH-lpg1227-lacZ	This study
lpg2138	*lelB*	pCA-Ptac-rpoS-lpg2138-lacZ	*lacI* and P*tac*-*rpoS* in pCA-lpg2138-lacZ	This study
lpg1796	*lelC*	pCA-Ptac-rpoS-lpg1796-lacZ	*lacI* and P*tac*-*rpoS* in pCA-lpg1796-lacZ	This study
lpg2556	*regK3*	pCA-Ptac-lpg2138-Km-regK3-lacZ	*lacI* and P*tac*-*lelB* in pZT-lpg2556-lacZ	This study
		pCA-Ptac-lpg1796-regK3-lacZ	*lacI* and P*tac*-*lelC* in pZT-lpg2556-lacZ	This study
lpg2830	*lubX*	pCA-Ptac-lpg2138-Km-lubX-lacZ	*lacI* and P*tac*-*lelB* in pZT-lpg2830-lacZ	This study
		pCA-Ptac-lpg1796-lubX-lacZ	*lacI* and P*tac*-*lelC* in pZT-lpg2830-lacZ	This study
L. longbeachae lacZ **fusions containing P*tac*- regulators**				
LLO_1957		pCA-Ptac-lpg1796-LLO_1957-lacZ	*lacI* and P*tac*-*lelC* in pCA-LLO_1957-lacZ	This study
		pCA-Ptac-lpg1796-LLO_1957-LTTR-m1-lacZ	*lacI* and P*tac*-*lelC* in pCA-LLO_1957-LTTR-m1-lacZ	This study
		pCA-Ptac-LLO_1956-LLO_1957-lacZ	*lacI* and P*tac*-LLO_1956 in pCA-LLO_1957-lacZ	This study
LLO_0038		pCA-Ptac-lpg2138-Km-LLO_0038-lacZ	*lacI* and P*tac*-*lelC* in pCA-LLO_0038-lacZ	This study
LLO_0087		pCA-Ptac-lpg2138-Km-LLO_0087-lacZ	*lacI* and P*tac*-*lelC* in pCA-LLO_0087-lacZ	This study
LLO_0990		pCA-Ptac-lpg2138-Km-LLO_0990-lacZ	*lacI* and P*tac*-*lelC* in pCA-LLO_0990-lacZ	This study
LLO_1372		pCA-Ptac-lpg2138-Km-LLO_1372-lacZ	*lacI* and P*tac*-*lelC* in pCA-LLO_1372-lacZ	This study
LLO_1397	*ralF*	pCA-Ptac-lpg2138-Km-LLO_1397-lacZ	*lacI* and P*tac*-*lelC* in pCA-LLO_1397-lacZ	This study
LLO_1506	*cetLl4*	pCA-Ptac-lpg2138-Km-LLO_1506-lacZ	*lacI* and P*tac*-*lelC* in pCA-LLO_1506-lacZ	This study
LLO_1715		pCA-Ptac-lpg2138-Km-LLO_1715-lacZ	*lacI* and P*tac*-*lelC* in pCA-LLO_1715-lacZ	This study
LLO_2240		pCA-Ptac-lpg2138-Km-LLO_2240-lacZ	*lacI* and P*tac*-*lelC* in pCA-LLO_2240-lacZ	This study
LLO_2440		pCA-Ptac-lpg2138-Km-LLO_2440-lacZ	*lacI* and P*tac*-*lelC* in pCA-LLO_2440-lacZ	This study
LLO_3086		pCA-Ptac-lpg2138-Km-LLO_3086-lacZ	*lacI* and P*tac*-*lelC* in pCA-LLO_3086-lacZ	This study
L. cincinnatiensis lacZ **fusions containing P*tac*- regulators**				
Lcin_0370		pCA-Ptac-Lcin_1101-Km-Lcin_0370-lacZ	*lacI* and P*tac*-Lcin_1101 in pCA-Lcin_0370-lacZ	This study
		pCA-Ptac-Lcin_2090-Km-Lcin_0370-lacZ	*lacI* and P*tac*-Lcin_2090 in pCA-Lcin_0370-lacZ	This study
Lcin_2089		pCA-Ptac-Lcin_1101-Km-Lcin_2089-lacZ	*lacI* and P*tac*-Lcin_1101 in pCA-Lcin_2089-lacZ	This study
		pCA-Ptac-Lcin_2090-Km-Lcin_2089-lacZ	*lacI* and P*tac*-Lcin_2090 in pCA-Lcin_2089-lacZ	This study
Lcin_3436		pCA-Ptac-Lcin_1101-Km-Lcin_3436-lacZ	*lacI* and P*tac*-Lcin_1101 in pCA-Lcin_3436-lacZ	This study
		pCA-Ptac-Lcin_2090-Km-Lcin_3436-lacZ	*lacI* and P*tac*-Lcin_2090 in pCA-Lcin_3436-lacZ	This study

***L. gormanii lacZ* fusions containing P*tac*- regulators**				
Lgor_0297		pCA-Ptac-Lgor_0298-Km-Lgor_0297-lacZ	*lacI* and P*tac* -Lgor_0298 in pCA-Lgor_0297-lacZ	This study
		pCA-Ptac-Lgor_3249-Km-Lgor_0297-lacZ	*lacI* and P*tac* -Lgor_3249 in pCA-Lgor_0297-lacZ	This study
Lgor_3248		pCA-Ptac-Lgor_3249-Km-Lgor_3248-lacZ	*lacI* and P*tac* -Lgor_3249 in pCA-Lgor_3248-lacZ	This study
		pCA-Ptac-Lgor_0298-Km-Lgor_3248-lacZ	*lacI* and P*tac* -Lgor_0298 in pCA-Lgor_3248-lacZ	This study
**His tagged regulators**				
lpg2138	*lelB*	pCA-pET21a-lpg2138-his	*lelB* in pET-21a	This study
lpg1796	*lelC*	pCA-pET21a-lpg1796-his	*lelC* in pET-21a	This study
**Construction of deletion mutants**				
lpg2138	*lelB*	pMG-pUC18-lpg2138 UP	The upstream region of *lelB* in pUC-18	This study
		pMG-pUC18-lpg2138 DW	The downstream region of *lel* B in pUC-18	This study
		pMG-pUC18-lpg2138-UP-Km-DW	The upstream and downstream region of *lelB* with the Km-cassette between them in pUC-18	This study
		pMG-pLAW344-UP-Km-DW-lpg2138	The insert of pMG-pUC18-lpg2138-UP-Km-DW in pLAW344	This study
lpg1796	*lelC*	pNS-lpg1796-UP	The upstream region of *lelC* in pUC-18	This study
		pNS-lpg1796-DW	The downstream region of *lelC* in pUC-18	This study
		pNS-lpg1796-km	The upstream and downstream region of *lelC* with the Km-cassette between them in pUC-18	This study
		pNS-lpg1796-pLAW-km	The insert of pNS-lpg1796-km in pLAW344	This study
**Vectors**				
		pMMB207C	oriV(RSF1010) *IncQ* Δ*mobA* , $lacI^q$ Cm^r P*tac* *oriT* MCS	3
		pGS-lac-02	oriV(RSF1010) with a promoterless *lacZ* gene Cm^r	4
		pUC-18	oriR(colEI), MCS, Amp^r	5
		pLAW344	oriR(colEI) *sacB* MCS *oriT* (RK2) Cm^r Amp^r	6
		pET-21a	oriR(colEI) Amp^r, pT7, C-terminal His_6 tag	Novagen

1. Zusman T, Speiser Y, and Segal G. 2014. Two Fis regulators directly repress the expression of numerous effector-encoding genes in *Legionella pneumophila* . J Bacteriol 196:4172-4183.

2. Shapira N, Zusman T, and Segal G. 2024. The LysR-type transcriptional regulator LelA co-regulates various effectors in different *Legionella* species. Mol. Microbiol. 121:243–259

3. Charpentier X, Faucher SP, Kalachikov S, and Shuman HA. 2008. Loss of RNase R induces competence development in *Legionella pneumophila* . J Bacteriol 190:8126-8136.

4. Gal-Mor O, Zusman T, and Segal G. 2002. Analysis of DNA regulatory elements required for expression of the *Legionella pneumophila icm* and *dot* virulence genes. J Bacteriol 184:3823-3833.

5. Yanish-Perron C, Viera J, and Messing J. 1985. Improved M13 phage cloning vectors and host strains: nucleotide sequences of the M13mp18 and pUC19 vectors. Gene 33:103-119.

6. Sadosky AB, Wiater LA, and Shuman HA. 1993. Identification of *Legionella pneumophila* genes required for growth within and killing of human macrophages. Infect Immun 61: 5361-5373.

Dataset S5: Primers used in this study

lpg#	Gene	Plasmid name	Primer name	Sequence (5'-3')
lacZ* fusions from *L. pneumohila				
lpg1797	*rvfA*	pML-lpg1797-LacZ	Lpg1797-LacZ-EcoRI	CGACGAATTCTTTTAGTCATTTACGTTTGC
			Lpg1797-LacZ-BamHI	GACCGGATCCCCAGTATGAATAGTAATTGACATGATATG
lpg2138	*lelB*	pCA-lpg2138-lacZ	Lpg2138-lacZ-EcoRI	GCAGGAATTCTCCTACAAGCCAATTATCTACA
			Lpg2138-lacZ-BamHI	GACAGGATCCCCATTAATTTTTCTGAGATCCATTTTTCATT
lpg1796	*lelC*	pCA-lpg1796-lacZ	Lpg1796-lacZ-EcoRI	GCAGGAATTCACAAACGTATGGACTACCCG
			Lpg1796-lacZ-BamHI	GACAGGATCCCCTTGCAAATCGGCAATATTCAT
lacZ* fusions from *L. longbeachae				
LLO_1957		pCA-LLO_1957-lacZ	LLO1957-lacZ-EcoRI	GCAGGAATTCCAGAAGATATAAAAAGTTGAGCA
			LLO1957-lacZ-BamHI	GACAGGATCCCCTAATAATTCTGTAATTGTCATCATTTAACCT
LLO_0038		pCA-LLO_0038-lacZ	LLO_0038-lacZ-EcoRI	GCACGAATTCGCGCATGAATACACTCC
			LLO_0038-lacZ-BamHI	GACCGGATCCCCAGCAATTCGTTTGATACTCATAAT
LLO_0087		pCA-LLO_0087-lacZ	LLO_0087-lacZ-EcoRI	GCACGAATTCAAAATTAACTCCATTTACTTGCC
			LLO_0087-lacZ-BamHI	GACCGGATCCCCGATTTCGTTTAAAAAATGCATTGAATTA
LLO_0990		pCA-LLO_0990-lacZ	LLO_0990-lacZ-EcoRI	GCACGAATTCGCTTAGAGTACCTTTTCGTTCT
			LLO_0990-lacZ-BamHI	GACCGGATCCCCTTTCAGTCTTTTTTTTAGCATTAATAGCC
LLO_1372		pCA-LLO_1372-lacZ	LLO_1372-lacZ-EcoRI	GCACGAATTCACCAACCTACCTAGATCC
			LLO_1372-lacZ-BamHI	GACCGGATCCCCTGAAATTTTGAAACTAGACATCATACA
LLO_1397		pCA-LLO_1379-lacZ	LLO_1397-lacZ-EcoRI	GCACGAATTCTTAGAATCTCACCCCACTAC
			LLO_1397-lacZ-BamHI	GACCGGATCCCCTTCTAATTCTGGATTAATCATGATATACTC
LLO_1506		pCA-LLO_1506-lacZ	LLO_1506-lacZ-EcoRI	GCACGAATTCTTTTATTTTCCTGCTAAGTCTG
			LLO_1506-lacZ-BamHI	GACCGGATCCCCTTTTACAATTGGATCTCTCATTATCAC
LLO_1715		pCA-LLO_1715-lacZ	LLO_1715-lacZ-EcoRI	GCACGAATTCCAAAGTTGAATATCCCATGC
			LLO_1715-lacZ-BamHI	GACCGGATCCCCTAACTCGCTTTTTATTTCCATTTTATT
LLO_2240		pCA-LLO_2240-lacZ	LLO_2240-lacZ-EcoRI	GCCAGAATTCGATGAATTATAAACAATCCTGAAGAGA
			LLO_2240-lacZ-BamHI	GACCGGATCCAAGGTATCTAAACAACTCATTTTTTAT
LLO_2440		pCA-LLO_2440-lacZ	LLO_2440-lacZ-EcoRI	GCACGAATTCAGAATAATAAAGAGAATTAGGTAATATCCC
			LLO_2440-lacZ-BamHI	GACCGGATCCCCTACAAAAGATAAGTGATGCATGATAAA
LLO_3086		pCA-LLO_3086-lacZ	LLO_3086-lacZ-EcoRI	GCACGAATTCCTACCATAGGTGAAGAGC
			LLO_3086-lacZ-BamHI	GACAGGATCCCCAATTTGCTTTGCATTGGTCAT
lacZ* fusions from *L. cincinnatiensis				
Lcin_0370		pCA-Lcin_0370-lacZ	Lcin_0370-lacZ-EcoRI	GCACGAATTCCATCAACATGGGTTCACC
			Lcin_0370-lacZ-BamHI	GACCGGATCCCCGTTATGATTGATTTTTTTCATTTTCATAGTTC
Lcin_2089		pCA-Lcin_2089-lacZ	Lcin_2089-lacZ-EcoRI	GCAGGAATTCTTTTAAGAGACAAGACGTGC
			Lcin_2089-lacZ-BamHI	GACCGGATCCCCTATACATTGATATAAACTCATCGCTG
Lcin_3436		pCA-Lcin_3436-lacZ	Lcin_3436-lacZ-EcoRI	GCACGAATTCGTACAAAGGCTTCGAGA
			Lcin_3436-lacZ-BamHI	GACCGGATCCCCTTTTTTAGGAAAATTAGTCATTATTATCTCAAC
lacZ* fusions from *L. gormanii				
Lgor_0297		pCA-Lgor_0297-lacZ	Lgor_0297-lacZ-EcoRI	GCACGAATTCGGTTAACATGTTCTATCACAAACG
			Lgor_0297-lacZ-BamHI	GACCGGATCCCCTGTATCGTGTTTCATTTGCATAATTAAA
Lgor_3248		pCA-Lgor_3248-lacZ	Lgor_3248-lacZ-EcoRI	GCACGAATTCAACTCCAAAGAATCCATCATAC
			Lgor_3248-lacZ-BamHI	GACTGGATCCTGGTCCATCTTTATTTTCATCTTACT

***lacZ* fusions containing a mutation in the lpg2138 regulatory element**				
lpg1101	*lem4*	pCA-lpg1101-TAT3-lacZ	Lpg1101-Mut-TAT-3-F	TTAAAAATAATATGACTATAGAAAACAATTAATTGTACATAAACAAATCA
			Lpg1101-Mut-TAT-3-R	GTACAATTAATTGTTTTCTATAGTCATATTATTTTTAATAATATCCATTGTTTTTC
lpg1797	*rvfA*	pCA-lpg1797-TAT3-lacZ	Lpg1797-Mut-TAT-3-F	GTGATATGAGAATACAAGATATTCGATTGGTCAACT
			Lpg1797-Mut-TAT-3-R	CGAATATCTTGTATTCTCATATCACTTCTAGTAATAAATATTA
lpg2137	*legK2*	pCA-lpg2137-TAT3-lacZ	Lpg2137-Mut-TAT-3-F	AATTTAATAAAACAATACAAAACAATTAATTGTATTAATATTTATCA
			Lpg2137-Mut-TAT-3-R	CAATTAATTGTTTTGTATTGTTTTATTAAATTTAGTTATATCTATTATTTT
***lacZ* fusions containing a mutation in the lpg1796 regulatory element**				
lpg1227	*vpdB*	pCA-lpg1227-LTTR-m1-lacZ	Lpg1227-LTTR-mut-1F	CAACATAACTATAACAAATAACCTATAAAAAGAACTCATAAGCATGT
			Lpg1227-LTTR-mut-1R	CTTTTTATAGGTTATTTGTTATAGTTATGTTGATTGATTTACAGCA
LLO_1957		pCA-LLO_1957-LTTR-m1-lacZ	LLO_1957-LTTR-mut-1F	ATCAAACCATTGCAAATAACCTATAAAAACAAGTAATAGCATTC
			LLO_1957-LTTR-mut-1R	TTTTATAGGTTATTTGCAATGGTTTGATTTTGAACAATAATCA
**Regulators under P*tac* control**				
lpg2138	*lelB*	pML-pMMB207c-Ptac-lpg2138	Lpg2138-Ptac-EcoRI	GACAGAATTCATGGATCTCAGAAAAATTAATCTCA
			Lpg2138-Ptac-BamHI	GACTGGATCCTTAATATCAATTTGTTTAAGCC
lpg1796	*lelC*	pCA-pMMB207c-Ptac-lpg1796	Ptac-lpg1796-EI-F	GAGCGAATTCATGAATATTGCCGATTTGCAA
			Ptac-lpg1796-R	GAGCGGATCCCATTTTTAACTGGCAATAAAG
			Lpg1796-EI-mut-F	AATTAGCAGAATTTGATGCGGTGCTTATGGAAAA
			Lpg1796-EI-mut-R	GCACCGCATCAAATTCTGCTAATTCAGCAAAGG
LLO_1956		pCA-pMMB207c-Ptac-LLO_1956	Ptac-LLO1956-EI-F	GAGCGAATTCATGAATATTACCGACCTGCAATCC
			Ptac-LLO1956-Bam-R	GAGAGGATCCGTCAACTCGCCTTAATATTTTATTGG
			LLO1956-EI-mut-F	ATGATAATTTATATGAATAGTCTAGGCTTAAATTATTTTATCAACACTAA
			LLO1956-EI-mut-R	AATTTAAGCCTAGACTATTCATATAAATTATCATAGGAGGAACAATAG
Lgor_0298		pCA-pMMB207c-Ptac-Lgor_0298	Lgor_0298-Ptac-EcoRI	GACAGAATTCATGGACTTAAAAAAGGTAAATTTAAATTTATTGG
			Lgor_0298-Ptac-BamHI	GACTGGATCCGATTGAGGTTTTTAATTGGATGCG
Lgor_3249		pCA-pMMB207c-Ptac-Lgor_3249	Lgor_3249-Ptac-EcoRI	GACAGAATTCATGGACTTGAACCGTGTAA
			Lgor_3249-Ptac-BamHI	GACAGGATCCACAGTGTTTGTACATAATGAAGGG
			Lgor_3249-Mut-1F	CGCCTATCTTGCTCAACATGCCCCCAACTTTACC
			Lgor_3249-Mut-1R	GGGCATGTTGAGCAAGATAGGCGGATAATTTGG
			Lgor_3249-Mut-2F	CCTGTGCAATGCGTAAGTCACACCCTTTGGCTAGT
			Lgor_3249-Mut-2R	GGTGTGACTTACGCATTGCACAGGCCATCTC
Lcin_1101		pCA-pMMB207c-Ptac-Lcin_1101	Lcin_1101-Ptac-EcoRI	GACAGAATTCATGGATATAAGAAGAATTAATTTAAATTTATTAATTC
			Lcin_1101-Ptac-BamHI	GACTGGATCCTGTTAACCTACGGTGAAAAG
Lcin_2090		pCA-pMMB207c-Ptac-Lcin_2090	Lcin_2090-Ptac-EcoRI	GACAGAATTCATGGATATTCGAAAAATTAACCTTAATTTG
			Lcin_2090-Ptac-BamHI	GACTGGATCCGCCGAAAAATATTAAGCACG
			Lcin_2090-Mut-F	CGTTTCCCAATAAGGAATTTAGTATTTATTTTTTTTATCATATGCGATTAACT
			Lcin_2090-Mut-R	GATAAAAAAAATAAATACTAAATTCCTTATTGGGAAACGGAA
**His tagged regulators**				
lpg2138	*lelB*	pCA-pET21a-lpg2138-his	Lpg2138-pET-F-NdeI	GTCACATATGGATCTCAGAAAAATTAATCTCAAC
			Lpg2138-pET-BamHI	GACTGGATCCGCAGCCTCATGATTGACGCAAC
lpg1796	*lelC*	pCA-pET21a-lpg1796-his	Lpg1796-pET-F-NdeI	GTCACATATGAATATTGCCGATTTGCAA
			Lpg1796-pET-R-BamHI	GAGCGGATCCGCATTAACAAGGGAAAAAGAGCTT
			Lpg1796-mut-Nde-F	CCCACCACTTATAATCCACATGAACGCACTGGACTTGAA

			Lpg1796-mut-Nde-R	TCATGTGGATTATAAGTGGTGGGATAATAGTTTGAGCAATATATACTG
**Gel-shift probes**				
lpg1101	*lem4*		Lpg1101-GS-F	GATCTGCAACCCATATTTAGCTGAGCG
			Lpg1101-GS-R	GATCGATTATTTCCTCGCAAAACCACCC
lpg1797	*rvfA*		Lpg1797-GS-F	TGTTTCAAACAAAACATCAACAAAAACC
			Lpg1797-GS-R	TGATATGCTCTTGGTTAATGCAATAAA
lpg2137	*legK2*		Lpg2137-GS-F	ATAACTGCTCATAAAAAGACGTCA
			Lpg2137-GS-R	GCTCTCCTACAAGCCAATTATC
lpg1227	*vpdB*		Lpg1227-GS-F	CTGAGTAGTGTTGAATAAACTGATGC
			Lpg1227-GS-R	CCTGCTTATTGGGTATTTAATACATAGT
LLO_1957			LLO_1957-GS-F	CCATTTATCATAAGACTTAAGTTAATACTAGGAC
			LLO_1957-GS-R	CCTTCATGTTATTGGATTACTCAATATTATGAC
**Construction of deletion mutants**				
lpg2138	*lelB*	pMG-pUC18-lpg2138 UP	lpg2138-Up-BamHI	GACTGGATCCTTGAAAGTTAAATCGCCATGGG
			lpg2138-Up-SalI	GACAGTCGACGAGATTAATTTTTCTGAGATCCAT
		pMG-pUC18-lpg2138 DW	lpg2138-Dw-SalI	GACTGTCGACTATTGTTGCGTCAATCATGA
			lpg2138-Dw-EcoRI	GACTGAATTCTCTTTTACGAAATGGAATC
lpg1796	*lelC*	pNS-lpg1796-UP	lpg1796-Up-F	GACAGGATCCGCGATGAATCAATTCATTGC
			lpg1796-Up-R	GACTGTCGACATCGGCAATATTCATTATTTTAAAACC
		pNS-lpg1796-DW	lpg1796-Down-F	GACTGTCGACAGCTCTTTTTCCCTTGTTAATTAG
			lpg1796-Down-R	TGACGAATTCATGCTCTTGGTTAATGCAATAAA
